# Donor–Acceptor Viologens with Through‐Space Conjugation for Enhanced Visible‐Light‐Driven Photocatalysis

**DOI:** 10.1002/advs.202409925

**Published:** 2024-11-21

**Authors:** Yi Qiao, Liang Xu, Xiaoyang Liu, Yujing Gao, Naiyao Li, Yawen Li, Tianle Cao, Ni Yan, Zishun Liu, Gang He

**Affiliations:** ^1^ Frontier Institute of Science and Technology Key Laboratory of Thermo‐Fluid Science and Engineering of Ministry of Education State Key Laboratory for Strength and Vibration of Mechanical Structures Xi'an Jiaotong University Xi'an Shaanxi Province 710054 P. R. China; ^2^ School of Materials Science and Engineering Chang'an University Xi'an 710064 China; ^3^ International Center for Applied Mechanics State Key Laboratory for Strength and Vibration of Mechanical Structures Xi'an Jiaotong University Xi'an Shaanxi Province 710054 P. R. China; ^4^ City University of Hong Kong (Dongguan) Dongguan 523808 China

**Keywords:** donor–acceptor, hydrogen production, oxidative coupling reaction, through‐space conjugation, viologens

## Abstract

A series of novel donor–acceptor (D–A) type viologens with through‐space conjugation (TSC) are synthesized. The synergistic effect of D–A conjugation and TSC endow these compounds with a narrower energy gap, excellent redox properties, strong absorption in the visible range (up to 450 nm), and high efficiency of intramolecular charge transfer (ICT). These compounds also exhibit a long‐lived charge separation state and stable radical formation. Considering their impressive photophysical and redox properties, TSC viologens with D–A conjugation are explored for visible‐light‐driven photocatalysis. As a catalyst in oxidative coupling reactions under visible light, compound **9** can achieve a yield of 94%, attributed to its strong ICT effect. Additionally, hybridized into graphitic carbon nitride (g‐C₃N₄), compound **9** enhances hydrogen production performance under visible light, achieving an H_2_ generation rate of 4102 µmol h^−1^g^−1^. This study highlights the potential applications of TSC viologens in photocatalytic systems.

## Graphical abstract

By introducing the synergistic effect of donor–acceptor (D–A) conjugation and through‐space conjugation (TSC) into viologen, a series of novel D–A type viologens with TSC are synthesized, which exhibits a narrower energy gap, excellent redox properties, strong absorption in the visible range, high efficiency of intramolecular charge transfer (ICT) and a long‐lived charge separation state. TSC viologens with D–A conjugation showed impressive performance for visible‐light‐driven photocatalysis, which could not only improve the hydrogen production efficiency of g‐C3N4 but also achieve a yield of 94% as a catalyst in oxidative coupling reactions under visible light.

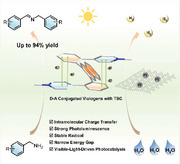

## 1 Introduction

Organic photoelectric materials have been widely applied in organic light‐emitting diodes,^[^ ^1^, ^2^, ^3^, ^4^, ^5^, ^6^, ^7^ ^]^ organic field‐effect transistors,^[^ ^8^, ^9^, ^10^, ^11^, ^12^, ^13^ ^]^ organic solar cells,^[^ ^14^, ^15^, ^16^, ^17^, ^18^, ^19^ ^]^ biomedical applications^[^ ^20^, ^21^, ^22^, ^23^, ^24^ ^]^ and other fields owing to their excellent properties. Viologens (MV^2+^), as one of the most representative organic photoelectric materials, taking advantage of good redox properties and excellent electron transfer ability, have been widely applied to electrochromic,^[^ ^25^, ^26^, ^27^, ^28^ ^]^ energy storage,^[^ ^29^, ^30^, ^31^ ^]^ photodynamic therapy^[^ ^32^, ^33^ ^]^ and photocatalysts.^[^ ^34^, ^35^, ^36^ ^]^ Nevertheless, the wide energy gap, poor visible light absorption, and low conjugation degree of traditional viologens restricted their further development and application greatly. The above shortcomings can be alleviated by many modification methods such as bridging of main group elements in the bipyridine bay and introducing conjugation groups into the viologen skeleton.^[^ ^37^, ^38^, ^39^, ^40^, ^41^ ^]^ However, it's still a challenge to avoid forming a neutral quinoid structure, which is unfavorable for the utilization of free radicals in photocatalysis. Through‐space conjugation (TSC) with π electron spatial delocalization possesses the ability of efficient multidimensional charge and energy transport, which have attracted numerous researchers by characteristic electronic and optical properties such as aggregation‐induced emission (AIE),^[^ ^42^, ^43^, ^44^, ^45^, ^46^ ^]^ thermally activated delayed fluorescence (TADF)^[^ ^47^, ^48^, ^49^, ^50^, ^51^ ^]^ and monomolecular conductance.^[^ ^52^, ^53^, ^54^, ^55^ ^]^ Recently, viologens with TSC were designed and synthesized by our group, which not only prevents the formation of a neutral quinoid structure but also retards charge recombination, showing great potential in photocatalytic fields.^[^ ^56^ ^]^ Nevertheless, the performance of TSC viologens is still hindered by insufficient charge separation and transfer, and rapid charge carrier recombination did not achieve light‐induced electron transfer, which greatly limits their application in visible‐light‐driven photocatalysis, especially in visible‐light photocatalytic coupling reactions.

Simultaneously, donor–acceptor (D–A) interaction as a representative of the through‐bond conjugation (TBC), consisting of electron‐rich donor and electron‐deficient acceptor, has attracted considerable attention for their widespread applications in photocatalysis,^[^ ^57^ ^]^ solar cells,^[^ ^58^ ^]^ and organic photovoltaics.^[^ ^59^ ^]^ The difference in electron affinity potentials of the donor and acceptor leads to a large dipole moment of D–A materials, which facilitates charge transfer (CT) of the excited state. CT interaction is beneficial for the wide spectral response range and a redshift of optical absorption edge. In addition, the photogenerated electrons concentrated on the acceptor section could efficiently improve the exciton dissociation. It is demonstrated that D–A interaction is an effective strategy for accelerating carrier transport, inhibiting charge recombination, improving the exciton dissociation, narrowing band gap, and facilitating surface photocatalytic reactions, which plays a significant role in optimizing the intrinsic properties of organic photoelectric materials.^[^ ^60^ ^]^ Recently, a series of D–A naphthalene‐viologen‐based cyclophanes were synthesized and characterized by J. Fraser Stoddart et al. Photoinduced intramolecular and intermolecular CT was revealed by them, which augurs well for their use in the development of multi‐responsive organic electronic and optoelectronic materials.^[^ ^61^ ^]^ A new naphthalene‐based D–A COF was reported by Zeming Wang and coworkers to regulate the electron distribution for boosting photocatalysis.^[^ ^62^ ^]^ Inspired by the above studies, TBC and TSC are integrated into hybrid conjugation viologens for a synergistic effect, which could modulate the optoelectronic properties of viologens by stabilizing the free radical, broadening light absorption and enhancing good redox property to further narrow energy, enhance visible light absorption, and stabilize free radical of viologens. On the one hand, D–A conjugation could effectively regulate the electron distribution of viologens, accelerate CT along the molecular skeleton, and improve the efficiency of charge carrier separation, which is conducive to improving visible‐light‐driven photocatalysis. On the other hand, the electron delocalization of TSC could be through the space of the molecule, which could accelerate CT through intermolecular and intramolecular space.

**Scheme 1:**
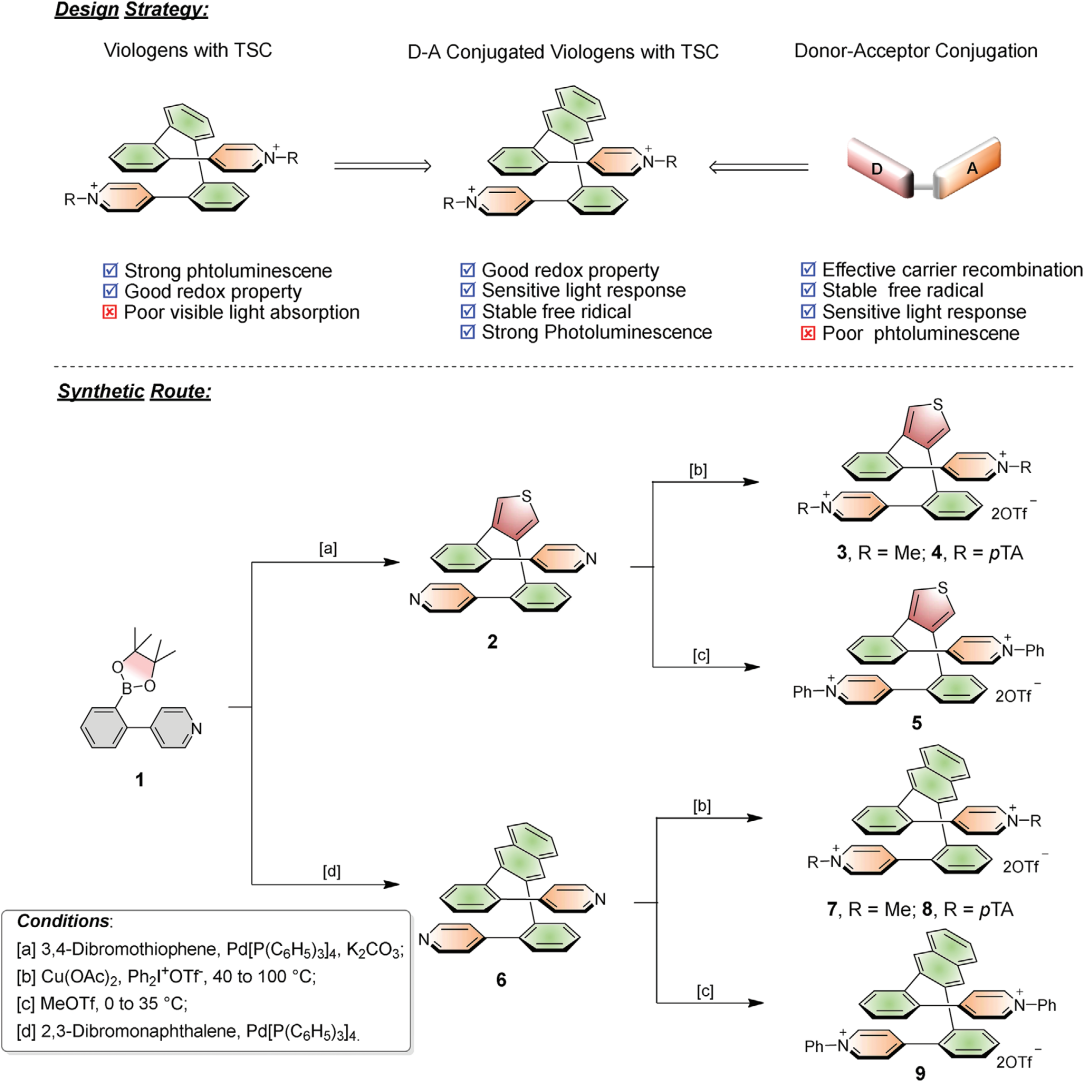
Design strategy and synthetic route to the TSC viologens.

Based on the considerations, D–A conjugation between naphthyl/thienyl and pyridine, through‐space conjugation with intermolecular and intramolecular were integrated to synthesize a series of novel D–A conjugated viologens with TSC units (diphenylnaphthalene or 3,4‐dioxythiophene) (**Scheme** **1**). Benefiting from the synergistic effect of D–A interaction and TSC, TSC viologens with D–A interaction exhibited a narrowed band gap, high visible optical absorption, excellent fluorescence, and redox properties. The solvent effect of fluorescence spectra revealed the photoinduced twisted intramolecular charge transfer (TICT), and the UV/vis spectra and DFT calculation identified the visible optical absorption and the narrower band. Femtosecond transient absorption (fs‐TA) experiments indicated that D–A conjugation and TSC can accelerate intramolecular charge transfer, prolong the charge separation state, and stabilize free radicals. Consequently, TSC viologens with D–A interaction possessing remarkable photoelectric performance exhibit excellent performance in photocatalytic oxidative coupling reaction and visible‐light‐driven hydrogen.

## 2 Results and Discussion

### 2.1 Synthesis and Structural Characterization

First, the precursor of TSC with D–A conjugation (**2**, **6**) as a white powder was synthesized via Suzuki‐Miyaura at 95 °C for 16h. Next, **3** and **7** with OTf^−^ as a white powder with 79% and 75% yield were obtained using the excess MeOTf for anion exchange by **2** and **6**, respectively. To obtain **4** and **8** with OTf^−^, **2** and **6** were reacted with 4‐bromomethylbenzoic acid and anion exchange as a white powder with 45% and 50% yield. **5**/**9** were obtained as yellow powder with 50%/47% yield with a catalyst of Cu(CH_3_COO)_2_, diphenyl iodonium triflate and **2**/**6**. ^1^H, ^13^C, ^19^F, correlation spectroscopy (COSY) nuclear magnetic resonance (NMR), and high‐resolution mass spectrum (HRMS) of the target molecules **2**–**9** are shown in supporting information, which is critical evidence for the molecular formula of **2–9**. The single crystals of **2**, **3**, **6**, **8**, and **9** were obtained by solvent evaporation (MeCN or dichloromethane) for X‐ray diffraction experiments (Tables S1–S5, Supporting Information). Besides, the abbreviation name of the compound is summarized in Supporting Information.

**Figure 1:**
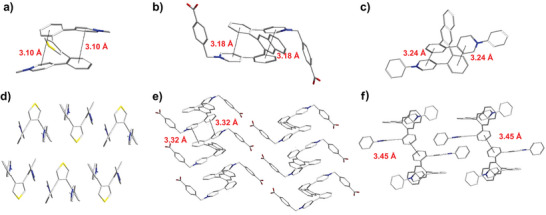
X‐ray single‐crystal structure of single molecular structure of a,d) **3**, b,e) **8**, c,f) **9**. The π–π interactions are depicted as black dashed lines. Color code: C, grey; N, blue; S, yellow and O, red. Counter ions, solvent molecules, and hydrogen atoms have been omitted for clarity.

As shown in Figure S1 (Supporting Information), the crystal structure directly confirms the π^…^π interactions of **2** with the distance of 3.24 Å, which is the typical distance for TSC interaction reported previously After anion exchange, there are still π^…^π interactions with a distance of 3.10 Å between molecules of compound **3** (**Figure** **1**a,d). From the crystal of **6**, the distance of intramolecular and intermolecular π^…^π interactions is 3.17 and 3.40 Å, respectively (Figure S2, Supporting Information). Similarly, there are intramolecular and intermolecular TSC in **8** and **9** from Figure 1. A single crystal structure of **8** indicates that the distance between the benzene ring and adjacent pyridine is 3.18 Å, while the distance of the molecule is 3.32 Å (Figure 1b,e; Figure S3, Supporting Information). There are multiple π^…^π interactions with the distance of 3.24 and 3.45 Å between the benzene ring and adjacent pyridine or benzene rings on adjacent molecules (Figure 1c,f; Figure S4, Supporting Information). In conclusion, the intramolecular and intermolecular π^…^π interactions of through‐space conjugation molecules (TSCMs) (compounds **2** and **6**) and viologens with intramolecular and intermolecular TSC (compounds **3**, **8,** and **9**) were proved directly by the single crystal structure, which could provide multiple pathways for electrons to transfer via intramolecular and intermolecular.

### 2.2 Photophysical Properties

**Figure 2:**
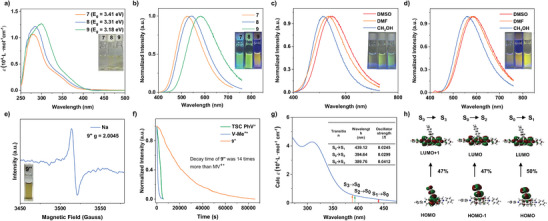
a) UV/vis spectra and b) fluorescence spectra of **7**, **8**, and **9** in DMF. The inset photographs of **7**, **8**, and **9** in DMF upon daylight and UV light (365 nm). d) Fluorescence spectra of **9** at room temperature in different solutions (DMSO, CH_3_OH, and DMF) (c = 2.0 × 10^−5^ m). The inset photographs of **9''** in different solutions (DMSO, CH_3_OH, and DMF). e) EPR spectrum of the radical species of **9''** by adding sodium at room temperature. EPR spectrum of **9** by adding zinc at room temperature. The inset photographs of **9′'** in DMF upon daylight. f) Optical memory of **9''**, TSC PhV**''** and MV^+•^ at 525 nm in the air. g) Imitating UV/vis absorption spectra of **9** in DMF. The inserted tables are the calculated oscillator strengths of transitions and orbital amplitude plots of **9**. h) Calculated dominant orbital energy levels and HOMO/LUMO orbital plots of **9**.

Infrared Spectroscopy experiments were used to characterize the different functional groups of the compounds. As shown in Figure S5 (Supporting Information), absorption peaks appear at 1630–1640 cm^−1^ and 1200–1350 cm^−1^ after anion exchange could be attributed to the S═O and C─F bond of OTf^−^ after anion exchange. For **4** and **8**, the strong absorption peak of 1700 cm^−1^ may be attributed to the C═O bond at carboxylic acid, and the wide absorption peak at 3500 cm^−1^ may be attributed to the O─H bond at carboxylic acid, which can determine the carboxylic acid of **4** and **8**. The above absorption peaks are slightly redshifted compared with traditional carboxylic acid groups, which could be attributed to the effect of hydrogen bonding and through‐space conjugation. To study optical absorption properties, UV/vis spectra of TSC viologens with D–A conjugation were collected. Different from the light absorption of TSCMs **2** (333 nm), TSC viologens with D–A conjugation of **3**, **4,** and **5** showing absorption in the visible light region were up to 376, 397, and 457 nm (Figures S6 and S7, Supporting Information). Remarkably, **5** in DMF exhibited a yellow color in DMF upon daylight (Figure S7, Supporting Information). The calculated energy gap was 3.10 eV (**5**), which was narrower than 4.03 eV (**2**), 3.49 eV (**3**) and 3.38 eV (**4**) (Figure S8, Supporting Information; **Figure** **2**a). Similarly, the maximum absorption of **6**, **7,** and **8** were 347, 400, and 418 nm, while their calculated energy gaps were 3.77, 3.41, and 3.31 eV (Figure 2a). The results wavelength and calculated energy gaps of **9** were 460 nm and 3.18 eV. Furthermore, the energy gap and maximum absorption of TSC viologens reported by our groups were 3.25 eV and 312 nm. Compared with TSC viologens,^[^ ^56^ ^]^ TSC viologens with D–A conjugation (TSC‐TV^2+^ and TSC‐NV^2+^) exhibited a narrower band gap and visible light absorption, which could be attributed to that ionization could effectively reduce the energy gap and higher conjugation degree could result in a significant redshift in light absorption. Benefiting from the ionization and higher conjugation degree of intramolecular/intermolecular TSC, the maximum absorption synergistic effect of TSC and D–A conjugation provides a basis for the subsequent visible‐light‐driven photocatalysis. Furthermore, fluorescence spectra of TSC viologens with D–A conjugation were obtained to study their structure and performance.

Upon UV irradiation (λ = 365 nm), the TSC viologens with D–A conjugation exhibit different colors in DMF, the fluorescence emission spectra of **3**, **4,** and **5** show a peak of 473, 499, and 533 nm, while the fluorescence emission peak of **7**, **8** and **9** was 524, 544 and 582 nm (Figure 2b; Figure S9, Supporting Information). Notably, compared with TSC‐TV^2+^, the fluorescence emission spectra of TSC‐NV^2+^ redshifted obviously. Compared with TSC‐TV^2+^, the reason for the redshifted of TSC‐NV^2+^ fluorescence emission spectra could be attributed to the higher degree of their conjugation. According to X‐ray diffraction experiment results, TSC‐NV^2+^ possessed intramolecular and intermolecular TSC. But TSC‐TV^2+^ only exhibited intramolecular TSC. When the degree of conjugation increases, the degree of electron delocalization also increases. This increased degree of delocalization causes electrons within molecules to move more easily within larger conjugated systems, resulting in higher HOMO (highest occupied molecular orbital) and lower LUMO (lowest unoccupied molecular orbital). This change reduces the band gap of the molecule. According to the Planck‐Einstein relation (E = hc/λ), a decreased energy leads to an increased wavelength.^[^ ^16^ ^]^ Besides, **7**, **8**, and **9** showed strong fluorescence with Φ_F_ values of 28.19%, 27.62%, and 14.83%, while **3**, **4,** and **5** exhibited weaker fluorescence with Φ_F_ values of 1.55%, 3.41%, and 2.84% (Figures S10–S12 and Table S7, Supporting Information). Additionally, the fluorescence lifetime of TSC‐NV^2+^ in DMF was much longer than TSC‐TV^2+^ as shown in Table S6 (Supporting Information). The fluorescence spectra of **5** and **9** at room temperature in different solutions were shown in Figure 2c,d to study the charge transfer of TSC viologens with D–A conjugation. As the polarity of the solvent increases, the color in different solutions upon UV irradiation (365 nm) changed and the fluorescence emission peaks of **5** and **9** exhibited a redshift, that could be attributed to TICT brought from the strong D–A interactions between the electron‐rich donor (naphthyl/thienyl) and electron‐deficient acceptor (pyridine).^[^ ^63^, ^64^, ^65^ ^]^

### 2.3 Transient Absorption Spectroscopy

**Figure 3:**
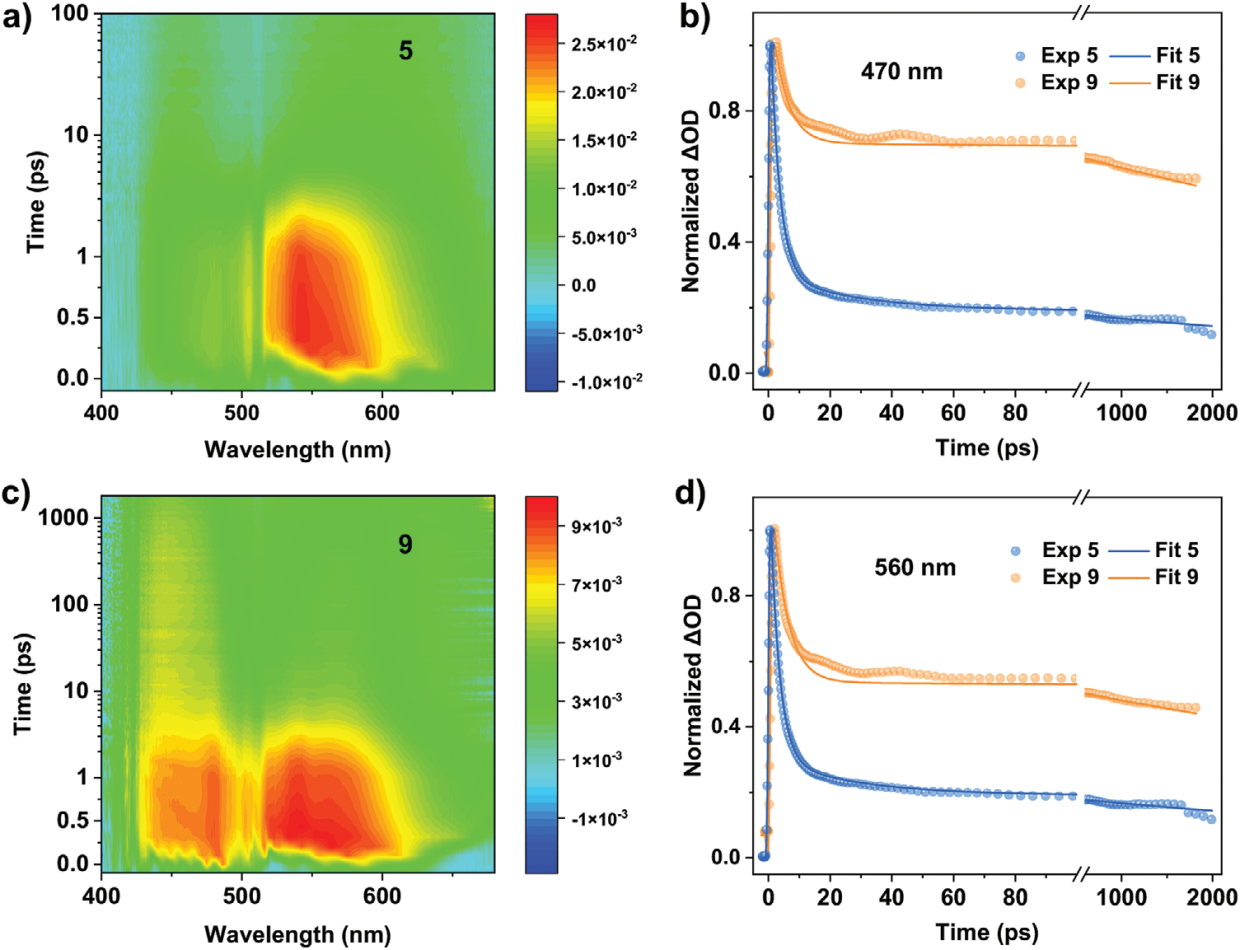
Transient absorption spectra. a,c) Contour map of **5** and **9** in DMF. b,d), Decay curves in transient absorption of **5** and **9** probed at 470 and 560 nm.

Additionally, the fs‐TA measurements were used to investigate the photoexcitation and CT processes of TSC viologens with D–A conjugation. Under the excitation at 390 nm, the change of absorbance signals was observed in the range of 450–650 nm. The characteristic peaks at 550 nm (in 1 ps) of **5** or **9** instant increase, indicating the formation of excited state 5* or 9*, which coincided with the electrochemical spectra (**Figure** **3**a,c; Figures S38c and S39c, Supporting Information). CT processes of **5** and **9** could be investigated by multiexponential fitting of kinetic traces at 470 and 560 nm (Figure 3b,d; Figures S13–S15, Supporting Information). Decay curves in transient absorption could be split into three states through the multiexponential fitting, which corresponds to the first state of excited states formation, the second state of charge separation states formation, and the charge recombination process. For TSC viologens with D–A conjugation of **9**, the excited states formed in the time scale of ≈0.32 ps and decayed in a long time (≈8000 ps), which indicates a long‐live charge recombination process. Besides, the excited states of **5** formed in the time scale of ≈2.93 ps under excitation light and delayed in ≈667 ps. Notably, compared with **5**, the longer charge recombination process of **9** could be attributed to stronger D–A interactions. Similarly, the characteristic peaks at 600 nm (in 1 ps) of **4** or **8** instant increase are related to the formation of excited state 4* or 8*, which is consistent with their chemical spectra (Figures S13, S39b, and S40b, Supporting Information). Moreover, multiexponential fitting of kinetic races indicated that the excited states of **4** and **8** formed in the time scale of ≈2.91 and 0.28 ps under excitation light, and delayed in ≈4280 and 9000 ps, respectively (Figure 3a,c; Figure S16–S19, Supporting Information). Compared with TSC‐TV^2+^, the excited states of TSC‐NV^2+^ formed faster and delayed slower, which could be attributed to the synergistic effect of D–A interaction and TSC supplied a faster charge separation state, accelerated generation of electron transfer processes and prolonged charge recombination process, which is very favorable for their application in photocatalysis.

### 2.4 Electrochemical Properties

Cyclic voltammetry (CV) and differential pulse voltammetry (DPV) tests were conducted to investigate the electrochemical characteristics. **3** and **7** showed two redox peaks, that were consistent with the theoretical two redox states, which was further proved by DPV (Figures S20 and S23, Supporting Information). Nevertheless, TSC viologens with 4‐(bromomethyl) benzoic acid (**4** and **8**) and phenyl (**5** and **9**) displayed only one redox peak that was inconsistent with the theoretical two redox states, and DPV graphs were asymmetric (Figures S21, S22, S24, and S25, Supporting Information). This discrepancy could be attributed to the overlapping of two redox adjacent peaks owing to the larger electron delocalization area. Furthermore, electron‐transfer constants (*k*_ET_), HOMO/LUMO orbital energy, and energy gap were calculated from the absorption spectra and onset reduction potential (Figure S26, Supporting Information). The calculated energy gap of **9** was 3.18 eV, which was narrower than that of the reported TSC viologens (Table S8, Supporting Information). Besides, the *k*_ET_ of TCS viologens with D–A conjugations was 9.54 × 10^−4^ cm s^−1^, which was higher than that of the reported TSC viologens, indicating D–A conjugation was beneficial to improve CT (Table S9, Supporting Information).^[^ ^66^ ^]^

### 2.5 Density Functional Theory (DFT) Calculations

To further confirm the above experimental results, DFT calculations of orbital energy levels and UV/vis absorption were performed for TSC viologens with D–A conjugation. The calculated results of UV/vis absorption spectra were in good agreement with the experimental results, which show a narrower band gap between LUMO and HOMO energy levels as the conjugation degree enhanced, which was consistent with the previously reported study (Figures S27–S32, Supporting Information). Specially, the LUMO and HOMO energy level of **9** were −3.24 and −6.41 eV. The energy gap between the LUMO and HOMO orbit was 3.17 eV, which was consistent with the calculated orbital energy level by CV measurement and the narrowest among TSC viologens with D–A conjugation (Figure 2a; Tables S10–S15, Supporting Information). The simulated UV spectrum shows that the main absorption peak of **9** is located at 309 nm, and the broad absorption tail extends to 460 nm, which was consistent with the UV/vis spectrum of **9**. The peaks at 439.12, 394.64, and 389.76 nm belong to the S_0_ → S_1_ transition, S_0_ → S_2_ transition, and S_0_ → S_3_, transitions exhibited obvious TSC characteristics (Figure 2g,h). The calculated orbitals of the compounds are shown in Figures S33 and S34 (Supporting Information). Besides, as shown in Figures S35–S38 (Supporting Information) TSC viologens with D–A conjugation and their radical species consist of red electron‐rich donor (naphthyl and thienyl) and blue electron‐deficient acceptor (pyridine). The great difference in electron affinity potentials of the donor and acceptor led to a large dipole moment of D–A materials. The electrostatic potential around naphthyl was higher than thienyl, indicating the stronger D–A conjugation interactions of TSC‐NV^2+^ (Figures S35–S38, Supporting Information).

### 2.6 Chemical Reduction Reaction

For evaluating the influence of TSC and D–A conjugation on radicals, the redox process in the chemical reduction reaction was investigated. EPR spectra were measured after adding zinc powder to the viologens DMF solution. The results showed that EPR signals were weak without obvious color change, which indicated zinc powder is insufficient to reduce viologens to free radical states. The DMF solutions of **3**, **4**, **5**, **7**, **8,** and **9** could be reduced to radical by Na with obvious color change. White compounds of **3** and **7** could be reduced by Na into yellow free radicals, which matched well with the spectroelectrochemistry and CV experiments (Figure 2e; Figures S39 and S40, Supporting Information). Na could reduce compounds **4** and **8** into blue radicals, and their UV/vis spectra exhibited an absorption peak of ≈600 nm (Figures S39 and S40, Supporting Information). Similarly, **5** and **9** could be reduced by Na into yellow radical with absorption peaks of UV/vis spectra occurring ≈450 and 600 nm (Figures S39 and S40, Supporting Information). The free radicals of TSC viologens were identified by electron paramagnetic resonance (EPR) analysis (Figure 2e; Figures S41 and S42, Supporting Information). Remarkably, benefiting from the large area of electron delocalization and D–A conjugation, the free radical **9''** was stabilized effectively, and the decay time of **9''** was ≈14 times than that of MV^+•^ and reported TSC Ph**''** in the air (Figure 2f).^[^ ^67^ ^]^

### 2.7 Photocatalytic Oxidative Coupling Reaction

**Figure 4:**
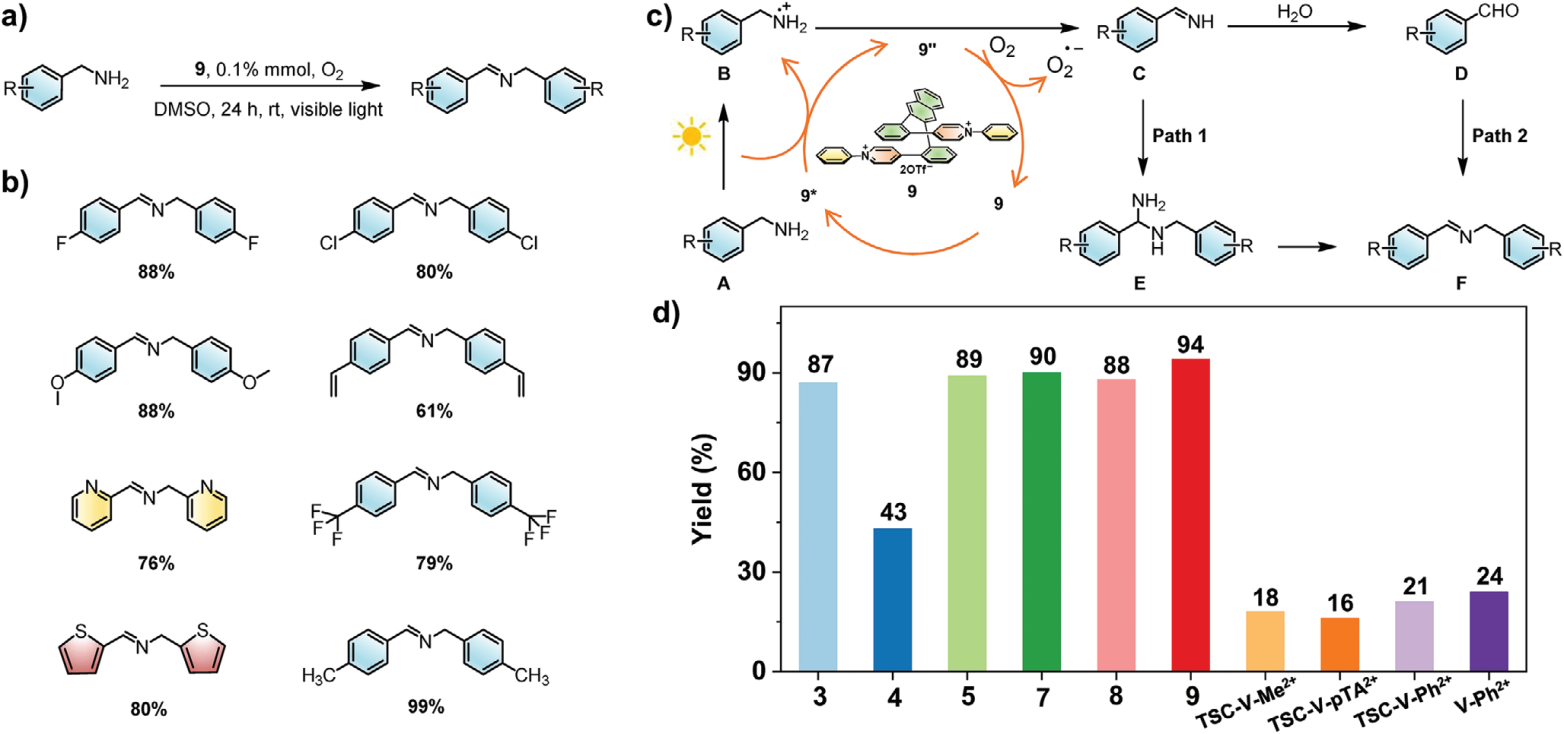
a) Photocatalytic oxidative coupling into amines by catalyst **9**. b) The yield of photocatalytic oxidative coupling of primary amines by catalyst **9**. c) Proposed mechanism of photocatalytic oxidative coupling into amine. d) The yield of photocatalytic oxidative coupling into amines by different viologens catalysts.

Based on the remarkable photoelectronic properties of TSC viologens with D–A conjugation, we elucidated their photocatalytic performance as a catalyst for the aerobic oxidation of amines to imines.^[^ ^60^, ^68^, ^69^ ^]^ As shown in **Figure** **4**a and Table S16 (Supporting Information), benzylamine was chosen to investigate the photocatalytic activity. Based on the visible light absorption of TSC viologens with D–A conjugation, visible light was chosen as the light source. The common organic solvents and atmosphere of the reaction were screened, and the results indicated that a higher yield could be achieved under O_2_ with the solvent DMSO. Besides, no amine was detected in dark conditions or without catalyst **9**, illustrating that light and catalyst are indispensable in photocatalytic oxidative coupling into amines. Subsequently, the yield of photocatalytic oxidative coupling into amines by TSC viologens with D–A conjugation was studied. As shown in Figure 4d, the yield with the catalyst of **9** is up to 94%, which is much higher than TSC viologens and general viologens, indicating the advantage of the synergistic effect from D–A conjugation and TSC (Table S16, Supporting Information). Furthermore, several amines with various substituents for the photocatalyzed oxidation were investigated, and the yield with the catalyst of **9** was still high, indicating good substrate compatibility (Figure 4b; Table S17, Supporting Information). Subsequently, a mechanism for the photocatalyzed aerobic oxidation of benzylamine by the TSC viologens with D–A conjugation is proposed based on the above experimental results and previously reported results for analyzing the reasons for the excellent performance.^[^ ^70^, ^71^ ^]^ Under visible light, the catalyst of **9** was form **9''** and anion radical B. At the same time, O_2_ was reduced to superoxide radicals

O_2_ **^·−^** by obtaining an electron. **B** lost a proton and H^+^ through O_2_^·−^ to obtain **C**. Afterward**, C** transformed into the final product through two possible paths 1 and 2. In path 1, C collided with aniline to form the intermediate E, which lost NH_3_ to obtain the final product F. In path 2, under a trace of water, C conversed to intermediate D, which combined with aniline to achieve the target product. D–A conjugated viologens with TSC (**9**) could reach the yield of 94% as a catalyst in the oxidative coupling reaction under visible light, which could be attributed to the broadened light absorption into visible light, prolonged charge recombination process, and stable free radical. Visible light absorption proved by UV/vis spectra is the prerequisite for free radicals formation of excited by visible light, while **9** showed a broad absorption into visible light. For TSC viologens with D–A conjugation of **9**, the excited states formed in the time scale of ≈0.32 ps and decayed in a long time (≈8000 ps), indicating a faster free radical formation and a longer‐live charge recombination process compared with other viologens, that could provide more efficient charge to capture electrons for next photocatalytic reactions (Figure 3c,d). Meanwhile, the excellent stability of free radical **9''** was proved by longer life in the air, which could be good to be captured by oxygen for catalyst circulation (Figure 2f).

### 2.8 Visible‐Light‐Driven Hydrogen Production

**Figure 5:**
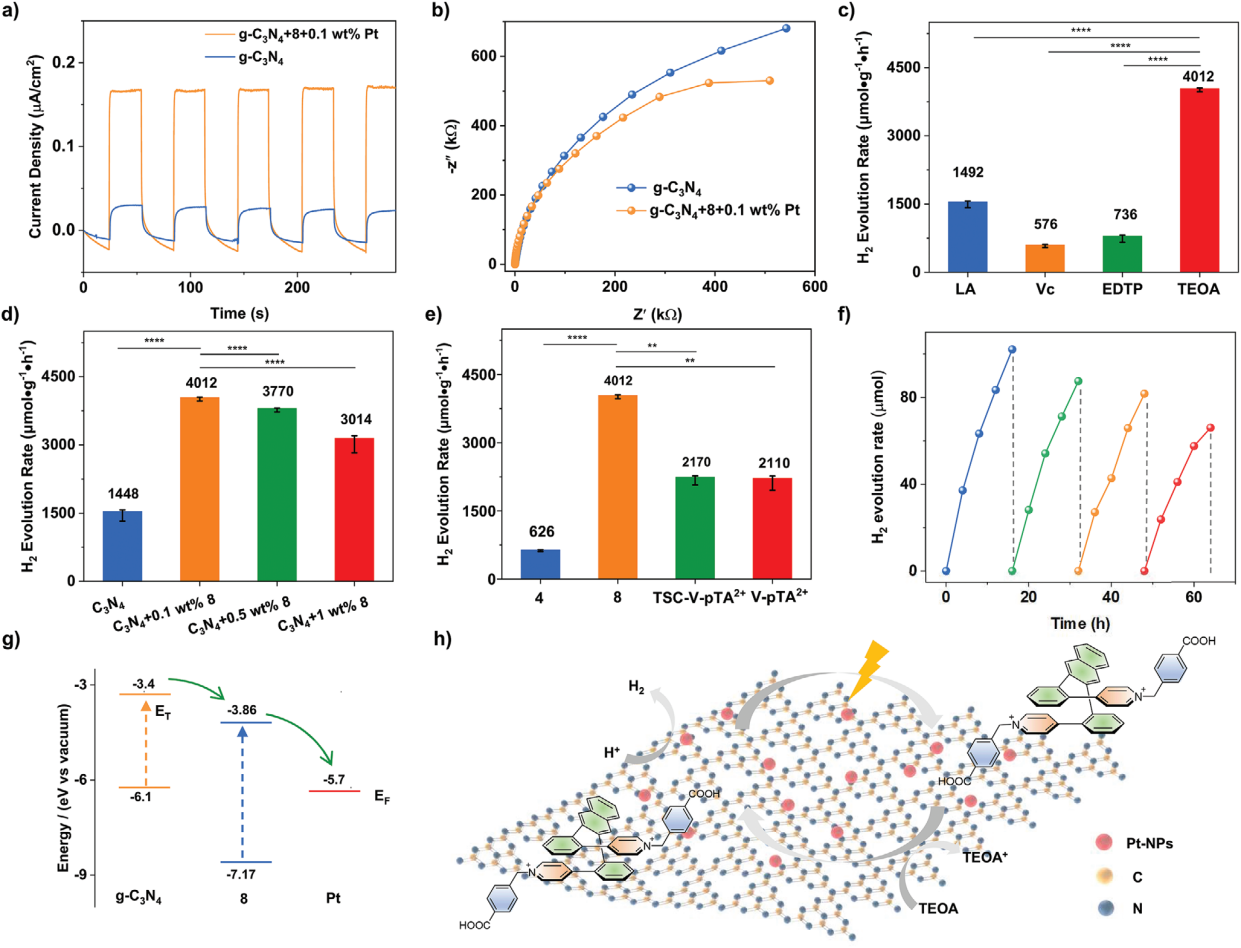
a) Transient photocurrent and b) EIS spectra of g‐C_3_N_4_ and g‐C_3_N_4_/0.1 wt.% **8** with 1 wt.% Pt cocatalyst. c) The screening of the sacrificial agent was to use catalyst g‐C_3_N_4_/0.1 wt.% **8**/Pt in a 10% aqueous solution of different sacrificial agents (mean values: 1492 for LA, 576 for Vc, 736 for EDTP, and 4012 for TEOA, error bar: 73.5 for LA, 33.9 for Vc, 79.2 for EDTP and 42.4 for TEOA). d) Different proportions of **8** with 1 wt.% Pt cocatalyst (mean values: 1448 for C_3_N_4_, 4012 for C_3_N_4_ + 0.1% **8**, 3770 for C_3_N_4_ + 0.5% **8** and 4012 for C_3_N_4_ + 1% **8**, error bar: 124.5 for C_3_N_4_, 42.4 for C_3_N_4_ + 0.1% **8**, 42.4 for C_3_N_4_ + 0.5% **8** and 189.5 for C_3_N_4_ + 1% **8**). e) **4**, **8**, TSC‐V‐pTA^2+^ and V‐pTA^2+^. f) Cyclic stability test of photocatalytic hydrogen production over g‐C_3_N_4_/0.1 wet % **8** with 1 wt.% Pt cocatalyst (mean values: 626 for **4**, 4012 for **8**, 2170 for TSC‐V‐pTA^2+^ and 2110 for V‐pTA^2+^, error bar: 19.8 for **4**, 42.4 for **8**, 99.0 for TSC‐V‐pTA^2+^ and 155.6 for V‐pTA^2+^). g) Energy level diagram of the vacuum level of the system with g‐C_3_N_4_/**8**/Pt. h) Schematic diagram of visible‐light‐driven hydrogen production with g‐C_3_N_4_/**8**. (The data were collected and are shown as the mean in c, d, and e, sample size (n) = 3, probability (P) value: ^****^ *p* <0.0001 and ^**^ *p* <0.01).

Graphitic carbon nitride (g‐C_3_N_4_),^[^ ^72^, ^73^, ^74^ ^]^ is one of the most classic semiconductors with a band gap of 2.7 eV and has attracted tremendous attention recently. Nevertheless, the high recombination rate of photogenerated electron‐hole pairs still restricts the further application of g‐C_3_N_4_. It has been demonstrated that introducing a redox medium to modify g‐C_3_N_4_ could effectively avoid the recombination of photogenerated electron‐hole pairs, thus improving light utilization and photoelectric conversion efficiency.^[^ ^75^, ^76^ ^]^ Based on the consideration, the composite of TSC viologens with D–A conjugation, g‐C_3_N_4_, and Pt nanoparticles was synthesized for a visible‐light‐driven hydrogen production system (Figure S43, Supporting Information). In this system, D–A TSC viologens with an efficient charge separation process were applied as electron mediators and photosensitizers, while g‐C_3_N_4_ and Pt were as a photosensitizer and catalysts. To illustrate that TSC viologens and Pt nanoparticles were dispersed in g‐C_3_N_4_, many characterization methods such as diffuse reflection UV/vis absorption spectrum, X‐ray diffraction pattern, and time‐resolved photoluminescence were applied (Figures S44–S50, Supporting Information). C, N, Pt, F, and S elements uniformly dispersed on 2D layered C_3_N_4_ as the TEM images shown, which confirmed **8** and Pt nanoparticles were doped in g‐C_3_N_4_ (Figure S44, Supporting Information). Besides, the UV/vis absorption spectrum of the composite showed a slight blue shift compared with g‐C_3_N_4_, indicating that a small amount of **8** existed in g‐C_3_N_4_ (Figure S45, Supporting Information). Two peaks at 13° and 27° observed in XRD patterns of the composite and g‐C_3_N_4_ corresponded to the (100) plane and (002) plane of g‐C_3_N_4_, and the ratio of two peaks changed greatly, indicating crystallinity would be reduced by **8** and Pt nanoparticles (Figure S46, Supporting Information). In addition, compared with g‐C_3_N_4_, the steady‐state photoluminescence spectrum intensity of the composite was weaker than g‐C_3_N_4_, which was attributed to the charge transfer interaction between **8** and g‐C_3_N_4_ (Figure S47, Supporting Information). X‐ray photoelectron spectroscopy (XPS) analysis was applied to further investigate the chemical composition and the surface bonding interactions of the composite materials. XPS spectra of g‐C_3_N_4_, **8,** and the composite were shown in Figures S48–S50 (Supporting Information). XPS spectra indicated that g‐C_3_N_4_ consists of C, N, and O elements, while the signals of C, N, O, S, and F could be detected on the surface of **8**. The XPS spectra indicated that the composite integrated the chemical composition and the surface bonding interactions of g‐C_3_N_4_ and **8**. Additionally, the peak of C 1s at 284.7 eV was attributed to C─C. The relative intensity of peak ≈284.7 eV was reduced apparently after hybrid g‐C_3_N_4_ and **8**, which indicates the surface interaction such as hydrogen bonds between rich amino groups on g‐C_3_N_4_ and carboxyl of **8** as shown in Figure S50 (Supporting Information).^[^ ^75^ ^]^ Photoelectrochemical (PEC) measurements were used to study the separation and transfer efficiencies of photo‐generated carriers. It can be seen that the photocurrent intensity of the composite was higher than g‐C_3_N_4_, which indicated the composite generated much more photoinduced carrier under visible light (**Figure** **5**a). Electrochemical impedance spectroscopy (EIS) was applied to explore the insightful photophysical mechanism. The lowest resistance is reflected in the smallest diameter of the semicircle in the Nyquist plot.^[^ ^77^ ^]^ As shown in Figure 5b, the electrochemical impedance spectra (EIS) radius of the composite is smaller than g‐C_3_N_4_, that indicated the interfacial charge transfer rate of the composite is faster than g‐C_3_N_4_ (Figure 5a,b).

First, the pure g‐C_3_N_4_ system could not produce hydrogen, only the composite system consisting of g‐C_3_N_4_ and Pt could produce hydrogen. It proved that g‐C_3_N_4_ and Pt were respectively as a photosensitizer and catalyst in the composite system. Several control experiments including sacrificial agent species and proportions of **8** were controlled to optimize the reaction conditions. First, triethyl orthoacetate (TEOA) was chosen as a sacrificial agent owing to faster hydrogen production rate than Lactic acid (LA), Ascorbic acid (Vc), and *N, N, N’, N’*‐ethylenediamine tetrakis (methylene‐phosphonic acid) (EDTP) (Figure 5c). As shown in Figure 5d, introducing **8** into the composite of g‐C_3_N_4_ and Pt nanoparticles could significantly accelerate the hydrogen production rate. Especially, the hydrogen production rate of composite with C_3_N_4_, Pt nanoparticles, and 0.1wt.% **8** could reach up to 4012 µmol h^−1^g^−1^ was more competitive than other catalysis systems consisting of C_3_N_4_, but then decreased with the amount of **8** increasing (Figure 5d). Besides, different conjugations (TSC‐V‐pTA^2+^, V‐pTA^2+^) with carboxyl were introduced into g‐C_3_N_4_ to investigate the influence of the redox medium on the hydrogen production performance. The hydrogen production rate of C_3_N_4_/0.1 wt.% **8**/1wt.% Pt composite was higher than other conjugation viologens (Figure 5e). Furthermore, the high hydrogen production rate of 4012 µmol h^−1^g^−1^ is much higher than other composites consisting of C_3_N_4_. Compared with similar catalyst systems consisting of C_3_N_4_, C_3_N_4_/0.1 wt.% **8**/1 wt.% Pt exhibited a higher hydrogen production rate. Besides, this system also had great advantages compared with other systems of covalent organic framework (COF) consisting of viologens (**Table** **1**).

**Table 1:** Hydrogen generation activities of some organic photocatalytic systems.

Photocatalytic systems	Catalysis	Hydrogen generation rate	Conditions	References
g‐PAN/ g‐C_3_N_4_	1.5 wt.% Pt	370 µmol h^−1^g^−1^	10 vol% TEOA aqueous λ > 400 nm	[78]
CBV^2+^/ g‐C_3_N_4_	1 wt.% Pt	831 µmol h^−1^g^−1^	10 vol% TEOA aqueous λ > 400 nm	[75]
C‐PAN/ g‐C_3_N_4_	3 wt.% Pt	1775 µmol h^−1^g^−1^	10 vol% TEOA aqueous λ > 400 nm	[76]
PDA/ g‐C_3_N_4_	3 wt.% Pt	1380 µmol h^−1^g^−1^	10 vol% TEOA aqueous λ > 400 nm	[79]
NHPI/ g‐C_3_N_4_	1 wt.% Pt	1145 µmol h^−1^g^−1^	10 vol% TEOA aqueous λ > 400 nm	[80]
DHPEI/ g‐C_3_N_4_	1 wt.% Pt	1345 µmol h^−1^g^−1^	10 vol% TEOA aqueous λ > 400 nm	[81]
VOPc/ g‐C_3_N_4_	1 wt.% Pt	1310.40 µmol h^−1^g^−1^	10 vol% TEOA aqueous λ > 400 nm	[82]
pTA‐TPV^2+^/ g‐C_3_N_4_	1 wt.% Pt	2404 µmol h^−1^g^−1^	10 vol% TEOA aqueous λ > 400 nm	[83]
[Pt(bpy)(dmMV^2+^)_2_] (PF_6_) _4_•H_2_O	‐	0.59 µL min^−1^	acetate buffer solution (0.1 M; pH 5) 800 nm>λ > 400 nm	[84]
TPCBPB‐COF	‐	1530 µmol h^−1^g^−1^	TEOA λ > 400 nm	[85]
Tp‐BPy‐COF	Pt	346 mmol h^−1^	H_2_A λ > 420 nm	[77]
TPCBP‐E‐TiO_2_	Pt	1013 µmol h^−1^g^−1^	TEOA λ > 400 nm	[86]
COF‐MB‐TPCBP	‐	4000 µmol h^−1^g^−1^	TEOA λ > 400 nm	[87]
CbV^2+^‐based FeSP	PVP‐Pt	870 µmol h^−1^g^−1^	EDTA λ > 400 nm	[88]
**8**/g‐C_3_N_4_	1 wt.% Pt	4012 µmol h^−1^g^−1^ (2.25 µL min^−1^)	10 vol% TEOA aqueous λ > 400 nm	***This work***

Subsequently, to explore the reasons for superior performance, photoelectrochemical measurements were applied to investigate the separation and transfer efficiencies of photo‐generated carriers. The result can be speculated from the following aspects. Electron‐hole pairs generated by C_3_N_4_ under visible light will be inclined to recombine with the absence of **8**. Nevertheless, introducing **8** into C_3_N_4_ will restrain the recombination of electron‐hole pairs. The energy level diagram of the vacuum level of the system with g‐C_3_N_4_/**8**/Pt was illustrated in Figure 5g, the conduction band and valence band relative to the absolute vacuum of g‐C_3_N_4_ (−3.4 and −6.1 eV) are both higher than that of **8** (−3.86 and −7.17 eV), which could promote the photogenerated electron on g‐C_3_N_4_ transform to **8** and formation radical of **8''**. Subsequently, the photogenerated electron was captured by Pt NPs with the lower Fermi level (−5.7 eV) to reduce H^+^ into H_2_, while the generated hole will be caught by TEOA to restrain the backward reaction. Consequently, modifying g‐C_3_N_4_ with **8** and Pt nanoparticles could prevent photogenerated electron‐hole pairs from recombining and enable photogenerated electron transfer to Pt nanoparticles to accomplish the hydrogen production process, to enhance the hydrogen production performance of this system (Figure 5h). Notably, the cyclic stability of hydrogen production test was conducted to evaluate the durability, the H_2_ production rate of g‐C_3_N_4_/0.1 wt.% **8**/1wt.% Pt can retain over half of the initial performance indicating its excellent durability of H_2_ production (Figure 5f). Briefly, D–A conjugated viologens with TSC (**8**) could improve.

g‐C_3_N_4_ performance in hydrogen production under visible light, and achieve the H_2_ generation rate of 4102 µmol h^−1^g^−1^ as redox species hybridized and photosensitizer into g‐C_3_N_4_. Spectral energy level matching between them promotes photogenerated electrons on g‐C_3_N_4_ transforming to D–A conjugated viologens with TSC, which will prevent electron‐hole pairs generated by g‐C_3_N_4_ from recombining under visible light to reach a high generation rate.

## 3 Conclusion

In conclusion, a series of TSC viologens with D–A conjugation and excellent photoelectric properties were designed and synthesized via Suzuki‐Miyaura coupling, followed by *N*‐alkylation and *N*‐arylation. Their chemical structure and optoelectronic properties were confirmed using X‐ray diffraction, UV/vis spectroscopy, CV, DPV, EPR, PL, and fs‐TA experiments. Intramolecular and intermolecular π^…^π interactions were identified by X‐ray diffraction experiments. These findings indicate that TBC and TSC are integrated into a hybrid conjugation viologen system, offering multiple channels for CT, which enhances visible light absorption, improves redox properties, enhances ICT, prolongs the charge separation state, and stabilizes the radical. Given these excellent photoelectric properties, TSC viologens with D–A conjugation show significant potential in visible‐light‐driven photocatalytic applications. As a catalyst in oxidative coupling reactions under visible light, they achieve a high yield of 94% and exhibit good substrate compatibility. When used as redox species hybridized into g‐C₃N₄ for hydrogen production under visible light, the H₂ generation rate reaches up to 4012 µmol h^−1^g^−1^. This work not only broadens the application range of viologens but also provides a novel approach to integrating TSC viologens and TBC into a hybrid conjugation system to achieve a synergistic effect.

## 4 Experimental Section

### Synthesis

**1** (464.9 mg, 1.65 mmol), 1,2‐Diiodobenzene (100 mg, 0.41 mmol), Pd(PPh_3_)_4_ (48 mg, 0.04) and K_2_CO_3_ (229 mg, 1.65 mmol) was added to 50 mL two‐necked round bottom flask. The flask was evacuated under vacuum and flushed with dry nitrogen three times and then a 25 mL mixture of methylbenzene, ethyl alcohol, and deionized water (3/1/1, v/v/v) was added. The reaction mixture was heated and refluxed at 95 °C for 16 h to white product **2**.**2** (98 mg, 0.25 mmol) was dissolved in dichloromethane (5 mL) and cooled the solution to 0 °C, followed by methyl triflate (0.11 mL, 1 mmol) was added. The reaction mixture was stirred at 0 °C for 5 min, then warmed up to 35 °C and stirred for 12 h to obtain white product **3**. A mixture of **2** (98 mg, 0.25 mmol) and benzyl bromide (128 mg, 0.75 mmol) in anhydrous MeCN (20 mL) was stirred at 60 °C for 72 h to obtain a white product. The product was dissolved in dichloromethane (5 mL) and cooled the solution to 0 °C, followed by methyl triflate (0.11 mL, 1 mmol) was added. The reaction mixture was stirred at 0 °C for 5 min, then it was allowed to warm to 35 °C and stirred for 12 h to obtain white product **4**.**2** (98 mg, 0.25 mmol), diphenyl iodonium triflate (322.6 mg, 0.75 mmol), and anhydrous Cu(OAc)_2_ (3 mg, 0.017 mmol) in degassed DMF (4 mL) was stirred at 100 °C for 12 h under nitrogen atmosphere to obtain white product **5**.**1** (393.3 mg, 1.40 mmol), 1,2‐Diiodobenzene (100 mg, 0.35 mmol), Pd(PPh_3_)_4_ (40 mg, 0.04) and K_2_CO_3_ (193 mg, 1.40 mmol) was added to 50 mL two‐necked round bottom flask. The flask was evacuated under vacuum and flushed with dry nitrogen three times and then a 25 mL mixture of methylbenzene, ethyl alcohol, and deionized water (3/1/1, v/v/v) was added. The reaction mixture was heated and refluxed at 95 °C for 16 h to obtain a white product **6**.**6** (109 mg, 0.25 mmol) was dissolved in dichloromethane (5 mL) and cooled the solution to 0 °C, followed by methyl triflate (0.11 mL, 1 mmol) was added. The reaction mixture was stirred at 0 °C for 5 min, then warmed up to 35 °C and stirred for 12 h to obtain white product **7**. A mixture of **6** (109 mg, 0.25 mmol) and benzyl bromide (128 mg, 0.75 mmol) in anhydrous MeCN (20 mL) was stirred at 60 °C for 72 h under a nitrogen atmosphere. Then the mixture was cooled to room temperature, and the precipitate was collected and washed with an excess amount of acetonitrile (3 × 5 mL) by centrifuge to obtain a white product. The product was dissolved in dichloromethane (5 mL) and cooled the solution to 0 °C, followed by methyl triflate (0.11 mL, 1 mmol) was added. The reaction mixture was stirred at 0 °C for 5 min, then it was allowed to warm to 35 °C and stirred for 12 h to obtain white product **8**.

### Statistical Analysis

The date Crystal data and structure refinement, FT‐IR spectra, UV/vis absorption spectra, Lifetime decay profiles of phosphorescence bands, Transient absorption spectra, cyclic voltammogram, and XPS spectra were original. The Fluorescence spectra and EPR spectra were normalized. The Software of Origin was used to analyze and process all data. In the section on Photocatalytic oxidative coupling of amines into imines, three parallel experiments were carried out under the same condition at the same time to ensure the accuracy of the experiment, then the middle yield value was to make a contrast when three data had small errors. In the section on Hydrogen generation under xenon lamp, three parallel experiments were carried out under the same condition at the same time to ensure the accuracy of the experiment, the mean values (mean + SD) were chosen among three data small errors. Diamond and Mercury were used to analyze the Single‐crystal X‐ray structure. The photocatalytic hydrogen production rates of the sacrificial agent, different proportions of **8,** and different compounds were investigated. Specifically, the data were collected and were shown as the mean, sample size (n) = 3, mean values: 1492 for LA, 576 for Vc, 736 for EDTP, and 4012 for TEOA, error bar: 73.5 for LA, 33.9 for Vc, 79.2 for EDTP and 42.4 for TEOA, probability (P) value: ****p <0.0001). Mean values: 1448 for C_3_N_4_, 4012 for C_3_N_4_ + 0.1% **8**, 3770 for C_3_N_4_ + 0.5% **8** and 4012 for C_3_N_4_ + 1% **8**, error bar: 124.5 for C_3_N_4_, 42.4 for C_3_N_4_ + 0.1% **8**, 42.4 for C_3_N_4_ + 0.5% **8** and 189.5 for C_3_N_4_ + 1% **8**, probability (P) value: ^****^ *p* <0.0001; mean values: 626 for **4**, 4012 for **8**, 2170 for TSC‐V‐pTA^2+^ and 2110 for V‐pTA^2+^, error bar: 19.8 for **4**, 42.4 for **8**, 99.0 for TSC‐V‐pTA^2+^ and 155.6 for V‐pTA^2+^, probability (P) value: ^**^ *p* <0.01). The data of Femtosecond transient absorption measurements were analyzed by the Software of TASA_V2.5 and CarpetView. DFT calculation and Evaluation of HOMO and LUMO energy were conducted by GaussView6.0 and Xi'an Jiaotong University's high‐performance computing platform. Electrostatic potential surfaces and Computed UV/vis spectra were calculated by the Software GaussView 6.0.

## Conflict of Interest

The authors declare no conflict of interest.

## Supporting information



Supporting Information

Supporting cif

## Data Availability

The data that support the findings of this study are available from the corresponding author upon reasonable request.
