# Supplementary material for: Donor–Acceptor Viologens with Through‐Space Conjugation for Enhanced Visible‐Light‐Driven Photocatalysis
Source: Adv Sci (Weinh). 2024 Nov 21;12(2):2409925. doi: 10.1002/advs.202409925 (PMC11727243; doi:10.1002/advs.202409925)
Supplement: Supplementary file 1 — Supporting Information [file ADVS-12-2409925-s001.pdf]

ADVANCED SCIENCE
Open Access

# Supporting Information

for *Adv. Sci.*, DOI 10.1002/advs.202409925

Donor–Acceptor Viologens with Through-Space Conjugation for Enhanced Visible-Light-Driven Photocatalysis

*Yi Qiao, Liang Xu, Xiaoyang Liu, Yujing Gao, Naiyao Li, Yawen Li*, Tianle Cao, Ni Yan, Zishun Liu* and Gang He**

Supplementary Information

**Donor-Acceptor Viologens with Through-Space Conjugation for Enhanced Visible-Light-Driven Photocatalysis**

*Yi Qiao,[1] Liang Xu,[1] Xiaoyang Liu,[1] Yujing Gao,[1] Naiyao Li,[1] Yawen Li,[1,*] Tianle Cao,[2] Ni Yan,[2] Zishun Liu,[3,4*] Gang He[1,*]*

1. Yi Qiao, Liang Xu, Xiaoyang Liu, Yujing Gao, Naiyao Li, Yawen Li, Gang He
Frontier Institute of Science and Technology, Key Laboratory of Thermo-Fluid Science and Engineering of Ministry of Education, State Key Laboratory for Strength and Vibration of Mechanical Structures, Xi'an Jiaotong University, Xi'an, Shaanxi Province, 710054, P. R. China.
E-mail: liyawen@xjtu.edu.cn, ganghe@mail.xjtu.edu.cn

2. Tianle Cao, Ni Yan
School of Materials Science and Engineering, Chang'an University, 710064, Xi'an, China

3. Zishun Liu
International Center for Applied Mechanics; State Key Laboratory for Strength and Vibration of Mechanical Structures, Xi'an Jiaotong University, Xi'an, Shaanxi Province, 710054, P. R. China
E-mail: zishunliu@mail.xjtu.edu.cn

4. Zishun Liu
City University of Hong Kong (Dongguan), Dongguan, 523808, China

**Table of Contents**

1. Materials and instrumentation ..... 3

2. Synthetic procedures ..... 4

3. Single-crystal X-ray structure determination. ..... 13

4. Optical characterization data ..... 21

5. Femtosecond transient absorption measurements ..... 26

6. The cyclic voltammogram and Differential pulse voltammetry ..... 30

7. Evaluation of HOMO and LUMO energy ..... 33

8. Evaluation of electron-transfer constant $k_{ET}$ ..... 34

9. Computed UV/vis spectra ..... 36

10. DFT calculations ..... 45

11. Electrostatic potential surfaces ..... 47

12. UV/vis spectra of radical species in DMF ..... 49

13. EPR spectrum ..... 50

14. Photocatalytic oxidative coupling of amines into imines ..... 51

15. Hydrogen generation under xenon lamp ..... 54

16. $^{1}$H, $^{13}$C, $^{19}$F and 2D COSY NMR spectra and high resolution mass spectrum (HRMS) ..... 60

References ..... 84

## Experimental Procedures

### 1. Materials and instrumentation

**General Experimental Details.** Starting materials purchased from commercial suppliers were used without further purification. Melting points were recorded by using a XT-4 apparatus in open capillary tubes. NMR spectra were recorded on a spectrometer operating at 400 MHz for $^{1}H$ and 100 MHz for $^{13}C$ NMR spectra on a Bruker ascend spectrometer. Coupling constants were reported in Hz with multiplicities denoted as s (singlet), d (doublet), t (triplet), q (quartet) and m (multiplet). UV/vis measurements were performed using a Lambd 950 absorption spectrophotometer and fluorescence spectra were measured using a Hitachi F-7000 fluorescence spectrophotometer. The cyclic voltammetry (CV) and differential pulse voltammetry (DPV) in solution were measured using CHI660E B157216, with a polished gold electrode as the working electrode, a Pt-net as counter electrode, and an Ag wire as reference electrode, using ferrocene/ferrocenium ($Fc/Fc^{+}$) as internal standard. Electron paramagnetic resonance (EPR) was measured using a Bruker EMX PLUS6/1 instrument at room temperature in dry degassed N, N-dimethylformamide (DMF). High-resolution mass spectra (HRMS) were collected on a Bruker maxis UHR-TOF mass spectrometer in an ESI positive mode. The phosphorescence quantum efficiency, time-resolved phosphorescence spectra and lifetime were obtained using Edinburgh FLSP980 fluorescence spectrophotometer equipped with a xenon lamp (Xe900), a picosecond pulsed laser (EPL-375), a microsecond flash-lamp (μF900) and an integrating sphere, respectively. Single crystal X-ray diffraction data collection of the compounds were recorded by Bruker D8 Venture photon II diffractometer. Gas chromatography (GC) for gas samples were carried out on a SHIMDZU GC-2014ATF/SPL (TDX-01 60/80 mesh, 2.0 mm x 3.2 mm x 2.1 mm-FID, TCD permanent gases, $N_2$ carrier gas), and nitrogen as the carrier gas. Transmission electron microscope (TEM) were recorded by FEI-Tecnai-$G^2$-F30. X-ray powder diffraction (XRD) were collected with a Rigaku SmartLab powder X-ray diffractometer operated at 3 kW. X-ray photoelectron spectroscopy (XPS) were measured using Thermo Fisher ESCALAB Xi+. Photographs were taken using a Nikon D5100 digital camera.

All the computational calculations reported in this work were performed using the Gaussian 09 code. To simulate the experimental UV-Vis in DMF, the Polarizable Continuum Model (PCM)1 as a self-consistent reaction field (SCRF) was used for the calculation of equilibrium geometries, vibrational frequencies and excited state calculations. The geometries for the ground state of these compounds in the DMF solution were optimized at the M06-2X level 2 with the 6-311G(d,p) basis set for all atoms. It should be pointed out that the structures of all stationary points in DMF solvent were fully optimized, and frequency calculations were performed at the same level. The frequency calculations confirmed the nature of all revealed equilibrium geometries: there were no imaginary frequencies.

## 2. Synthetic procedures

### Synthesis of 2.

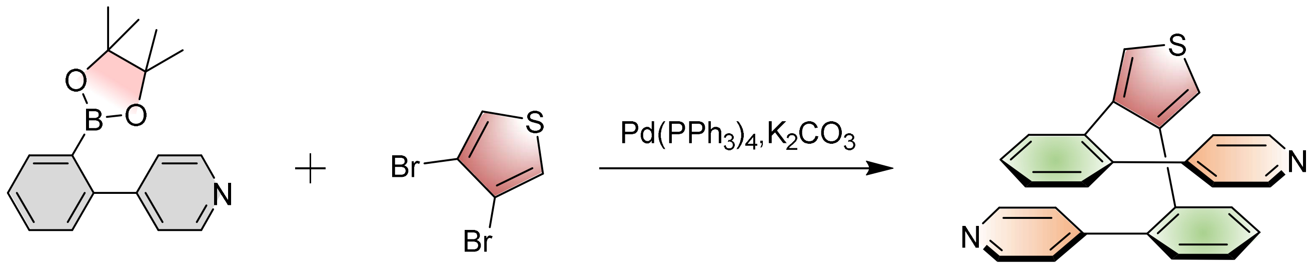


**1** (464.9 mg, 1.65 mmol), 1,2-Diiodobenzene (100 mg, 0.41 mmol), $Pd(PPh_3)_4$ (48 mg, 0.04) and $K_2CO_3$ (229 mg, 1.65 mmol) was added to 50 mL two-necked round bottom flask. The flask was evacuated under vacuum and flushed with dry nitrogen three times and then 25 mL mixture of methylbenzene, ethyl alcohol and deionized water (3/1/1, v/v/v) was added. The reaction mixture was heated and refluxed at 95 °C for 16 h. After cooling to room temperature, the mixture was poured into water and extracted with dichloromethane three times. The combined organic layers were dried over anhydrous magnesium sulfate. After filtration and solvent evaporation, the crude product was purified by silica-gel column chromatography with mixed petroleum ether and ethyl acetate (Yield: 118 mg, 73%).

$^1$H NMR (400 MHz, $CD_2Cl_2$) δ 8.17 (d, $J$ = 4.6 Hz, 4H), 7.25-7.20 (m, 4H), 7.00-6.95 (m, 4H), 6.48 (d, $J$ = 4.6 Hz, 4H), 6.37 (d, $J$ = 7.6 Hz, 2H); $^{13}$C NMR (100 MHz, $CD_2Cl_2$) δ 149.27, 148. 98, 141.86, 137.55, 134.82, 131.72, 129.51, 129.34, 127.72, 125.43, 123.59; HRMS (ESI$^+$) m/z: $[M+H]^+$ calcd for $C_{26}H_{18}N_2S$ 391.12635, found 391.12659; Mp (°C): 218.2°C -219.3°C.

**Synthesis of 3.**

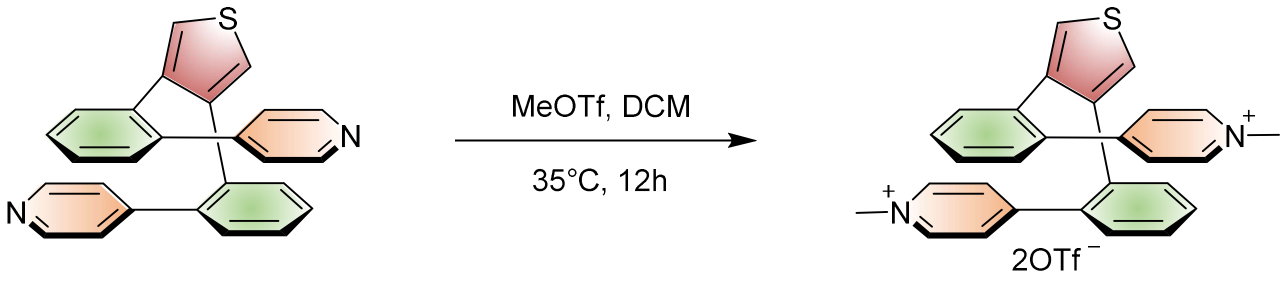


**2** **3**

**2** (98 mg, 0.25 mmol) was dissolved in dichloromethane (5 mL) and cooled the solution to 0 °C, followed by methyl triflate (0.11 mL, 1 mmol) was added. The reaction mixture was stirred at 0 °C for 5 min, then warming up to 35 °C and stirred for 12 h. Then the mixture was cooled to room temperature, and the precipitate was collected and washed with an excess amount of dichloromethane (3 × 5 mL) by centrifuge to obtain white product **3**. (Yield: 142 mg, 79%).

$^1$H NMR (400 MHz, $CD_3CN$) δ 8.20 (dd, *J* = 6.5, 2.3 Hz, 4H), 7.61 (d, *J* = 2.4 Hz, 2H), 7.43 (t, *J* = 7.8 Hz, 2H), 7.45-7.20 (m, 4H), 7.15 (d, *J* = 4.5 Hz, 4H), 6.66 (d, *J* = 7.8 Hz, 2H), 4.22 (s, 6H); $^{13}$C NMR (100 MHz, $CD_3CN$) δ 157.55, 144.81, 139.53, 135.38, 132.91, 132.55, 131.19, 129.56, 128.86, 127.35,48.38; HRMS (ESI$^+$) m/z: [M-2OTf]$^{2+}$ calcd for $C_{28}N_{24}N_2S$ 210.08246, found 210.08270; [M-OTf]$^+$ calcd for $C_{29}N_{24}N_2S_2F_3O_3$ 569.11750; found 569.11703; Mp (°C): 270.1°C -271.2°C.

**Synthesis of 4.**

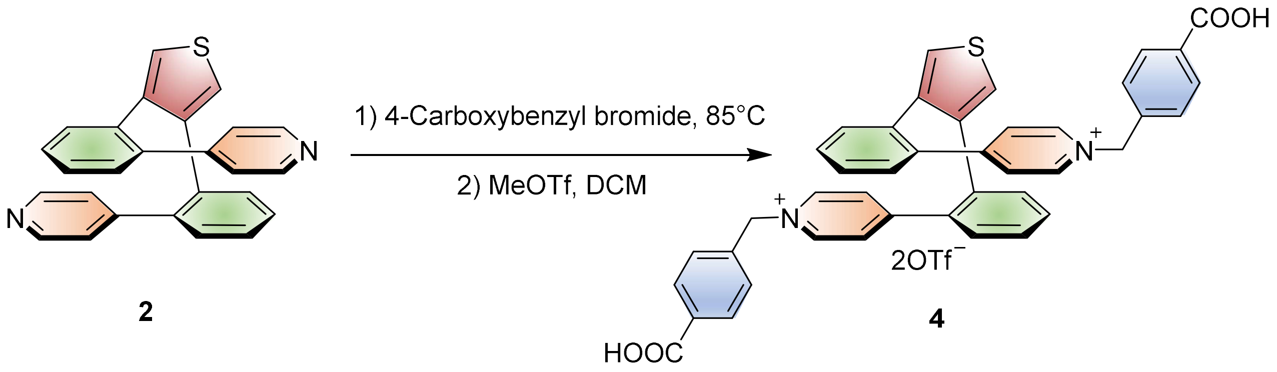


2 4

A mixture of **2** (98 mg, 0.25 mmol) and benzyl bromide (128 mg, 0.75 mmol) in anhydrous MeCN (20 mL) was stirred at 60 ºC for 72h under nitrogen atmosphere. Then the mixture was cooled to room temperature, and the precipitate was collected and washed with an excess amount of acetonitrile (3 × 5 mL) by centrifuge to obtain white product. The product was dissolved in dichloromethane (5 mL) and cooled the solution to 0 °C, followed by methyl triflate (0.11 mL, 1 mmol) was added. The reaction mixture was stirred at 0°C for 5 min, then it was allowed to warm to 35 °C and stirred for 12 h. The precipitate was collected and washed with an excess amount of dichloromethane (3 × 5 mL) by centrifuge to obtain white product **4**. (Yield: 98 mg, 45%).

$^{1}$H NMR (400 MHz, DMSO-$d_6$) δ 13.26 (s, 2H), 8.83 (d, *J* = 6.4 Hz, 4H), 8.11 (d, *J* = 8.1 Hz, 4H), 7.85 – 7.75 (m, 6H), 7.35 – 7.20 (m, 8H), 6.43 (d, *J* = 7.5, 1.4 Hz, 2H), 6.29 (d, *J* = 7.7 Hz, 2H), 5.80 (s, 4H); $^{13}$C NMR (100 MHz, DMSO-$d_6$) δ 166.78, 156.22, 143.51, 138.25, 133.31, 131.84, 131.33, 130.61, 130.21, 129.46, 128.19, 128.02, 126.51, 117.43, 99.54, 62.01; HRMS (ESI$^+$) m/z: [M-2OTf]$^{2+}$ calcd for $C_{42}N_{32}N_2O_4S$ 330.10359; found 330.10429; Mp (°C): > 300°C.

**Synthesis of 5.**

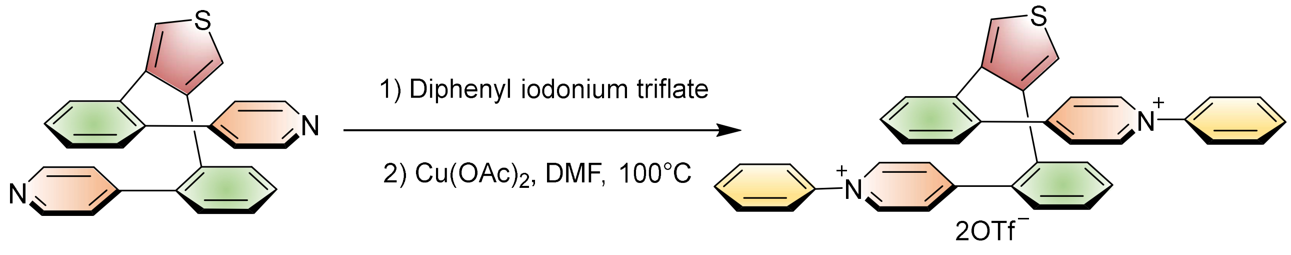


**2** **5**

**2** (98 mg, 0.25mmol), diphenyl iodonium triflate (322.6 mg, 0.75 mmol), and anhydrous $Cu(OAc)_2$ (3 mg, 0.017 mmol) in degassed DMF (4 mL) was stirred at 100 °C for 12 h under nitrogen atmosphere. The volatiles were removed under reduced pressure, the green solid was taken up in acetone/chloroform/diethyl ether (1:1:1), and filtered. The resulting residue was taken up in chloroform/acetone (5:1; 300 mL) and followed by the white solid was collected via centrifuge, washed with cold water and diethyl ether for 3 times, and dried at 40°C under a vacuum to obtain white product **5**. (Yield: 105 mg, 50%)

$^1$H NMR (400 MHz, $CD_3CN$) δ 8.51 (d, $J$ = 6.1 Hz, 4H), 7.80-7.75 (m, 6H), 7.71 (s, 6H), 7.47 (d, $J$ = 7.7 Hz, 2H), 7.38 (dd, $J$ = 15.8, 6.7 Hz, 6H), 7.29 (d, $J$ = 8.1 Hz, 2H), 6.87 (d, $J$ = 7.7 Hz, 2H); $^{13}$C NMR (151 MHz, $CD_3CN$) δ 158.82, 143.91, 143.14, 139.20, 135.71, 134.36, 133.10, 132.90, 132.57, 131.68, 131.61, 129.83, 129.48, 127.64, 125.12.; HRMS (ESI$^+$) m/z: $[M-2OTf]^{2+}$ calcd for $C_{38}N_{28}N_2S$ 273.10594; found 273.10582; Mp (°C): > 300°C.

**Synthesis of 6.**

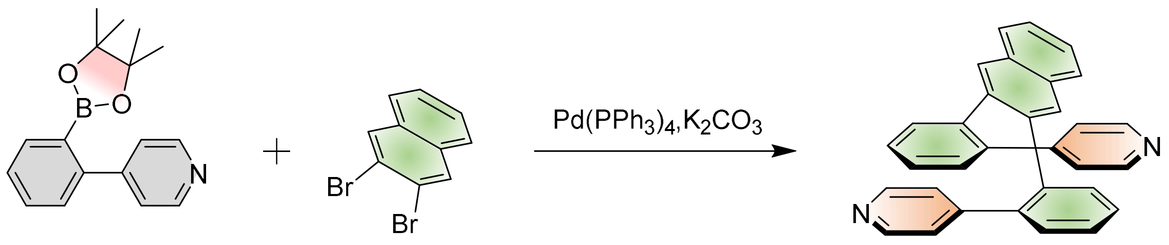


1 6

**1** (393.3 mg, 1.40 mmol), 1,2-Diiodobenzene (100 mg, 0.35 mmol), $Pd(PPh_3)_4$ (40 mg, 0.04) and $K_2CO_3$ (193 mg, 1.40 mmol) was added to 50 mL two-necked round bottom flask. The flask was evacuated under vacuum and flushed with dry nitrogen three times and then 25 mL mixture of methylbenzene, ethyl alcohol and deionized water (3/1/1, v/v/v) was added. The reaction mixture was heated and refluxed at 95 °C for 16 h. After cooling to room temperature, the mixture was poured into water and extracted with dichloromethane three times. The combined organic layers were dried over anhydrous magnesium sulfate. After filtration and solvent evaporation, the crude product was purified by silica-gel column chromatography with mixed petroleum ether and ethyl acetate (Yield: 76 mg, 50%).

$^1$H NMR (400 MHz, $CD_2Cl_2$) δ 8.11 (d, *J* = 5.1 Hz, 4H), 7.92 – 7.90 (m, 2H), 7.79 (s, 2H), 7.56-7.54 (m, 2H), 7.25 (t, *J* = 7.3 Hz, 2H), 7.04-6.99 (m, 4H), 6.44 (d, *J* = 4.4 Hz, 4H), 6.29 (d, *J* = 7.6 Hz, 2H); $^{13}$C NMR (100 MHz, $CD_2Cl_2$) δ 149.38, 148.77, 139.45, 139.04, 137.88, 133.45, 132.83, 131.14, 129.31, 128.10, 127.65, 126.83, 123.85; HRMS (ESI$^+$) m/z: $[M+H]^+$ calcd for $C_{32}H_{22}N_2$ 435.18558; found 135.18779; Mp (°C): 224.2°C -225.2°C.

**Synthesis of 7.**

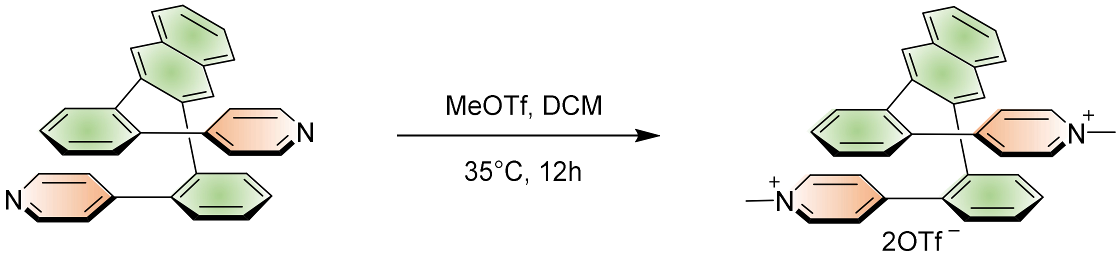


**6** (109 mg, 0.25 mmol) was dissolved in dichloromethane (5 mL) and cooled the solution to 0 °C, followed by methyl triflate (0.11 mL, 1 mmol) was added. The reaction mixture was stirred at 0 °C for 5 min, then warmed up to 35 °C and stirred for 12 h. Then the mixture was cooled to room temperature, and the precipitate was collected and washed with an excess amount of dichloromethane (3 × 5 mL) by centrifuge to obtain white product **7**. (Yield: 143 mg, 75%).

$^{1}$H NMR (400 MHz, $CD_3CN$) δ 8.17 (d, *J* = 6.3 Hz, 4H), 8.08-8.04 (m, 4H), 7.68 (dd, *J* = 6.4, 3.3 Hz, 2H), 7.47 (t, *J* = 7.6 Hz, 2H), 7.32-7.26 (m, 4H), 7.14 (d, *J* = 6.3 Hz, 4H), 6.68 (d, *J* = 7.7 Hz, 2H), 4.20 (s, 6H); $^{13}$C NMR (100 MHz, $CD_3CN$) δ 157.54, 145.16, 140.13, 136.77, 135.32, 134.40, 132.96, 131.11, 129.59, 128.99, 128.52, 127.67, 48.56; HRMS (ESI$^{+}$) m/z: [M-2OTf]$^{2+}$ calcd for $C_{34}H_{38}N_2$ 233.11990; found 233.11525; [M-OTf]$^{+}$ calcd for $C_{35}H_{28}N_2F_3SO_3$ 613.17672; found 613.17604; Mp (°C): 270.4°C - 271.3°C.

**Synthesis of 8.**

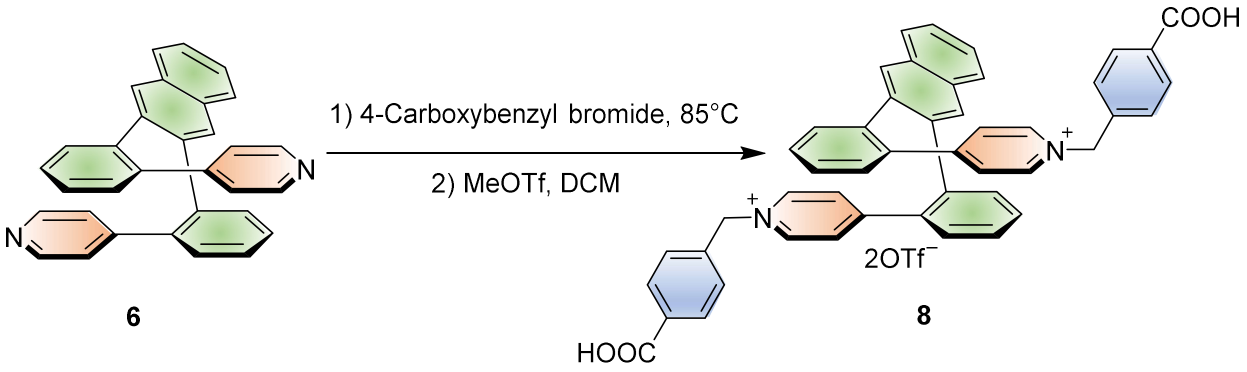


A mixture of **6** (109 mg, 0.25 mmol) and benzyl bromide (128 mg, 0.75 mmol) in anhydrous MeCN (20 mL) was stirred at 60 ºC for 72h under nitrogen atmosphere. Then the mixture was cooled to room temperature, and the precipitate was collected and washed with an excess amount of acetonitrile (3 × 5 mL) by centrifuge to obtain white product. The product was dissolved in dichloromethane (5 mL) and cooled the solution to 0 °C, followed by methyl triflate (0.11 mL, 1 mmol) was added. The reaction mixture was stirred at 0°C for 5 min, then it was allowed to warm to 35 °C and stirred for 12 h. The precipitate was collected and washed with an excess amount of dichloromethane (3 × 5 mL) by centrifuge to obtain white product **8**. (Yield: 91 mg, 40%).

$^{1}$H NMR (400 MHz, DMSO-$d_6$) δ 13.26 (s, 2H), 8.79 (d, $J$ = 6.3 Hz, 4H), 8.10-8.05 (m, 8H), 7.73 (d, $J$ = 8.0 Hz, 4H), 7.67 (dd, $J$ = 6.3, 3.3 Hz, 2H), 7.34 (d, $J$ = 7.5 Hz, 2H), 7.28 (d, $J$ = 7.8 Hz, 2H), 7.23 (d, $J$ = 6.3 Hz, 4H), 6.51 (t, $J$ = 7.6 Hz, 2H), 6.35 (d, $J$ = 7.7 Hz, 2H), 5.78 (s, 4H); $^{13}$C NMR (100 MHz, DMSO-$d_6$) δ 166.77, 155.91, 143.86, 138.66, 138.60, 135.38, 133.14, 131.80, 131.74, 131.11, 130.39, 130.19, 129.37, 128.10, 127.34, 126.58, 62.11; HRMS (ESI$^{+}$) m/z: [M-2OTf]$^{2+}$ calcd for $C_{48}H_{36}N_2$ 320.14338; found 320.12164; [M-OTf]$^{+}$ calcd for $C_{49}H_{36}N_2F_3SO_3$ 790.24715; found 790.24388; Mp (°C): > 300°C.

**Synthesis of 9.**

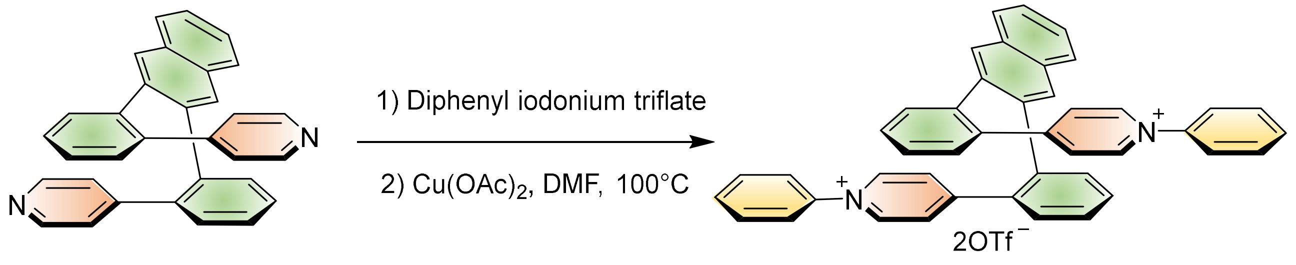


6 9

**6** (109 mg, 0.25mmol), diphenyl iodonium triflate (322.6 mg, 0.75 mmol), and anhydrous $Cu(OAc)_2$ (3 mg, 0.017 mmol) in degassed DMF (4 mL) was stirred at 100 °C for 12 h under nitrogen atmosphere. The volatiles were removed under reduced pressure, the green solid was taken up in acetone/chloroform/diethyl ether (1:1:1), and filtered. The resulting residue was taken up in chloroform/acetone (5:1; 300 mL) and followed by the white solid was collected via centrifuge, washed with cold water and diethyl ether for 3 times, and dried at 40°C under a vacuum to obtain white product **9**. (Yield: 104 mg, 47%).

$^1$H NMR (400 MHz, $CD_3CN$) δ 8.48 (d, *J* = 7.0 Hz, 4H), 8.15-8.05 (m, 4H), 7.76-7.73 (m, 6H), 7.75 – 7.65 (m, 6H), 7.53 (td, *J* = 7.6, 1.3 Hz, 2H), 7.40-7.35 (m, 6H), 7.31 (td, *J* = 7.6, 1.4 Hz, 2H), 6.89 (dd, *J* = 7.8, 1.3 Hz, 2H); $^{13}$C NMR (151 MHz, $CD_3CN$) δ 158.64, 144.18, 143.16, 140.32, 136.31, 136.31, 135.08, 135.08, 134.65, 134.65, 133.48, 133.48, 133.05, 133.05, 132.56, 132.56, 131.55, 131.55, 131.52, 129.80, 129.04, 128.54, 127.70, 125.15; HRMS (ESI$^+$) m/z: [M-2OTf]$^{2+}$ calcd for $C_{44}H_{32}N_2$ 294.12773; found 294.12749; [M-OTf]$^+$ calcd for $C_{45}H_{32}N_2F_3O_3S$ 737.20802; found 737.20987; Mp (°C): > 300°C.

## Abbreviation name of compound

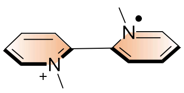

**V-Me$^{+\bullet}$**

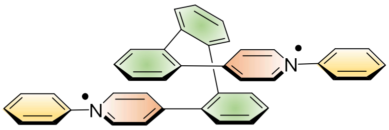

**TSC-V-Ph''**

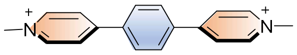

**V-Ph$^{2+}$**

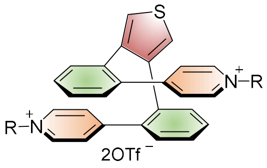


**TSC-TV$^{2+}$**

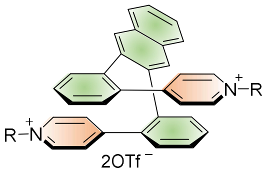


**TSC-NV$^{2+}$**

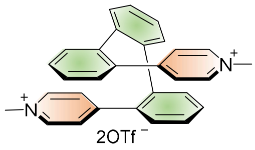


**TSC-V-Me$^{2+}$**

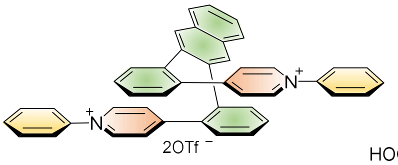


**TSC-V-Ph$^{2+}$**

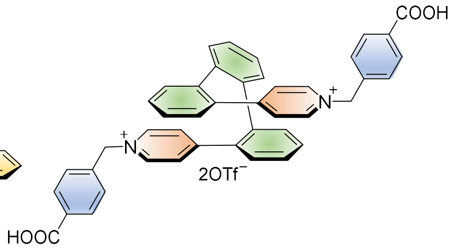


**TSC-V-pTA$^{2+}$**

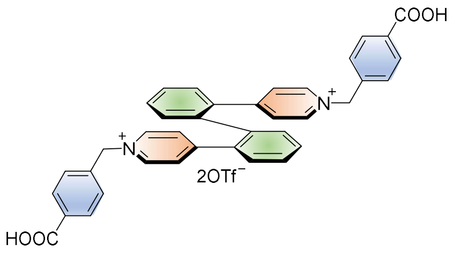


**V-pTA$^{2+}$**

**3. Single-crystal X-ray structure determination.**

**X-ray Crystallography.** X-ray diffraction data collection of the compounds were recorded by BrukerD8 Verture system with PHOTON II CPAD detector equipped at 150 K or 193 K and a Ga-target Liquid Metal Source ($\lambda$ = 1.34139 Å). The structures were solved by SHELXT (version 2018/2) and refined by full-matrix least-squares procedures using the SHELXL program (version 2018/3) through the OLEX2 graphical interface.

**The crystal of 2: 2** (2.0 mg, 5.0 mmol) was dissolved in $CHCl_3$ (10 mL) and the solution was passed through a 0.45-µm filter into a 5-mL bottle (2 mL solution in each bottle). The bottle was capped, after slow evaporation of MeCN at room temperature for 7 days, and light-white block-like single crystals of **2** were obtained.

**The crystal of 3: 3** (3.6 mg, 5.0 mmol) was dissolved in $CHCl_3$ (10 mL) and the solution was passed through a 0.45-µm filter into a 5-mL bottle (2 mL solution in each bottle). The bottle was capped, after slow evaporation of MeCN at room temperature for 7 days, and light-white block-like single crystals of **3** were obtained.

**The crystal of 6: 6** (4.2 mg, 5.0 mmol) was dissolved in MeCN (10 mL) and the solution was passed through a 0.45-µm filter into a 10-mL bottle (2 mL solution in each bottle). The bottle was capped, after slow evaporation of MeCN at room temperature for 7 days, and light-white block-like single crystals of **6** were obtained.

**The crystal of 8: 8** (4.2 mg, 5.0 mmol) was dissolved in MeCN (10 mL) and the solution was passed through a 0.45-µm filter into a 10-mL bottle (2 mL solution in each bottle). The bottle was capped, after slow evaporation of MeCN at room temperature for 7 days, and light-white block-like single crystals of **8** were obtained.

**The crystal of 9: 9** (5.1 mg, 5.1 mmol) was dissolved in MeCN (10 mL) and the solution was passed through a 0.45-µm filter into a 10-mL bottle (2 mL solution in each bottle). The bottle was capped, after slow evaporation of MeCN at room temperature for 7 days, and light-yellow block-like single crystals of **9** were obtained.

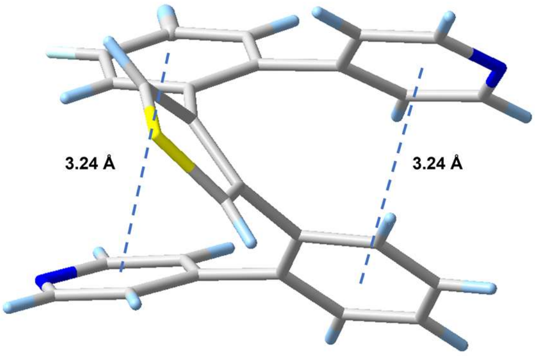


**Figure S1.** X-ray crystal structure of **2**. Color code: C, grey; N, blue; S, yellow; H, light blue. Counter ions and solvent molecules have been omitted.

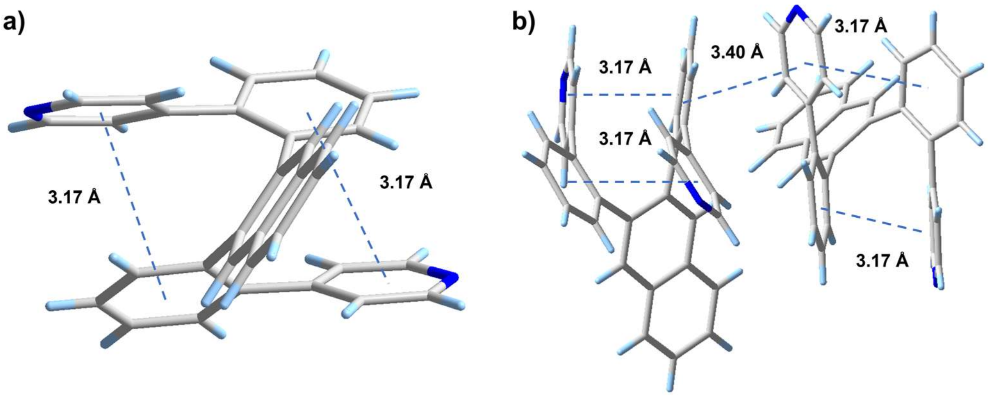


**Figure S2.** X-ray crystal structure of **6**. Color code: C, grey; N, blue; H, light blue. Counter ions and solvent molecules have been omitted.

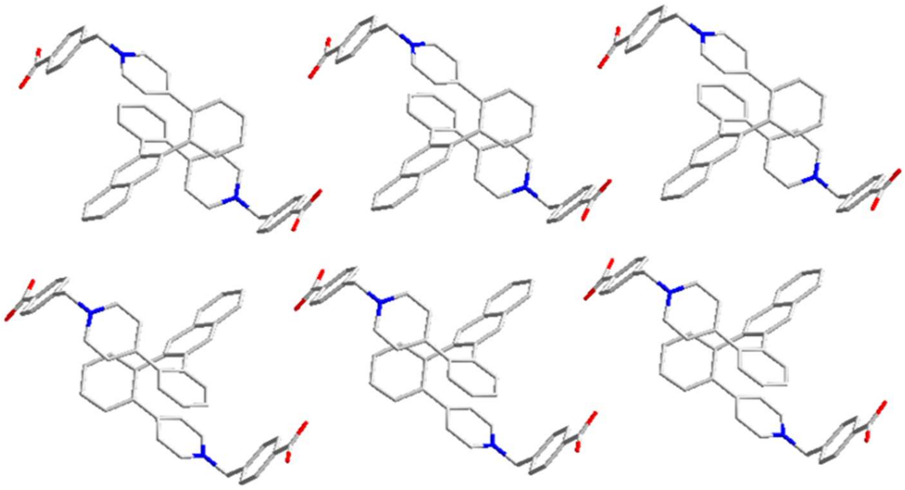

**Figure S3.** X-ray crystal structure of **8**. Color code: C, grey; N, blue; O, red. All counter ions, solvent molecules, and hydrogen atoms have been omitted for clarity.

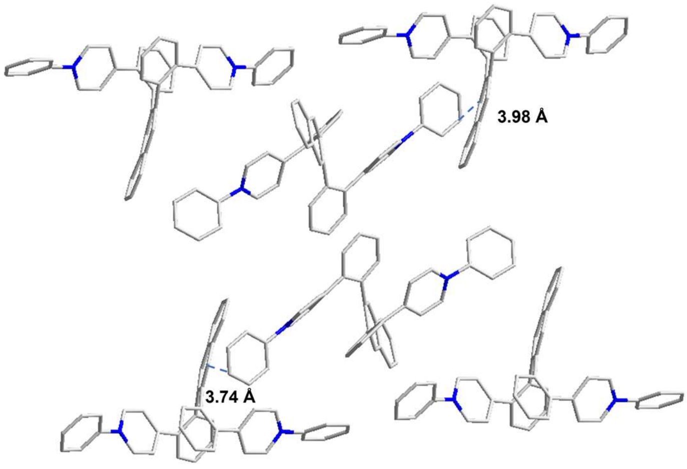


**Figure S4.** X-ray crystal structure of **9**. Color code: C, grey; N, blue; O, red. All counter ions, solvent molecules, and hydrogen atoms have been omitted for clarity.

**Table S1.** Crystal data and structure refinement for compound **2** (CCDC 2358118).

| | |
|---|---|
| Empirical formula | $C_{26}$ $H_{18}$ $N_2$ S |
| Formula weight | 390.48 |
| Temperature | 150.00 K |
| Wavelength | 0.71073 Å |
| Crystal system, space group | Triclinic, P-1 |
| Unit cell dimensions | a = 9.5416(6) Å α = 101.102(3)° |
| | b = 13.7930(10) Å β = 100.129(2)° |
| | c = 15.3956(11) Å γ = 93.034(2)° |
| Volume | 1949.4(2) Å^3 |
| Z, Calculated density | 4, 1.331 Mg/m^3 |
| Absorption coefficient | 0.181 mm^-1 |
| F(000) | 816.0 |
| Crystal size | 0.15 × 0.13 × 0.12 mm |
| Theta range for data collection | 3.642 to 55.02° |
| Limiting indices | -10 ≤ h ≤ 12, -17 ≤ k ≤ 17, -19 ≤ l ≤ 19 |
| Reflections collected / unique | 43390 / 8942 [Rint = 0.0820] |
| Completeness to θ = 25.242° | 99.9% |
| Max. and min. transmission | 0.7456 and 0.6976 |
| Refinement method | Full-matrix least-squares on F^2 |
| Data / restraints / parameters | 8942 / 0 / 523 |
| Goodness-of-fit on F^2 | 1.062 |
| Final R indices [I>2sigma(I)] | R1 = 0.0476, wR2 = 0.1071 |
| R indices (all data) | R1 = 0.0684, wR2 = 0.1255 |
| Extinction coefficient | n/a |
| Largest diff. peak and hole | 0.28 and -0.47 e. A^-3 |

**Table S2.** Crystal data and structure refinement for compound **3** (CCDC 2358119).

| | |
|---|---|
| Empirical formula | $C_{30} H_{24} F_6 N_2 O_6 S_3$ |
| Formula weight | 718.69 |
| Temperature | 100.00 K |
| Wavelength | 1.54178 Å |
| Crystal system, space group | Triclinic, P-1 |
| Unit cell dimensions | a = 10.3901(2) Å α = 68.6420(10)° |
| | b = 12.4184(3) Å β = 82.9270(10)° |
| | c = 13.5201(3) Å γ = 69.4790(10)° |
| Volume | 1521.56(6) Å ^3 |
| Z, Calculated density | 2, 1.569 Mg/m^3 |
| Absorption coefficient | 2.994 mm^-1 |
| F(000) | 736.0 |
| Crystal size | 0.15 × 0.13 × 0.12 mm |
| Theta range for data collection | 7.02 to 140.564° |
| Limiting indices | -12 ≤ h ≤ 12, -15 ≤ k ≤ 15, -16 ≤ l ≤ 16 |
| Reflections collected / unique | 40774 / 5766 [Rint = 0.0671] |
| Completeness to θ = 67.679° | 99.7% |
| Max. and min. transmission | 0.7533 and 0.6567 |
| Refinement method | Full-matrix least-squares on F^2 |
| Data / restraints / parameters | 5766 / 0 / 426 |
| Goodness-of-fit on F^2 | 1.071 |
| Final R indices [I>2sigma(I)] | R1 = 0.0431, wR2 = 0.1056 |
| R indices (all data) | R1 = 0.0499, wR2 = 0.1094 |
| Extinction coefficient | n/a |
| Largest diff. peak and hole | 1.08 and -0.50 e. A^-3 |

**Table S3.** Crystal data and structure refinement for compound **6** (CCDC 2358120).

| | |
|---|---|
| Empirical formula | $C_{32}H_{22}N_2$ |
| Formula weight | 434.56 |
| Temperature | 150.00 K |
| Wavelength | 0.71073 Å |
| Crystal system, space group | Monoclinic, $P2_1/c$ |
| Unit cell dimensions | a = 9.1938(4) Å α = 90° |
| | b = 39.441(2) Å β = 96.804(2)° |
| | c = 13.2161(7) Å γ = 90° |
| Volume | 4758.6(4) Å ^3 |
| Z, Calculated density | 8, 1.213 Mg/m^3 |
| Absorption coefficient | 0.071 mm^-1 |
| F(000) | 1824.0 |
| Crystal size | 0.16 × 0.14 × 0.13 mm |
| Theta range for data collection | 4.13 to 54.998° |
| Limiting indices | -10 ≤ h ≤ 11, -44 ≤ k ≤ 51, -16 ≤ l ≤ 17 |
| Reflections collected / unique | 53537 / 10885 [Rint = 0.0924] |
| Completeness to θ = 25.242° | 100.0% |
| Max. and min. transmission | 0.7456 and 0.6415 |
| Refinement method | Full-matrix least-squares on F^2 |
| Data / restraints / parameters | 10885 / 0 / 613 |
| Goodness-of-fit on F^2 | 1.041 |
| Final R indices [I>2sigma(I)] | R1 = 0.0699, wR2 = 0.1692 |
| R indices (all data) | R1 = 0.0971, wR2 = 0.1882 |
| Extinction coefficient | n/a |
| Largest diff. peak and hole | 0.41 and -0.29 e. A^-3 |

**Table S4.** Crystal data and structure refinement for compound **8** (CCDC 2358121).

| | |
|---|---|
| Empirical formula | $C_{102}$ $H_{75}$ $F_{12}$ $N_5$ $O_{20}$ $S_4$ |
| Formula weight | 2046.91 |
| Temperature | 193.00 K |
| Wavelength | 1.34139 Å |
| Crystal system, space group | Triclinic, P-1 |
| Unit cell dimensions | a = 13.747(4) Å α = 76.775(14)° |
| | b = 14.267(6) Å β = 81.439(8)° |
| | c = 27.719(8) Å γ = 88.527(10)° |
| Volume | 5233(3) Å ^3 |
| Z, Calculated density | 2, 1.299 Mg/m^3 |
| Absorption coefficient | 1.042 mm^-1 |
| F(000) | 2108.0 |
| Crystal size | 0.12 × 0.1 × 0.08 mm |
| Theta range for data collection | 2.88 to 107.812° |
| Limiting indices | -16 ≤ h ≤ 16, -17 ≤ k ≤ 16, -33 ≤ l ≤ 33 |
| Reflections collected / unique | 76066 / 19001 [Rint = 0.0610] |
| Completeness to θ = 53.594° | 99.2% |
| Max. and min. transmission | 0.7519 and 0.6121 |
| Refinement method | Full-matrix least-squares on F^2 |
| Data / restraints / parameters | 19001 / 676 / 1434 |
| Goodness-of-fit on F^2 | 1.065 |
| Final R indices [I>2sigma(I)] | R1 = 0.1260, wR2 = 0.2964 |
| R indices (all data) | R1 = 0.1554, wR2 = 0.3152 |
| Extinction coefficient | n/a |
| Largest diff. peak and hole | 1.00 and -0.93 e. A^-3 |

**Table S5.** Crystal data and structure refinement for compound **9** (CCDC 2358122)

| | |
|---|---|
| Empirical formula | $C_{48} H_{35} F_6 N_3 O_6 S_2$ |
| Formula weight | 927.91 |
| Temperature | 193.00 K |
| Wavelength | 0.71073 Å |
| Crystal system, space group | Monoclinic, P 1 21/n 1 |
| Unit cell dimensions | a = 13.9322(8) Å α = 90° |
| | b = 22.2531(11) Å β = 100.094(2)° |
| | c = 14.1250(6) Å γ = 90° |
| Volume | 4263.1(4) Å ^3 |
| Z, Calculated density | 4, 1.446 Mg/m^3 |
| Absorption coefficient | 0.206 mm^-1 |
| F(000) | 1912 |
| Crystal size | 0.12 x 0.1 x 0.08 mm |
| Theta range for data collection | 1.758 to 25.349° |
| Limiting indices | -16<=h<=11, -26<=k<=26, -16<=l<=17 |
| Reflections collected / unique | 33572 / 7808 [R(int) = 0.0995] |
| Completeness to θ = 53.594° | 100.0% |
| Max. and min. transmission | 0.7456 and 0.6288 |
| Refinement method | Full-matrix least-squares on F^2 |
| Data / restraints / parameters | 7808 / 0 / 587 |
| Goodness-of-fit on F^2 | 1.038 |
| Final R indices [I>2sigma(I)] | R1 = 0.0632, wR2 = 0.1105 |
| R indices (all data) | R1 = 0.1353, wR2 = 0.1364 |
| Extinction coefficient | n/a |
| Largest diff. peak and hole | 0.290 and -0.293 e. A^-3 |

**4. Optical characterization data**

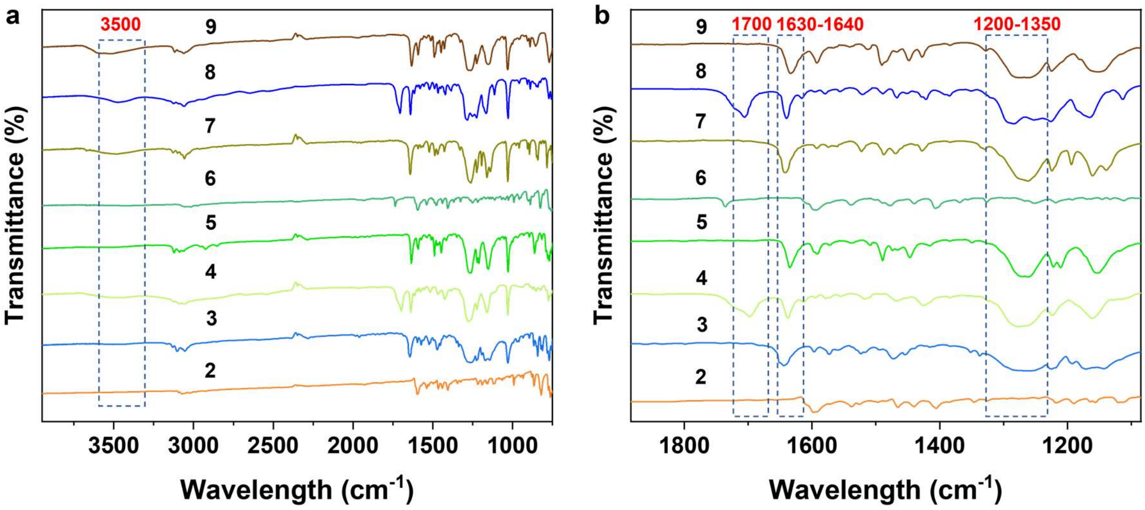


**Figure S5.** FT-IR spectra of **2-9** at room temperature.

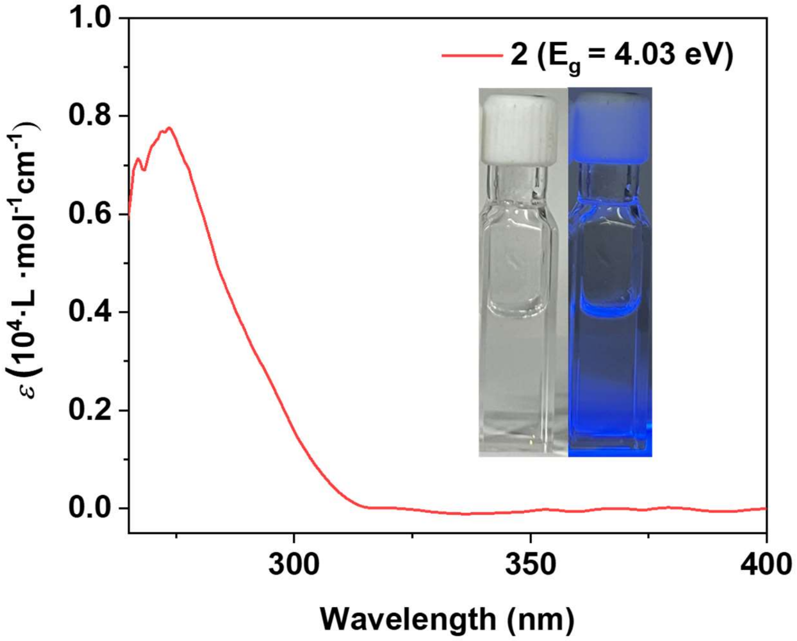


**Figure S6.** UV/vis absorption of **2** at room temperature in DMF solution. The inset photographs of **2** in DMF upon daylight and UV light (365 nm).

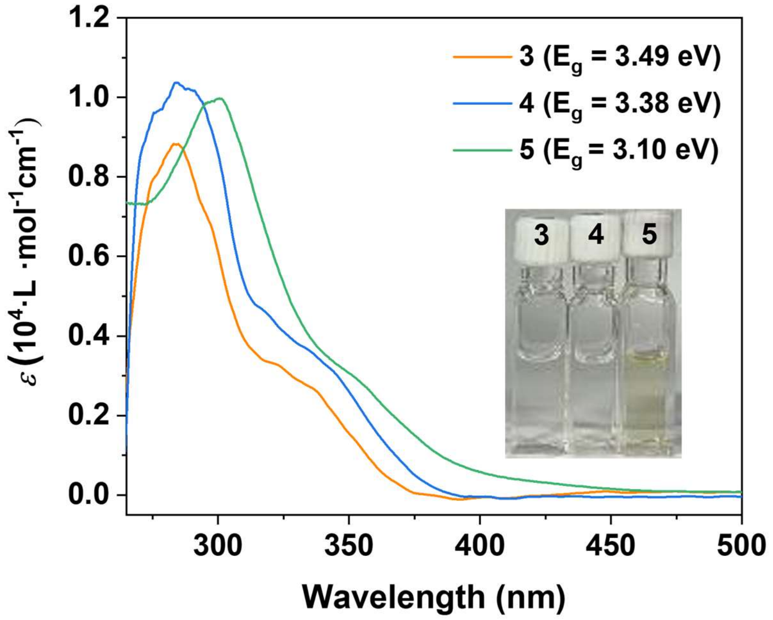


**Figure S7.** UV/vis absorption of **3**, **4** and **5** at room temperature in DMF solution. The inset photographs of **3**, **4** and **5** in DMF upon daylight and UV light (365 nm).

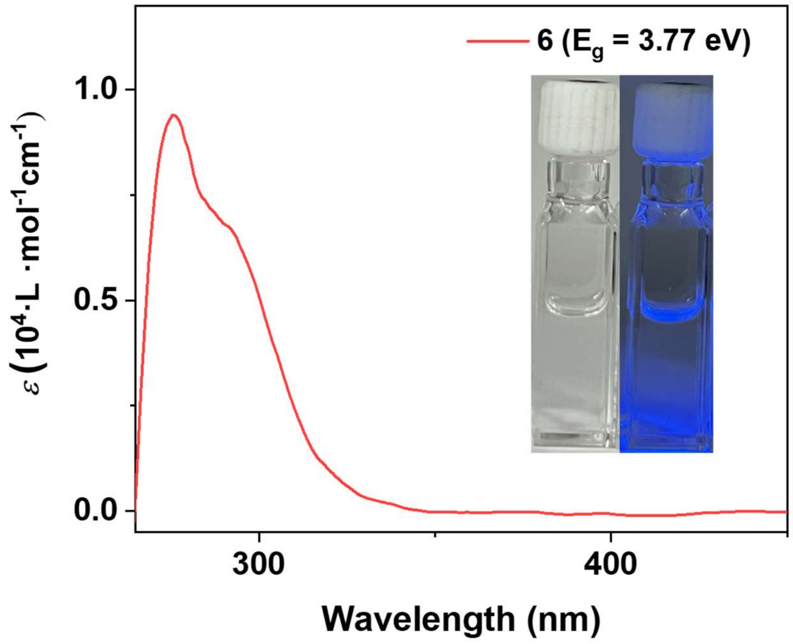


**Figure S8.** (a) UV/vis absorption of **6** at room temperature in DMF solution. The inset photographs of **6** in DMF upon daylight and UV light (365 nm).

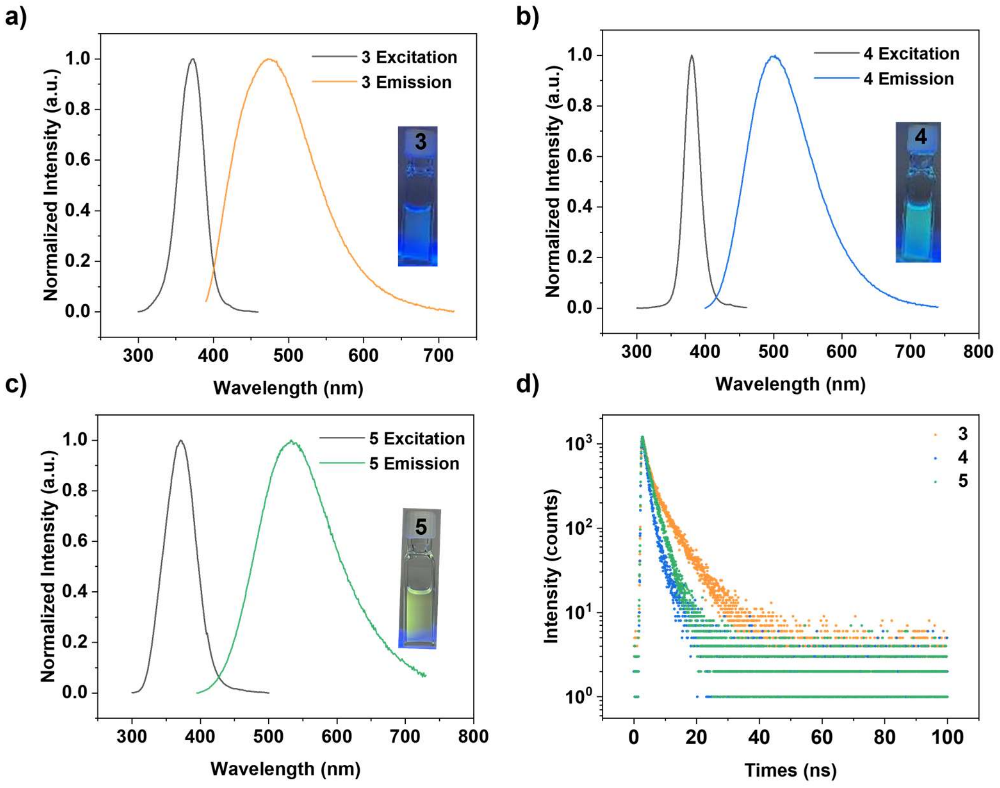


**Figure S9.** Fluorescence spectra of (a) **3**, (b) **4** and (c) **5** at room temperature in DMF (c= 2.0 × $10^{-5}$ M); (d) lifetime decay profiles of phosphorescence bands of **3**, **4** and **5** in DMF (c = 2 × $10^{-5}$ M) under ambient conditions. Inset: the fluorescence images of **3**, **4** and **5** in acetonitrile upon UV irradiation (365 nm).

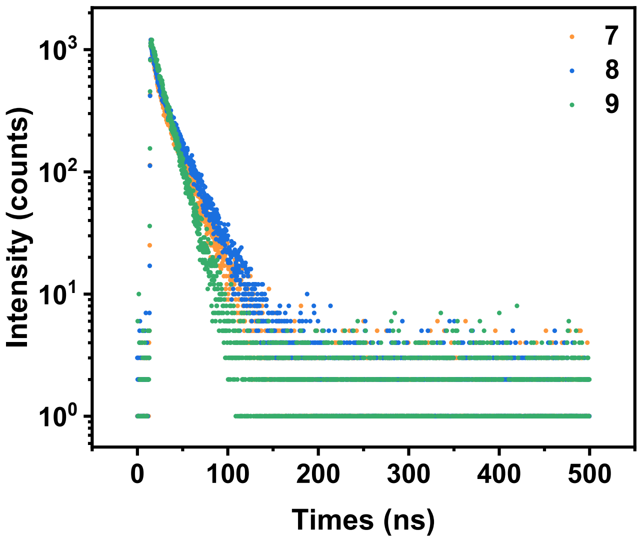


**Figure S10.** Lifetime decay profiles of phosphorescence bands of **7**, **8** and **9** in DMF (c = 2 × $10^{-5}$ M) under ambient conditions.

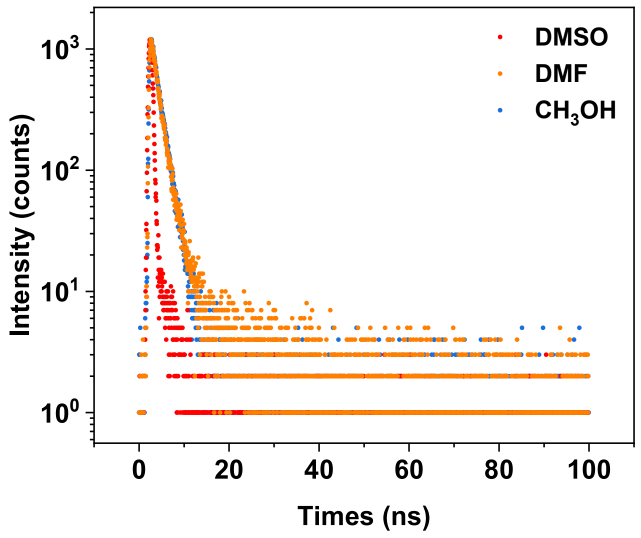


**Figure S11.** Lifetime decay profiles of phosphorescence bands of **5** in different solutions (DMSO, $CH_3OH$ and DMF) (c = 2 × $10^{-5}$ M) under ambient conditions.

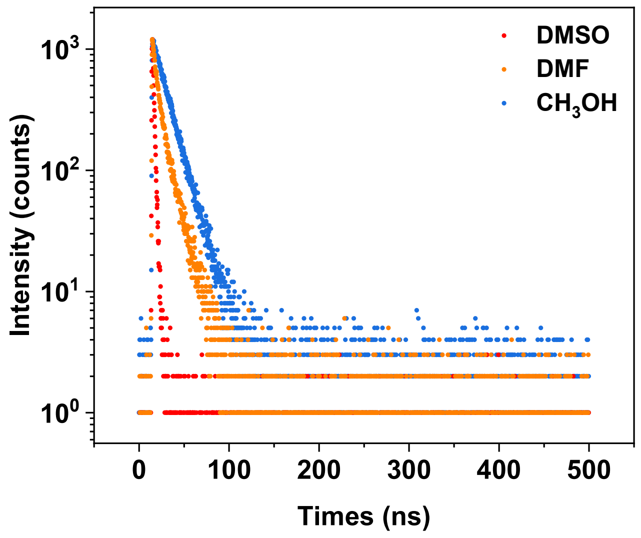


**Figure S12.** Lifetime decay profiles of phosphorescence bands of **9** in different solutions (DMSO, $CH_3OH$ and DMF) (c = 2 × $10^{-5}$ M) under ambient conditions.

**Table S6.** Photophysical average lifetimes (τ) of **3**, **4**, **5**, **7**, **8** and **9** in DMSO, $CH_3OH$ and DMF.

| Compound | Solution | lifetimes (τ/ns) |
|---|---|---|
| **3** | DMF | 5.40 |
| **4** | DMF | 2.88 |
| **5** | DMF | 2.84 |
| **5** | DMSO | 0.40 |
| **5** | $CH_3OH$ | 1.76 |
| **7** | DMF | 21.82 |
| **8** | DMF | 22.95 |
| **9** | DMF | 15.62 |
| **9** | DMSO | 1.62 |
| **9** | $CH_3OH$ | 15.62 |

**Table S7.** Quantum yield ($\Phi_F$) of **3**, **4**, **5, 7, 8** and **9** in DMF.

| Compound | $\Phi_F$ |
|---|---|
| **3** | 1.55% |
| **4** | 3.41% |
| **5** | 2.84% |
| **7** | 28.19% |
| **8** | 27.62% |
| **9** | 14.83% |

## 5. Femtosecond transient absorption measurements

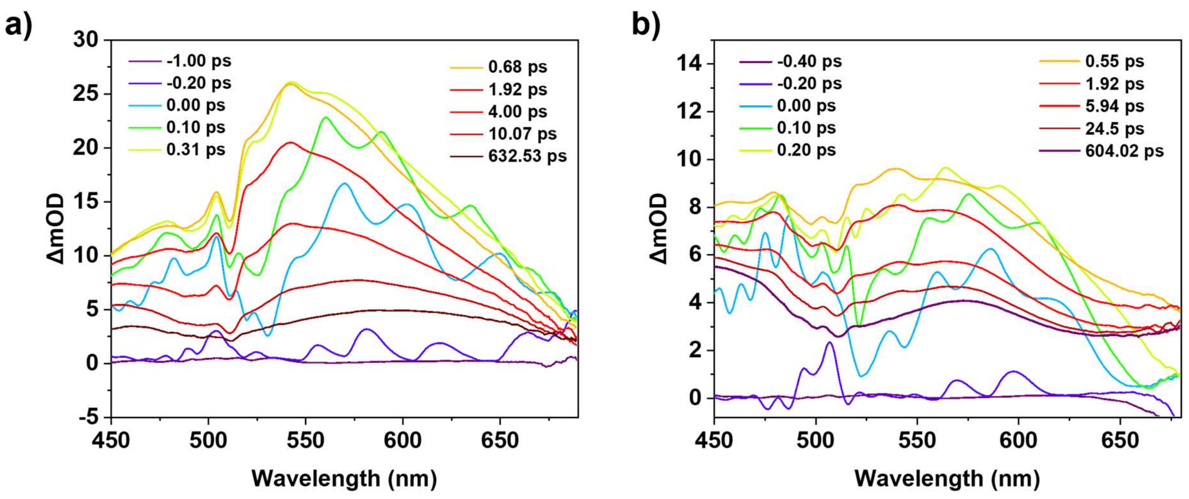


**Figure S13**. Transient absorption spectra of **5** (a) and **9** (b) in DMF.

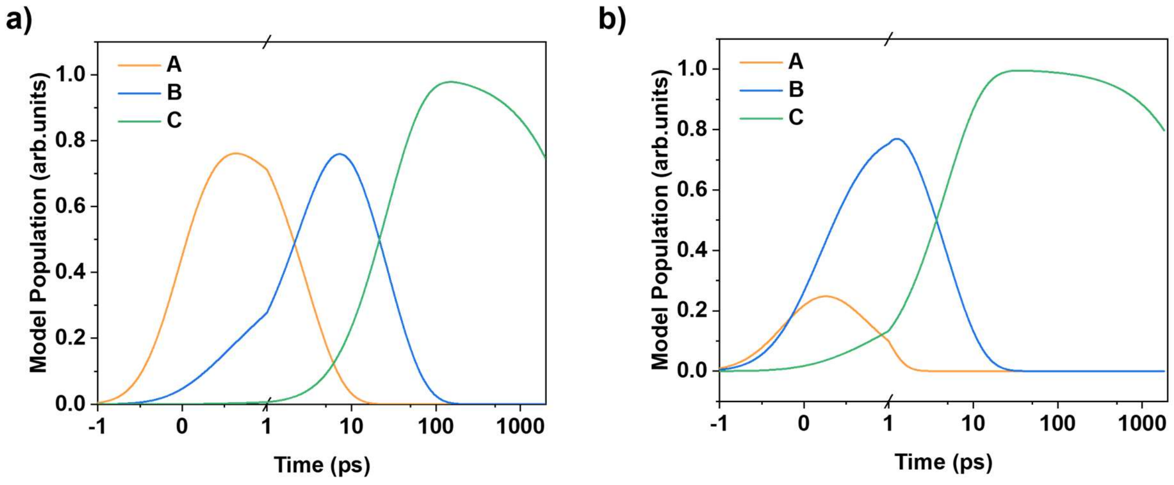


**Figure S14**. Population model kinetic profiles of **5** (a) and **9** (b).

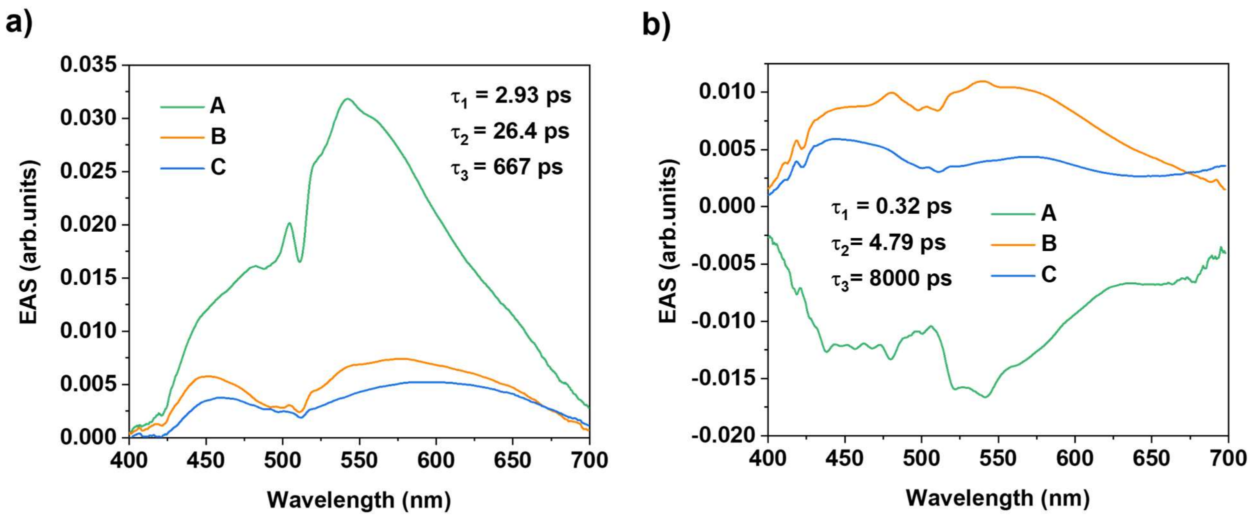


**Figure S15.** Evolution-associated spectra for an A → B → C → ground state sequential decay model of **5** (a) and **9** (b).

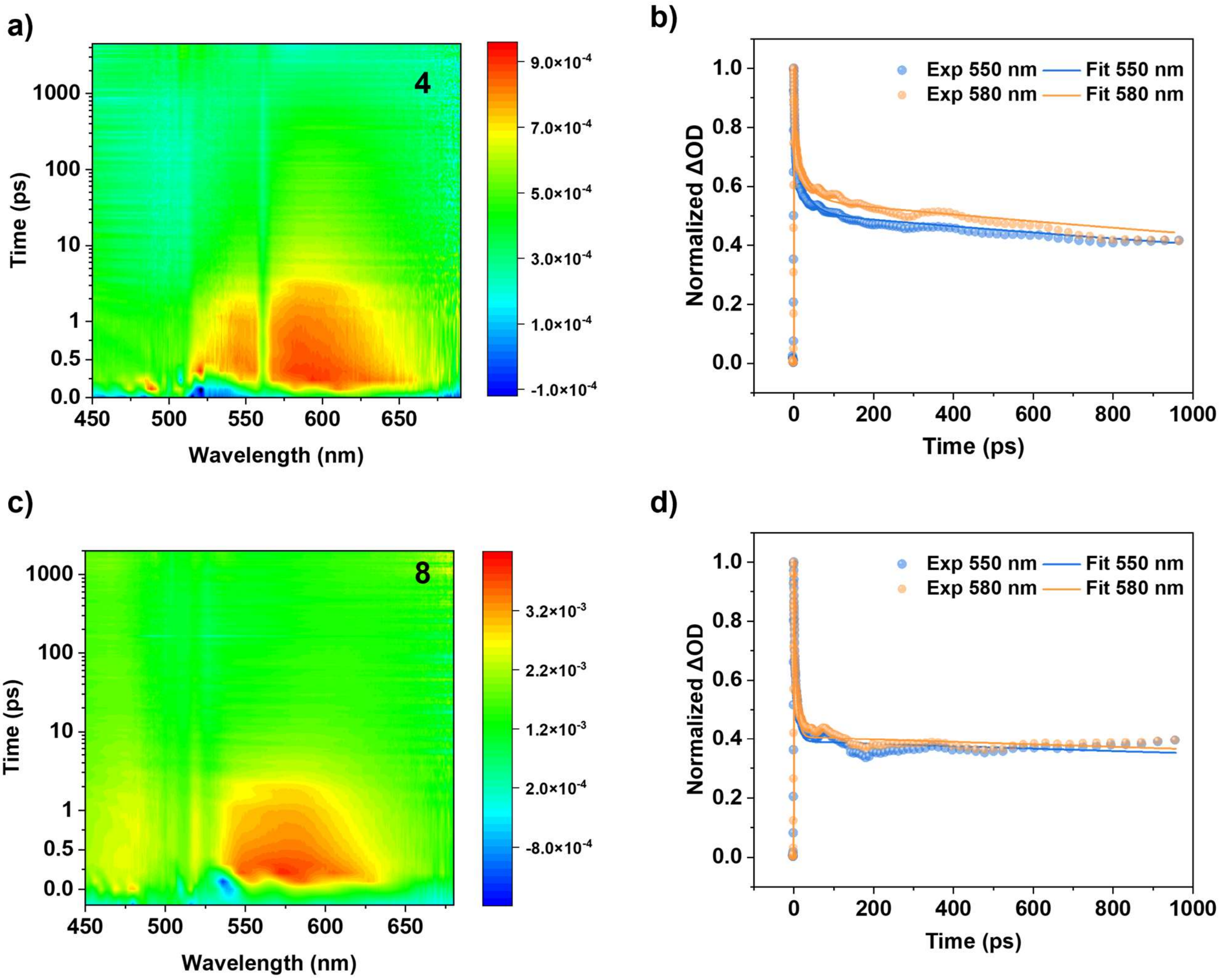


**Figure S16**. Transient absorption spectra (a) and (c), Contour map of **4** and **8** in DMF. (b) and (d) Decay curves in transient absorption of **4** and **8** probed at 550 nm and 580 nm.

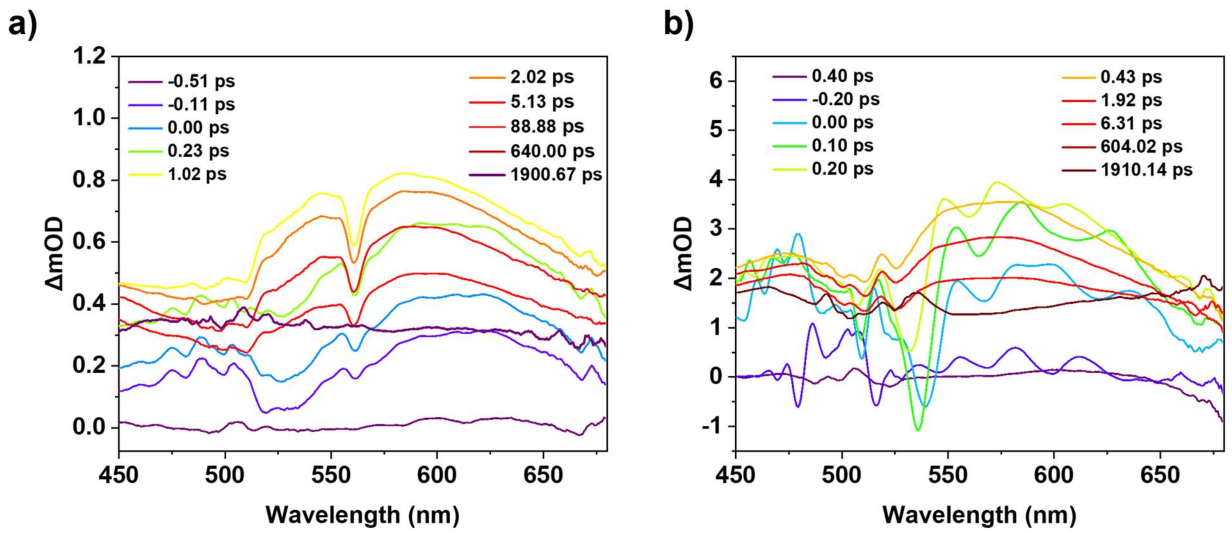


**Figure S17**. Transient absorption spectra of **4** (a) and **8** (b) in DMF.

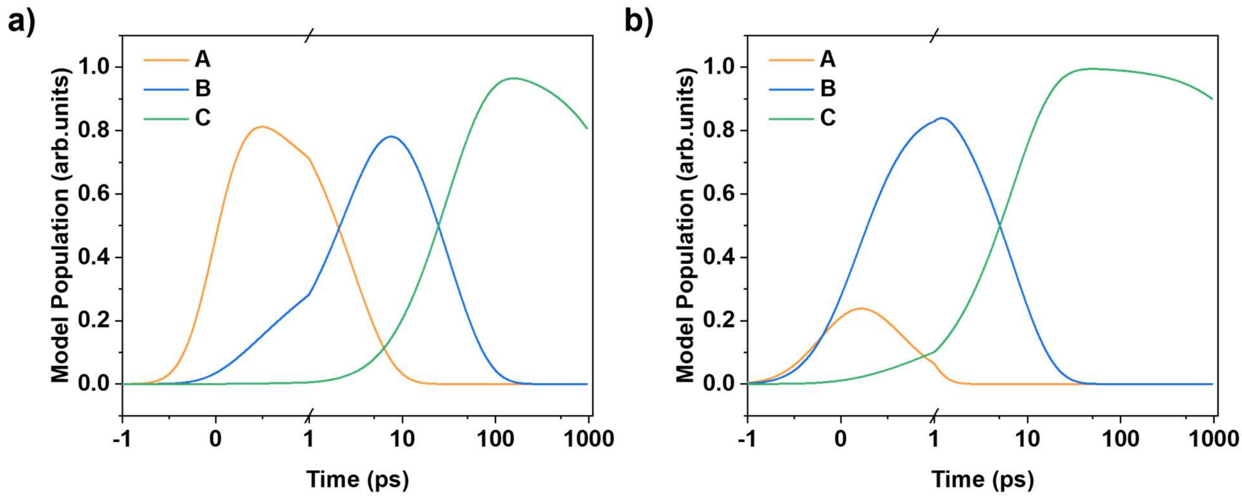


**Figure S18**. Population model kinetic profiles of **4** (a) and **8** (b).

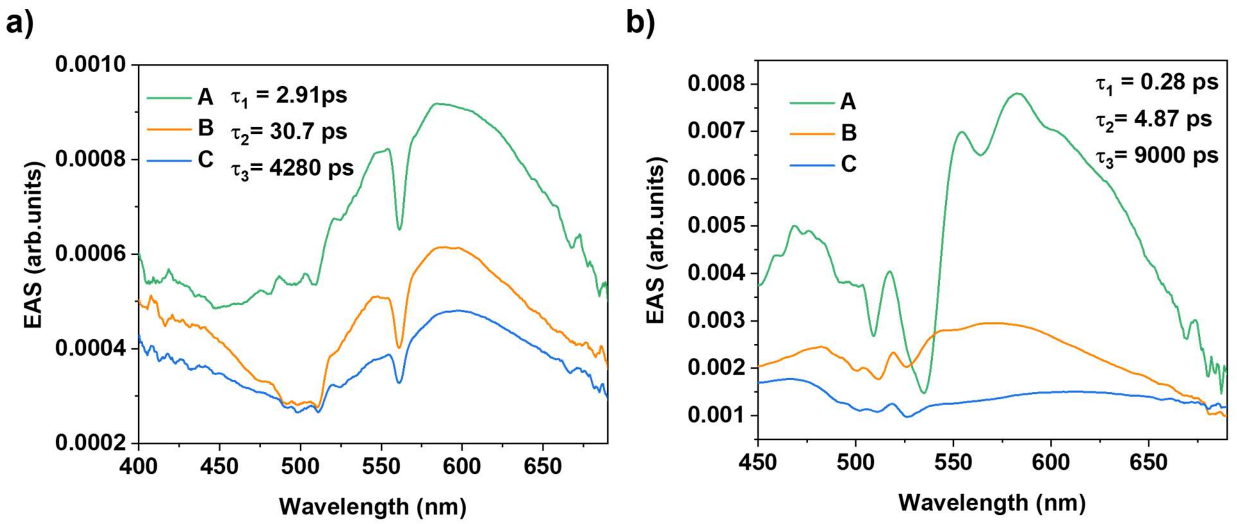


**Figure S19**. Evolution-associated spectra for an A → B → C → ground state sequential decay model of **4** (a) and **8** (b).

**6. The cyclic voltammogram and Differential pulse voltammetry**

All cyclic redox potential curves were measured in dry and degassed DMF solution of **3**, **4**, **5**, **7**, **8** and **9** with tetrabutylammonium hexafluorophosphate (1 M) as supporting electrolyte, potential E referenced to $Fc/Fc^+$.

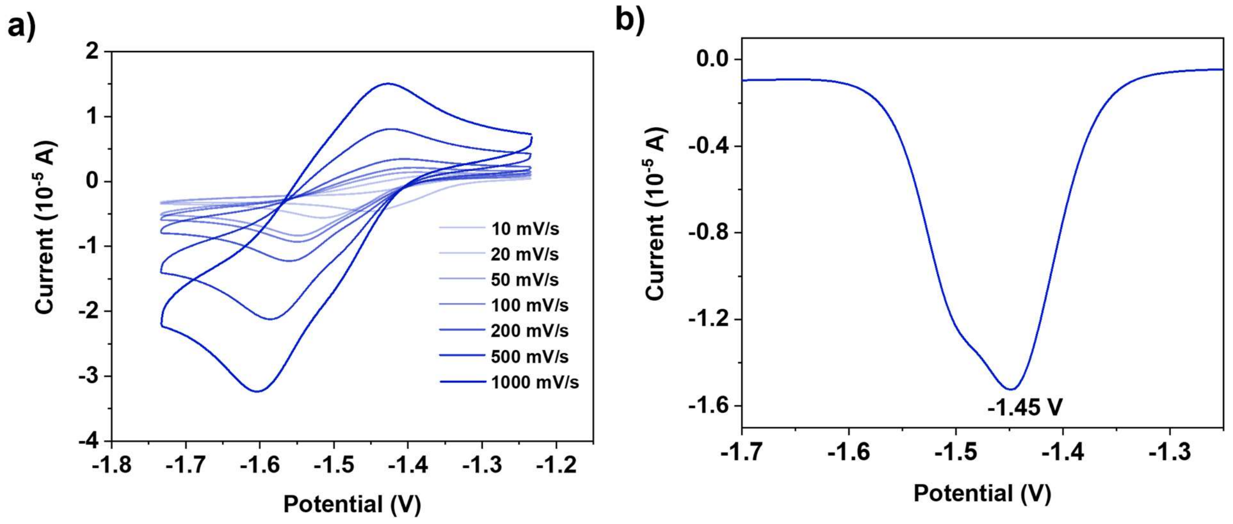


**Figure S20**. (a) The cyclic voltammogram of **3** in DMF solution with tetrabutylammonium hexafluorophosphate (0.1 M) as supporting electrolyte, potential E referenced to $Fc/Fc^+$; (b) Differential pulse voltammetry of **3**, c = $10^{-4}$ M.

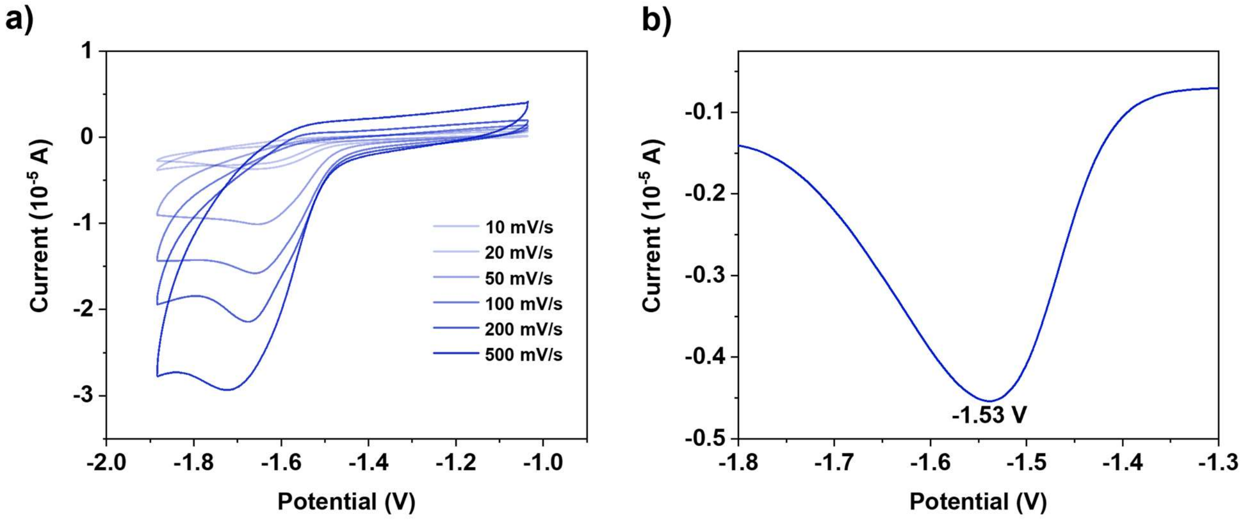


**Figure S21.** (a) The cyclic voltammogram of **4** at different scan rates in DMF solution with tetrabutylammonium hexafluorophosphate (0.1 M) as supporting electrolyte, potential E referenced to $Fc/Fc^+$; (b) Differential pulse voltammetry of **4**, c = $10^{-4}$ M.

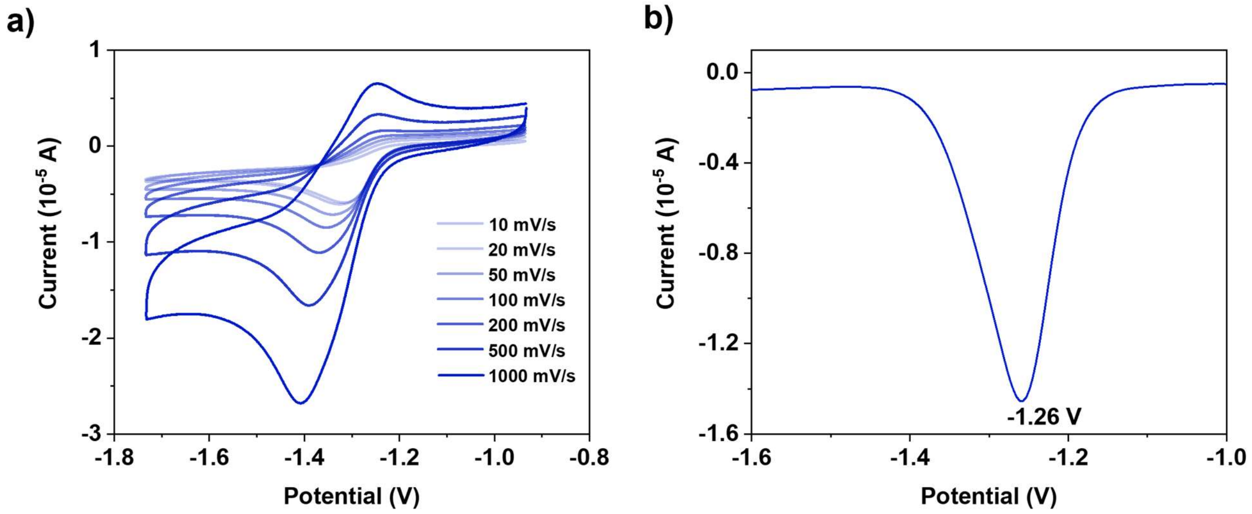


**Figure S22.** (a) The cyclic voltammogram of **5** at different scan rates in DMF solution with tetrabutylammonium hexafluorophosphate (0.1 M) as supporting electrolyte, potential E referenced to $Fc/Fc^{+}$; (b) Differential pulse voltammetry of **5**, c = $10^{-4}$ M.

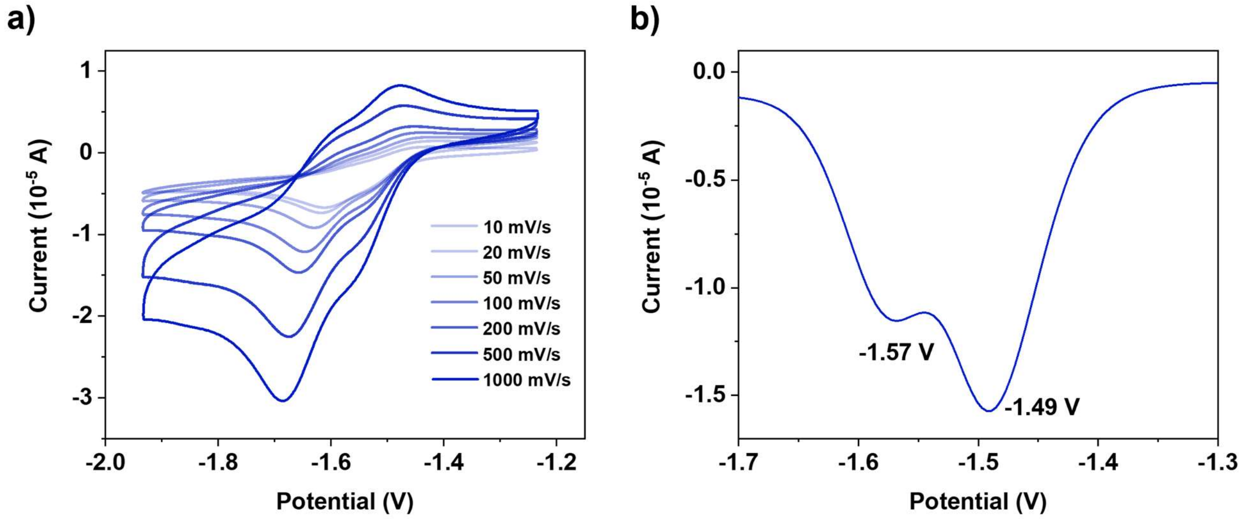


**Figure S23**. (a) The cyclic voltammogram of **7** at different scan rates in DMF solution with tetrabutylammonium hexafluorophosphate (0.1 M) as supporting electrolyte, potential E referenced to $Fc/Fc^{+}$; (b) Differential pulse voltammetry of **7**, c = $10^{-4}$ M.

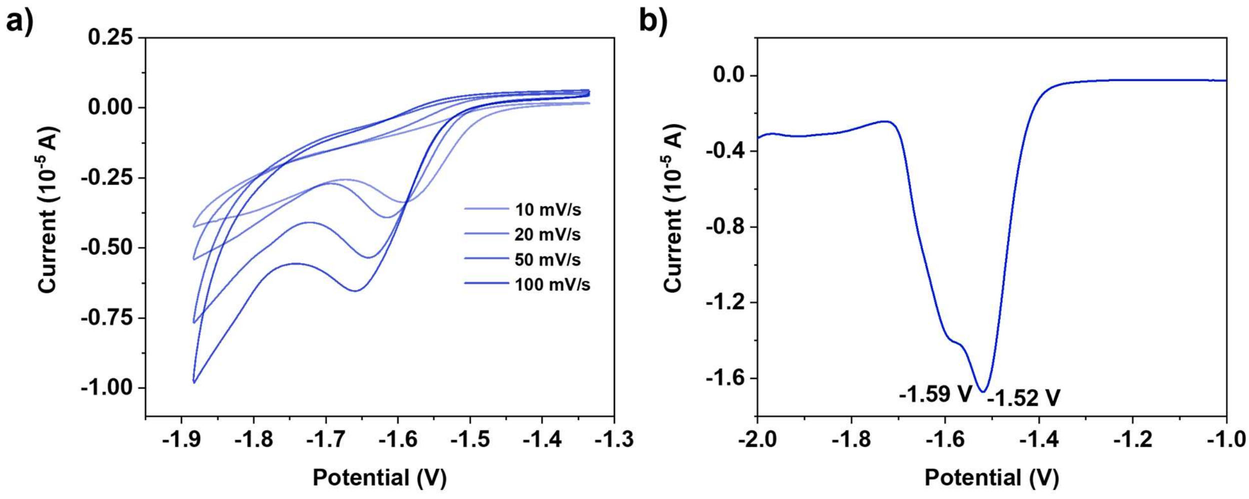


**Figure S24**. (a) The cyclic voltammogram of **8** at different scan rates in DMF solution with tetrabutylammonium hexafluorophosphate (0.1 M) as supporting electrolyte, potential E referenced to $Fc/Fc^{+}$; (b) Differential pulse voltammetry of **8**, c = $10^{-4}$ M.

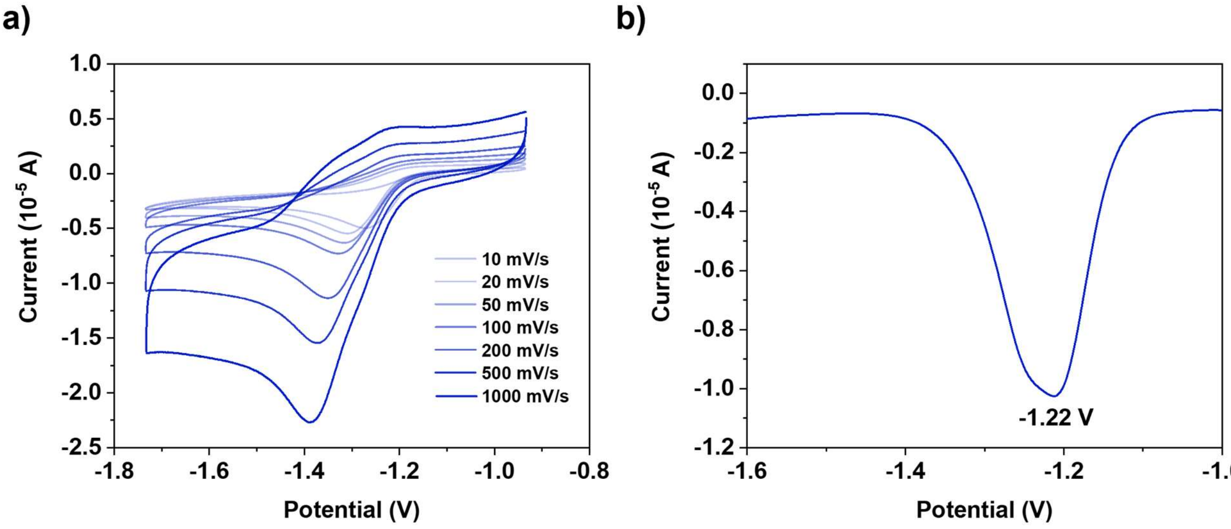


**Figure S25.** (a) The cyclic voltammogram of **9** at different scan rates in DMF solution with tetrabutylammonium hexafluorophosphate (0.1 M) as supporting electrolyte, potential E referenced to $Fc/Fc^{+}$; (b) Differential pulse voltammetry of **9**, c = $10^{-4}$ M.

## 7. Evaluation of HOMO and LUMO energy

The HOMO and LUMO energy levels of **3** and **5** were determined by CV experiments.[1] The LUMO energy level can be calculated according to the following formula:

$$E\ (\mathrm{LUMO}) = -[E(\mathrm{onset, red,1}) + 4.8 - E(\mathrm{Fc/Fc^+})]\ \mathrm{eV}$$

where $E$ (onset, red) is the onset reduction potential vs. $Fc/Fc^+$ electrode.

The HOMO/LUMO energy gap (ΔE) can be evaluated from the onset absorption wavelength ($\lambda_{onset}$) of **3**, **4**, **5**, **7**, **8 and 9** in the UV/vis spectra according to the following formula:

$$\Delta E\ (\mathrm{HOMO/LUMO}) = (1240/\lambda_{onset})\ \mathrm{eV}$$

The HOMO energy level can be determined:

$$E\ (\mathrm{HOMO}) = E\ (\mathrm{LUMO}) - \Delta E\ (\mathrm{HOMO/LUMO})\ \mathrm{eV}$$

**Table S8**. The experiment data and calculation results for **3**, **4**, **5**, **7**, **8** and **9**.

| | $E_{red}$ [V] | $E_{LUMO}$ [eV] | $\lambda_{onset}$ [nm] | $\Delta E$[a] [eV] (HOMO/LUMO) | $E_{HOMO}$ [eV] | $\Delta E$[b] [eV] (HOMO/LUMO) (calcd) | $E_{LUMO}$ [eV] calcd | $E_{HOMO}$ [eV] calcd |
|---|---|---|---|---|---|---|---|---|
| **3** | -1.55 | -3.98 | 355 | 3.49 | -7.47 | 3.76 | -3.11 | -6.87 |
| **4** | -1.66 | -3.87 | 367 | 3.38 | -7.25 | 3.66 | -3.22 | -6.88 |
| **5** | -1.35 | -4.18 | 388 | 3.20 | -7.38 | 3.61 | -3.28 | -6.89 |
| **7** | -1.64 | -3.89 | 363 | 3.41 | -7.30 | 3.26 | -3.14 | -6.40 |
| **8** | -1.67 | -3.86 | 375 | 3.31 | -7.17 | 3.23 | -3.19 | -6.42 |
| **9** | -1.33 | -4.20 | 390 | 3.18 | -7.38 | 3.17 | -3.24 | -6.41 |

[a] Energy gap values were calculated from the absorption spectra in DMF. [b] Theoretical calculations were carried out by using the GAUSSIAN09 suite of programs.

## 8. Evaluation of electron-transfer constant $k_{ET}$

The electron-transfer constants $k_{ET}$ were determined using the Nicholson method according to our previous work.[2]

$$i_p = 2.69 \times 10^5 A D_0^{1/2} v^{1/2} c^* = R v^{1/2}$$

where electrode radius r = 0.15 cm, electrode area $A = \pi r^2 = 0.07065\ cm^2$, concentration $c^* = 1 \times 10^{-6}$ mol/cm$^3$.

When scan rate v = 0.1 V/s,

$$k_{ET} = \Psi(\pi D_0 Fv/RT)^{1/2} = 182\Psi R$$

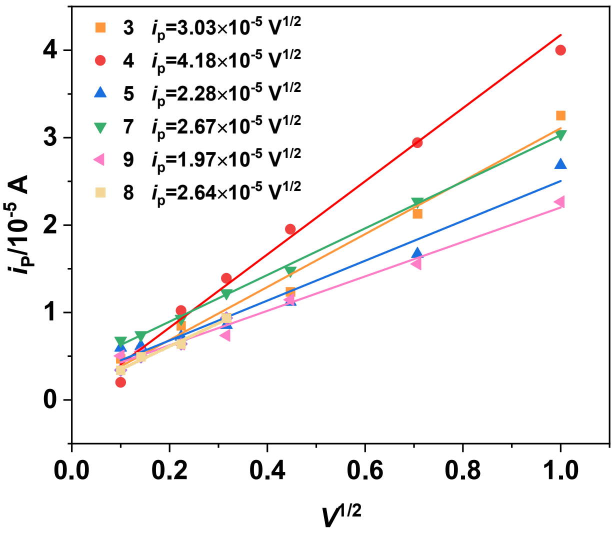


**Figure S26**. Peak current and scan rate diagrams of **3**, **4**, **5**, **7**, **8** and **9**.

**Table S9**. The electron-transfer constants $k_{ET}$ of **3-9**.

| | Eoxi | *ΔEp₁* [mV] [b] | $R_1$ [c] | $\Psi_1$ [d] | $k_{ET1}$ [e] |
|---|---|---|---|---|---|
| **3** | -1.548 | 156 | $3.03 \times 10^{-5}$ | 0.18 | $9.93 \times 10^{-4}$ |
| **4** | -1.591 | 116 | $4.18 \times 10^{-5}$ | 0.40 | $3.04 \times 10^{-4}$ |
| **5** | -1.355 | 142 | $2.28 \times 10^{-5}$ | 0.23 | $9.54 \times 10^{-4}$ |
| **7** | -1.644 | 193 | $2.67 \times 10^{-5}$ | 0.09 | $4.37 \times 10^{-4}$ |
| **8** | -1.670 | 136 | $2.64 \times 10^{-5}$ | 0.26 | $1.25 \times 10^{-3}$ |
| **9** | -1.325 | 154 | $1.97 \times 10^{-5}$ | 0.19 | $6.81 \times 10^{-4}$ |

[a] Reduction potentials measured by cyclic voltammetry.

[b] ΔEp was calculated from CV.

[c] Slope of $i_p \sim v^{1/2}$ in Figure S16.

[d] $\Psi = (-0.6288+0.0021\Delta Ep)/(1-0.017\Delta Ep)$.

[e] electron-transfer constant $k_{ET}$ was evaluated according to Nicholson's formula.

**9. Computed UV/vis spectra**

**Table S10.** Calculated ($\lambda_{TD\text{-}DFT}$) wavelengths (nm) of **3**. Molecular orbitals (MOs) involved in the main electronic transition, f corresponds to the oscillator strength.

| $\lambda_{TD\text{-}DFT}$ | MOs | Oscillator, Strength, f | |
|---|---|---|---|
| 373.36 | HOMO-1 -> LUMO | 0.04833 | 99.2% |
| 304.58 | HOMO-4 -> LUMO | 0.0782 | 6.0% |
| | HOMO-3 -> LUMO | | 75.9% |
| | HOMO-2 -> LUMO+1 | | 16.7% |
| 297.20 | HOMO-4 -> LUMO | 0.1859 | 14.7% |
| | HOMO-3 -> LUMO | | 7.4% |
| | HOMO-2 -> LUMO+1 | | 72.9% |
| 293.88 | HOMO-4 -> LUMO | 0.2394 | 77.3% |
| | HOMO-3 -> LUMO | | 13.2% |
| | HOMO-2 -> LUMO+1 | | 7.1% |

**Table S11.** Calculated ($\lambda_{TD\text{-}DFT}$) wavelengths (nm) of **4**. Molecular orbitals (MOs) involved in the main electronic transition, f corresponds to the oscillator strength.

| $\lambda_{TD\text{-}DFT}$ | MOs | Oscillator, Strength, f | |
|---|---|---|---|
| 383.56 | HOMO -> LUMO | 0.0752 | 14.8% |
| | HOMO -> LUMO+1 | | 56.1% |
| | HOMO -> LUMO+2 | | 28.5% |
| 317.24 | HOMO-9 -> LUMO+1 | 0.3493 | 7.9% |
| | HOMO-9 -> LUMO+2 | | 6.8% |
| | HOMO-4 -> LUMO+2 | | 8.2% |
| | HOMO-3 -> LUMO | | 3.4% |
| | HOMO-3 -> LUMO+1 | | 61.2% |
| 304.52 | HOMO-3 -> LUMO | 0.0999 | 7.3% |
| | HOMO-3 -> LUMO+2 | | 7.1% |
| | HOMO-2 -> LUMO | | 47.7% |
| | HOMO-2 -> LUMO+1 | | 12.4% |
| | HOMO-2 -> LUMO+3 | | 16.6% |
| 251.68 | HOMO-2 -> LUMO+5 | 0.1254 | 5.7% |
| | HOMO-1 -> LUMO+6 | | 79.0% |
| | HOMO-1 -> LUMO+8 | | 3.5% |

**Table S12.** Calculated ($\lambda_{TD\text{-}DFT}$) wavelengths (nm) of **5**. Molecular orbitals (MOs) involved in the main electronic transition, f corresponds to the oscillator strength.

| $\lambda_{TD\text{-}DFT}$ | MOs | Oscillator, Strength, f | |
|---|---|---|---|
| 394.73 | HOMO -> LUMO | 0.0942 | 99.4% |
| 329.03 | HOMO-2 -> LUMO | 0.6207 | 94.9% |
| 311.19 | HOMO-7 -> LUMO | 0.4012 | 6.6% |
| | HOMO-3 -> LUMO+1 | | 89.3% |
| 252.16 | HOMO-2 -> LUMO+2 | 0.1419 | 2.1% |
| | HOMO-1 -> LUMO+4 | | 85.5% |
| | HOMO-1 -> LUMO+6 | | 4.1% |

**Table S13.** Calculated ($\lambda_{TD-DFT}$) wavelengths (nm) of **7**. Molecular orbitals (MOs) involved in the main electronic transition, f corresponds to the oscillator strength.

| $\lambda_{TD-DFT}$ | MOs | Oscillator, Strength, f | |
|---|---|---|---|
| 375.35 | HOMO-1 -> LUMO | 0.0443 | 3.5% |
| | HOMO -> LUMO+1 | | 95.4% |
| 298.41 | HOMO-4 -> LUMO | 0.3784 | 37.0% |
| | HOMO-3 -> LUMO | | 3.4% |
| | HOMO-2 -> LUMO+1 | | 36.3% |
| | HOMO-1 -> LUMO+2 | | 8.2% |
| | HOMO -> LUMO+4 | | 7.9% |
| 272.09 | HOMO-4 -> LUMO+1 | 0.3357 | 2.9% |
| | HOMO-2 -> LUMO+2 | | 2.0% |
| | HOMO-1 -> LUMO+2 | | 32.1% |
| | HOMO-1 -> LUMO+3 | | 2.0% |
| | HOMO-1 -> LUMO+4 | | 13.1% |
| | HOMO-1 -> LUMO+5 | | 6.0% |
| | HOMO -> LUMO+4 | | 4.8% |
| | HOMO -> LUMO+5 | | 32.5% |
| 259.83 | HOMO-1 -> LUMO+2 | 0.3253 | 2.0% |
| | HOMO-1 -> LUMO+4 | | 68.9% |
| | HOMO-1 -> LUMO+5 | | 2.1% |
| | HOMO -> LUMO+4 | | 2.1% |
| | HOMO -> LUMO+5 | | 9.6% |
| | HOMO -> LUMO+6 | | 4.3% |

**Table S14.** Calculated ($\lambda_{TD\text{-}DFT}$) wavelengths (nm) of **8**. Molecular orbitals (MOs) involved in the main electronic transition, f corresponds to the oscillator strength.

| $\lambda_{TD\text{-}DFT}$ | MOs | Oscillator, Strength, f | |
|---|---|---|---|
| 377.34 | HOMO-1 -> LUMO | 0.0602 | 3.2% |
| | HOMO -> LUMO+1 | | 95.3% |
| 304.93 | HOMO-6 -> LUMO | 0.1430 | 12.1% |
| | HOMO-5 -> LUMO | | 2.6% |
| | HOMO-4 -> LUMO | | 6.3% |
| | HOMO-3 -> LUMO | | 62.1% |
| | HOMO-2 -> LUMO+1 | | 8.9% |
| 300.95 | HOMO-6 -> LUMO | 0.4012 | 3.1% |
| | HOMO-5 -> LUMO | | 8.9% |
| | HOMO-4 -> LUMO | | 3.4% |
| | HOMO-3 -> LUMO | | 15.7% |
| | HOMO-2 -> LUMO+1 | | 55.9% |
| | HOMO-1 -> LUMO+5 | | 2.6% |
| | HOMO -> LUMO+4 | | 3.1% |
| 273.46 | HOMO-3 -> LUMO+1 | 0.2163 | 2.9% |
| | HOMO-1 -> LUMO+2 | | 2.4% |
| | HOMO-1 -> LUMO+3 | | 8.8% |
| | HOMO-1 -> LUMO+5 | | 61.5% |
| | HOMO-1 -> LUMO+7 | | 3.7% |
| | HOMO -> LUMO+6 | | 6.8% |
| | HOMO -> LUMO+7 | | 3.7% |

**Table S15.** Calculated ($\lambda_{TD-DFT}$) wavelengths (nm) of **9**. Molecular orbitals (MOs) involved in the main electronic transition, f corresponds to the oscillator strength.

| $\lambda_{TD-DFT}$ | MOs | Oscillator, Strength, f | |
|---|---|---|---|
| 389.76 | HOMO-1 -> LUMO | 0.0412 | 4.2% |
| | HOMO -> LUMO+1 | | 94.7% |
| 317.27 | HOMO-4 -> LUMO | 0.4723 | 23.2% |
| | HOMO-3 -> LUMO | | 68.5% |
| | HOMO-2 -> LUMO+1 | | 3.6% |
| 310.66 | HOMO-4 -> LUMO | 0.3457 | 6.6% |
| | HOMO-2 -> LUMO+1 | | 87.0% |
| 274.38 | HOMO-7 -> LUMO+1 | 0.3399 | 8.0% |
| | HOMO-1 -> LUMO+2 | | 34.9% |
| | HOMO-1 -> LUMO+4 | | 13.1% |
| | HOMO-1 -> LUMO+5 | | 3.9% |
| | HOMO -> LUMO+4 | | 3.0% |
| | HOMO -> LUMO+5 | | 27.5% |

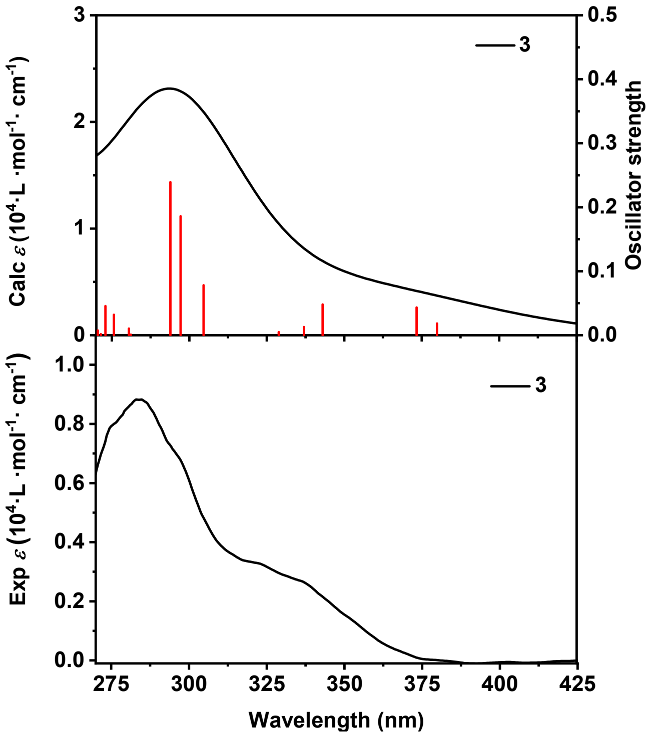


**Figure S27.** Computed UV/vis absorbance spectrum at the TD-B3LYP/6-311G (d, p) level of theory in DMF, and experimental UV/vis spectra in DMF of **3**.

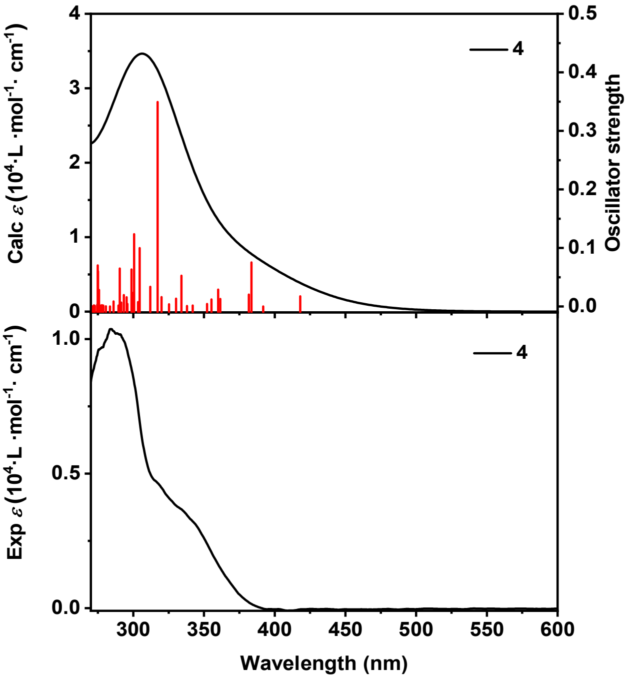


**Figure S28.** Computed UV/vis absorbance spectrum at the TD-B3LYP/6-311G (d, p) level of theory in DMF, and experimental UV/vis spectra in DMF of **4**.

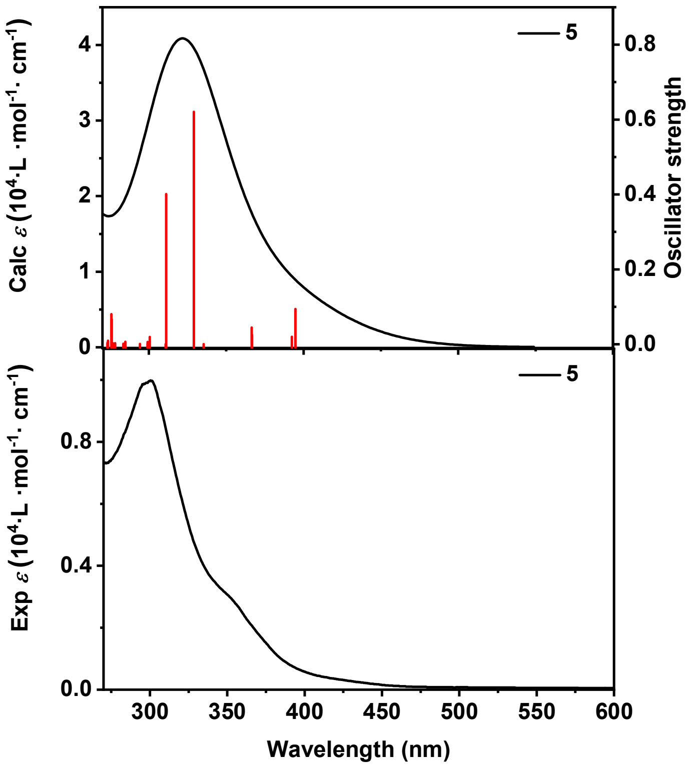


**Figure S29.** Computed UV/vis absorbance spectrum at the TD-B3LYP/6-311G (d, p) level of theory in DMF, and experimental UV/vis spectra in DMF of **5**.

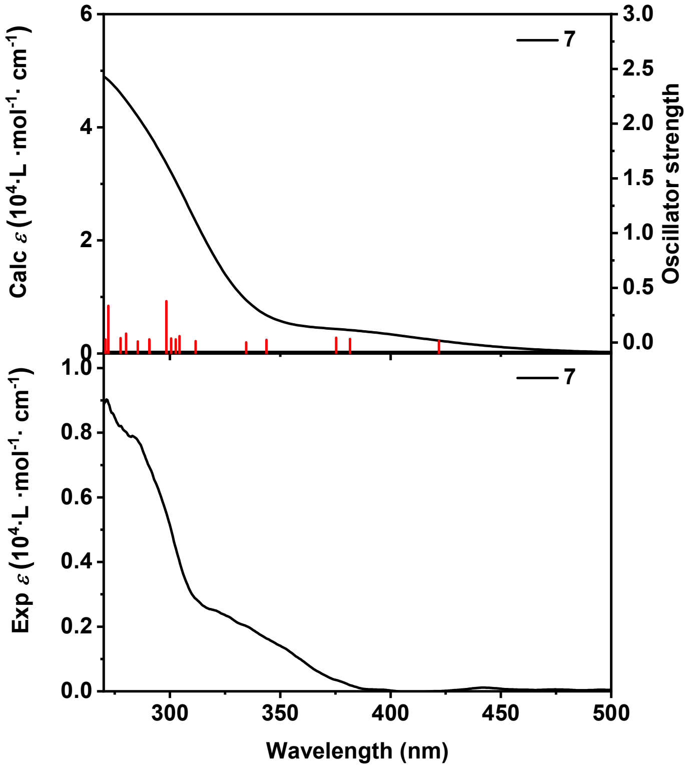


**Figure S30.** Computed UV/vis absorbance spectrum at the TD-B3LYP/6-311G (d, p) level of theory in DMF, and experimental UV/vis spectra in DMF of **7**.

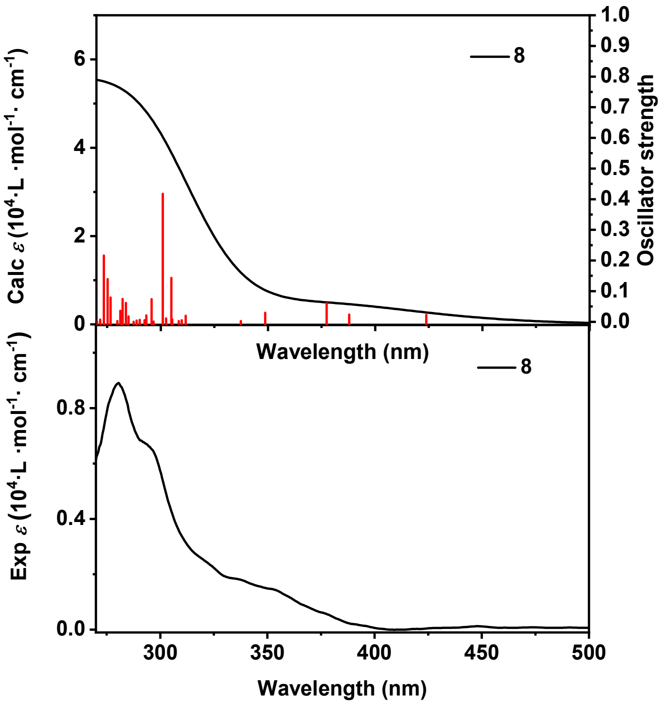


**Figure S31.** Computed UV/vis absorbance spectrum at the TD-B3LYP/6-311G (d, p) level of theory in DMF, and experimental UV/vis spectra in DMF of **8**.

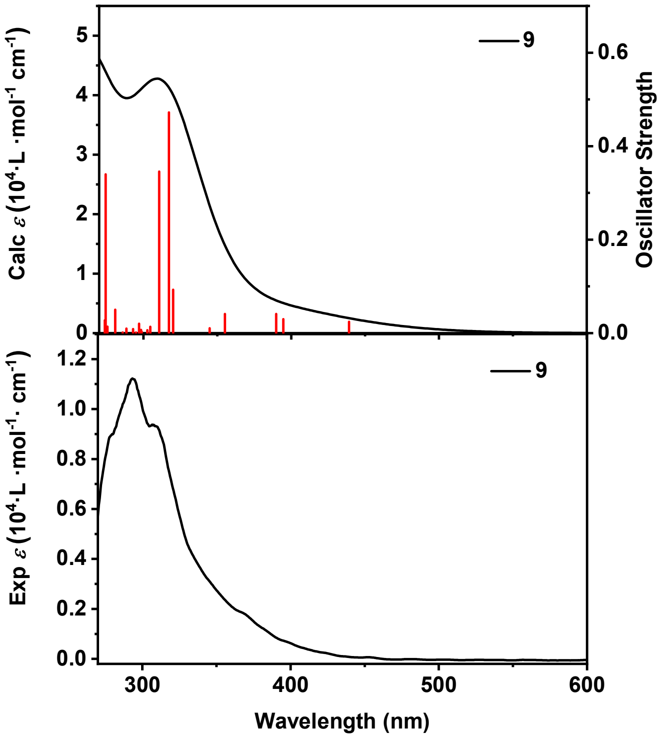


**Figure S32.** Computed UV/vis absorbance spectrum at the TD-B3LYP/6-311G (d, p) level of theory in DMF, and experimental UV/vis spectra in DMF of **9**.

## 10. DFT calculations

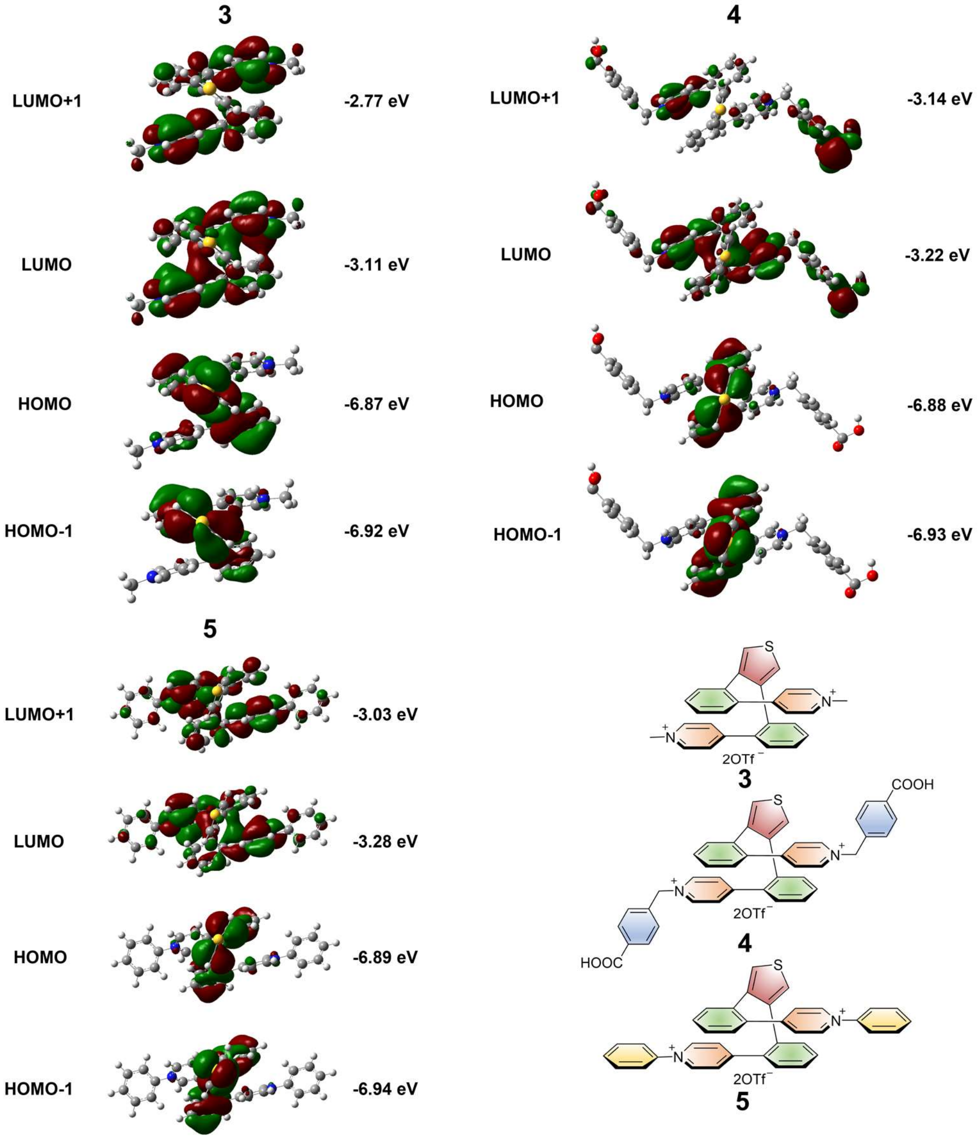


**Figure S33.** The calculated orbitals of **3**, **4** and **5**.

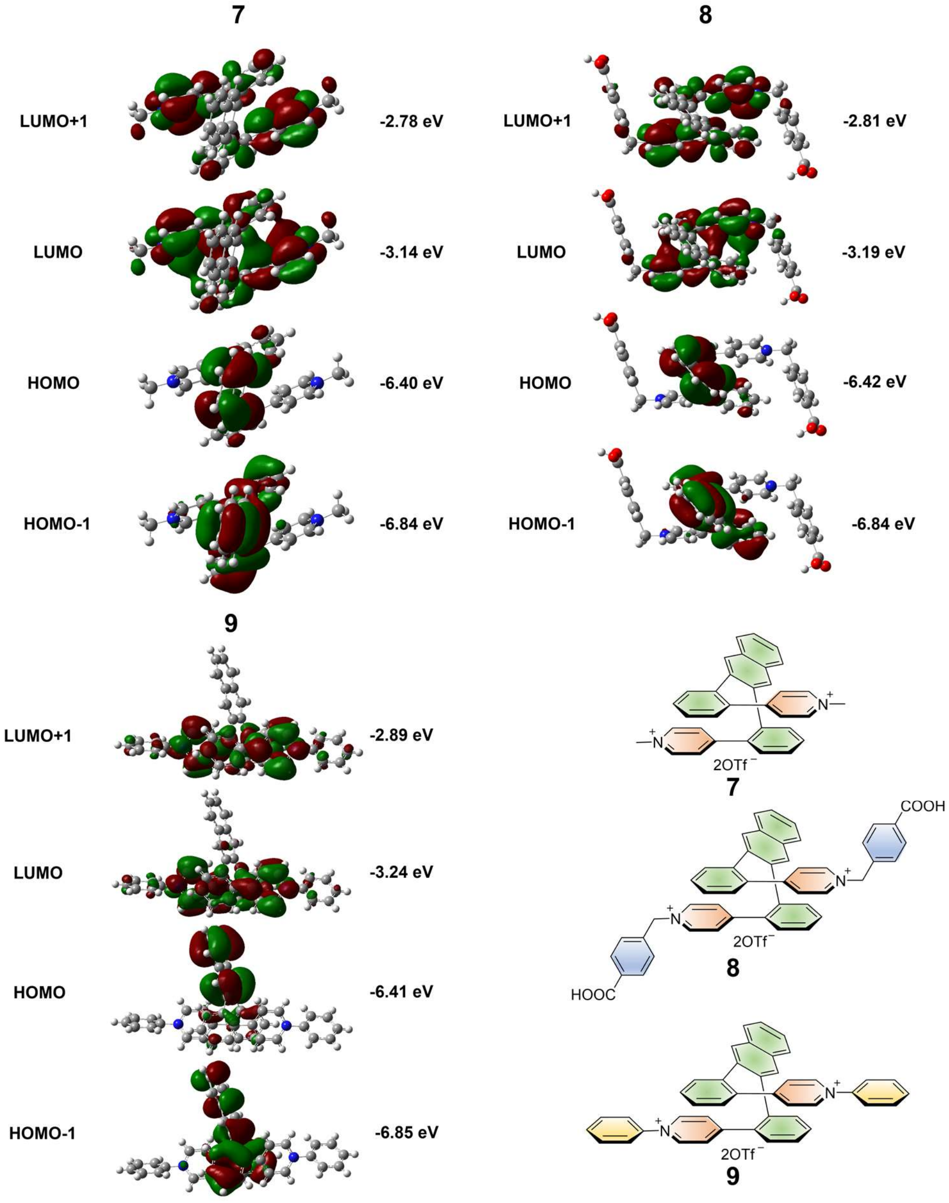


**Figure S34.** The calculated orbitals of **7**, **8** and **9**.

## 11. Electrostatic potential surfaces

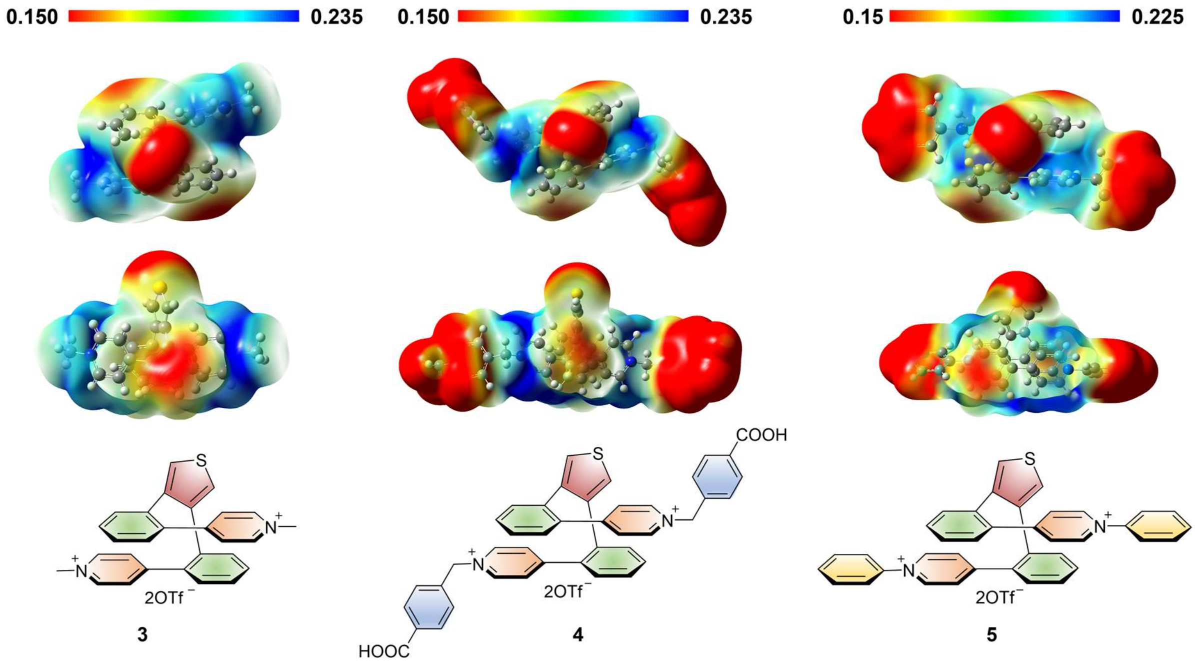


**Figure S35.** The calculated spin density plots of **3**, **4** and **5**.

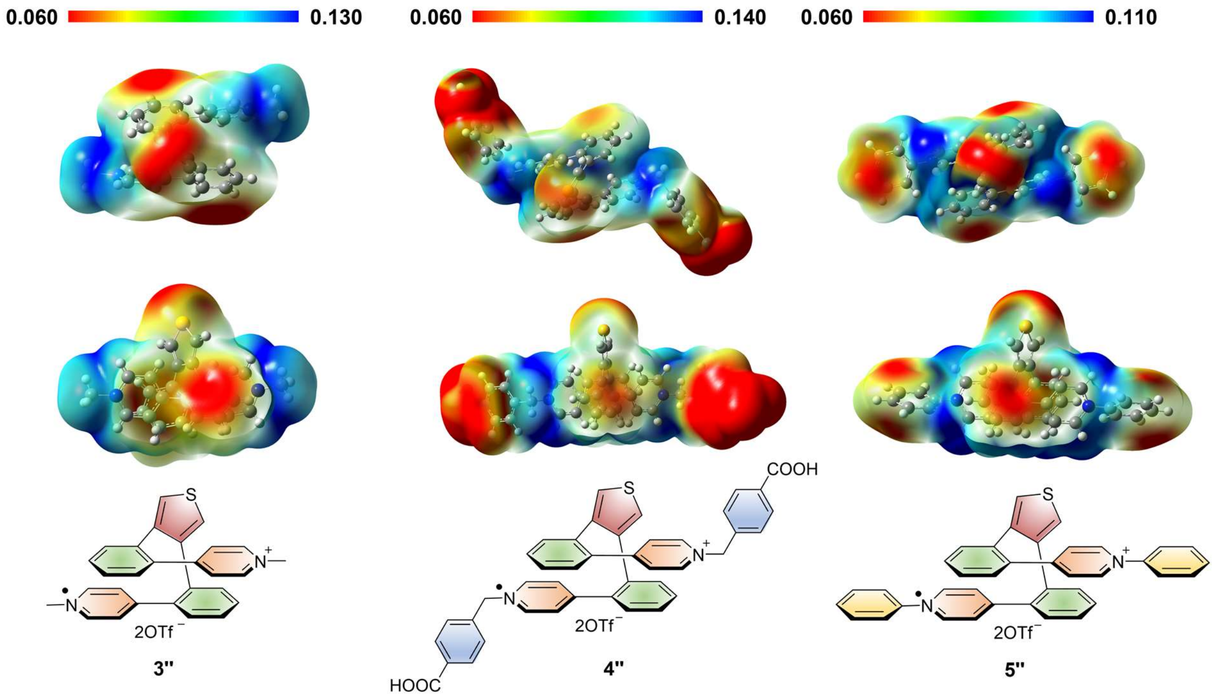


**Figure S36.** The calculated spin density plots of radical species **3''**, **4''** and **5''** in the triplet ground state.

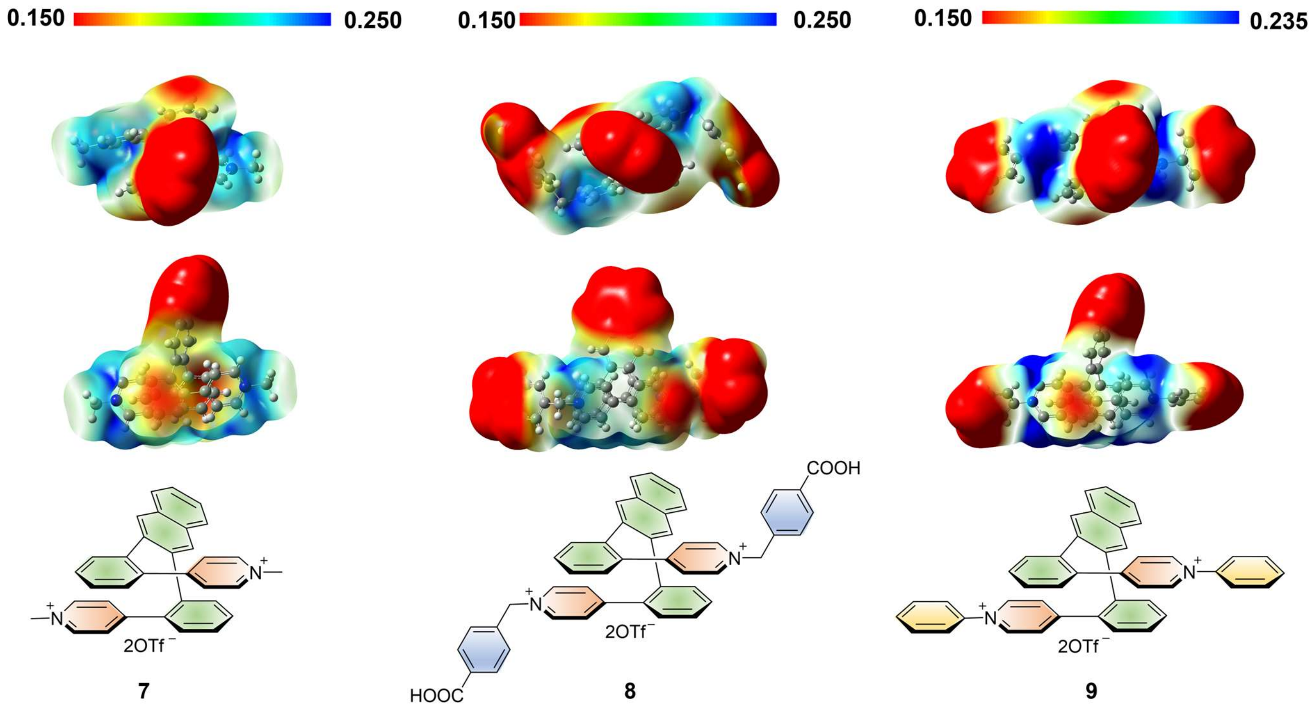


**Figure S37.** The calculated spin density plots of **7**, **8** and **9**

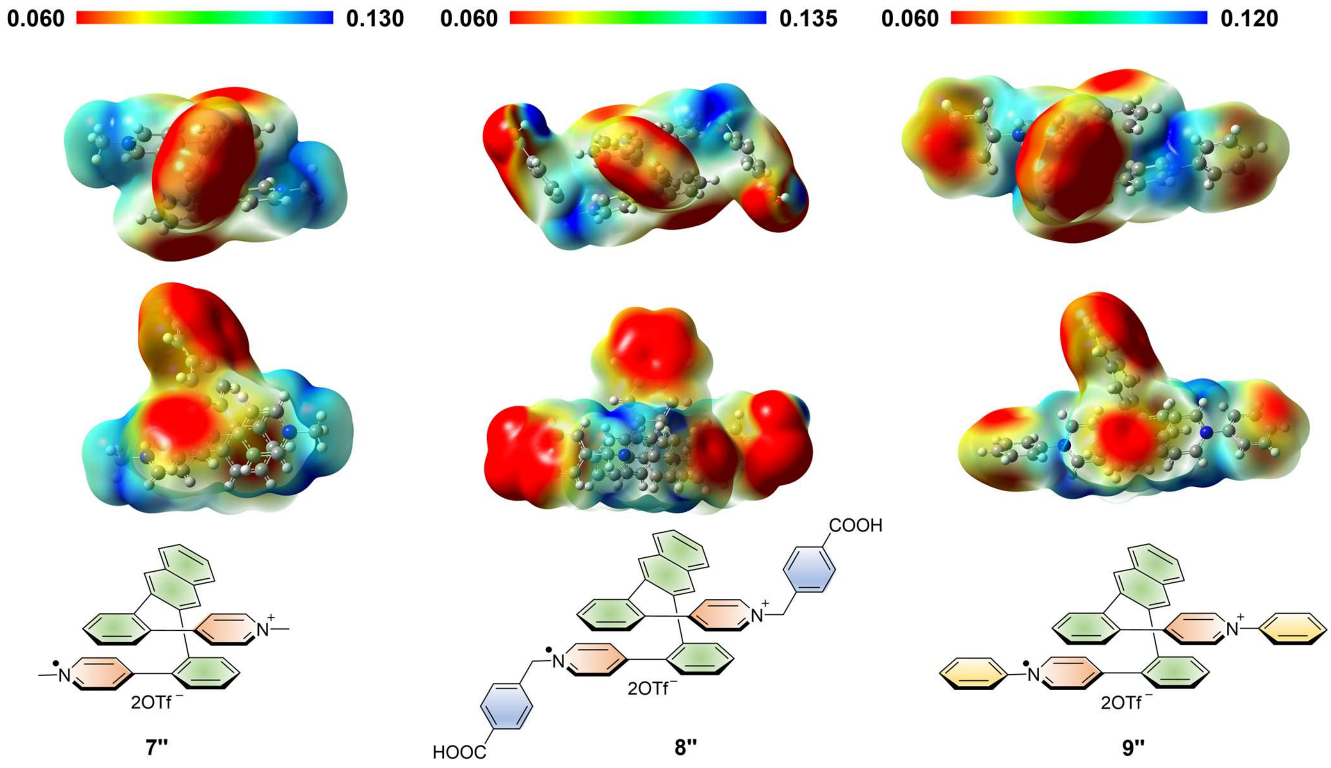


**Figure S38.** The calculated spin density plots of radical species **7''**, **8''** and **9''** in the triplet ground state.

**12. UV/vis spectra of radical species in DMF**

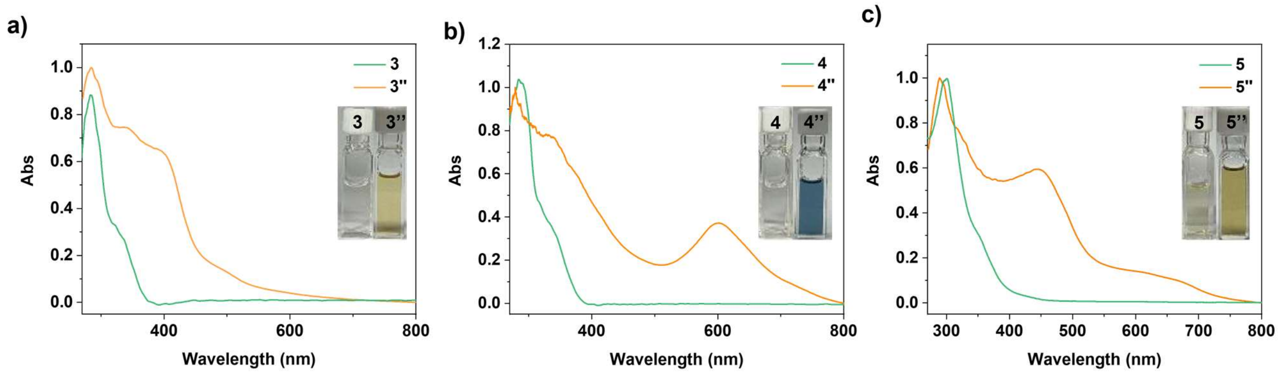


**Figure S39.** (a) UV/vis spectra of **3** by chemical reduction with Na (**3''**), photographs are shown as the inset; (b) UV/vis spectra of **4** by chemical reduction with Na (**4''**), photographs are shown as the inset; (c) UV/vis spectra of **5** by chemical reduction with Na (**5''**), photographs are shown as the inset.

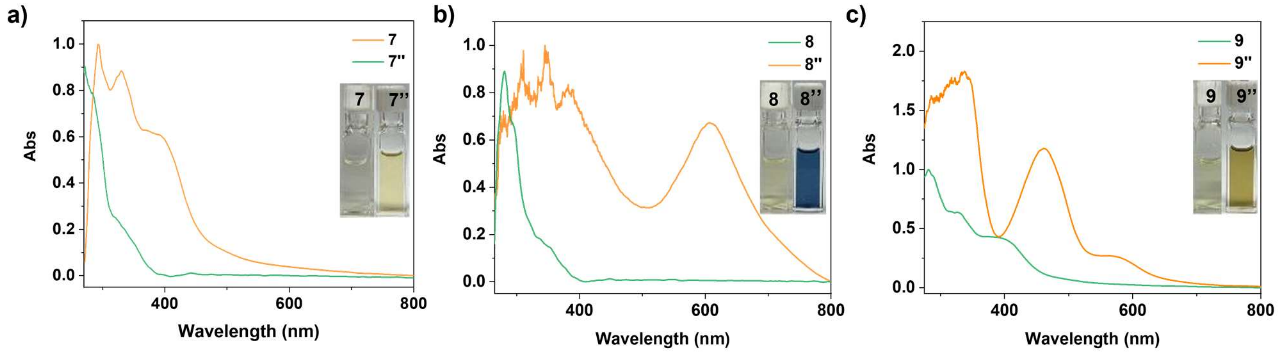


**Figure S40.** UV/vis spectra of (a) **7''**, (b) **8''** and (**9''**) by chemical reduction with Na, photographs are shown as the inset.

## 13. EPR spectrum

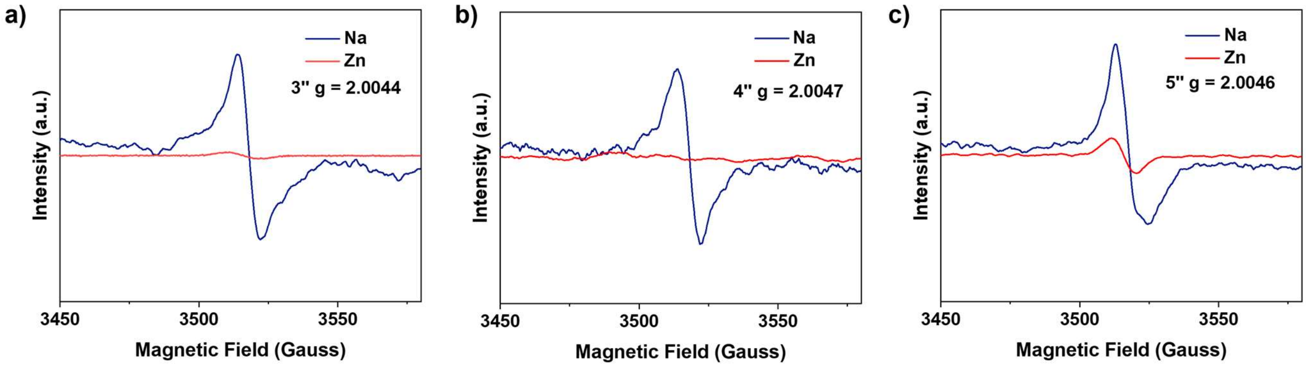


**Figure S41.** EPR spectra of **3**, **4**, and **5** by adding sodium and zinc powder at room temperature (c = $10^{-4}$ M) in DMF.

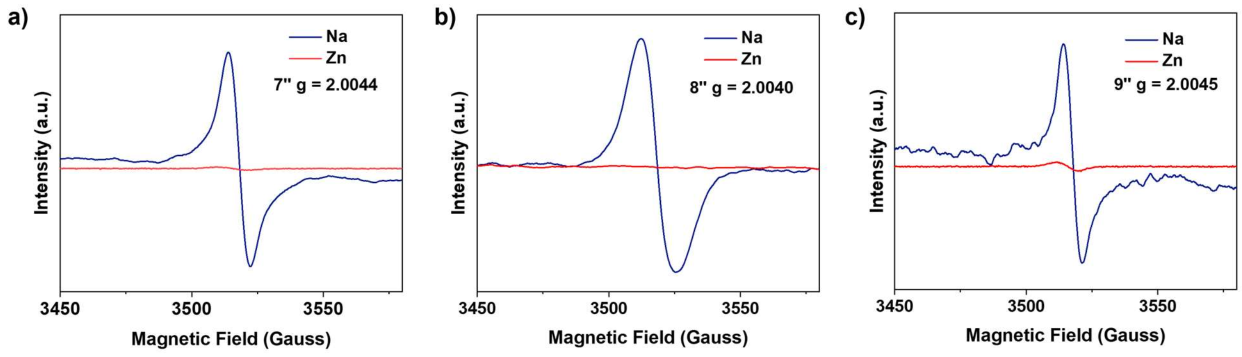


**Figure S42.** EPR spectra of **7**, **8** and **9** by adding sodium powder at room temperature (c = $10^{-4}$ M) in DMF.

## 14. Photocatalytic oxidative coupling of amines into imines

Three parallel experiments were carried out under the same condition at the same time to ensure the accuracy of the experiment, then take the middle yield value to make a contrast when three data have small errors. In the typical catalytic process, 0.2 mmol benzylamine as substrate, 1 μmol photocatalyst, and 2 ml DMSO as solvent were added in a 20 ml Pyrex bottle with a rubber stopper. Adding $O_2$ to the reaction system for 15 min and the reaction mixture was stirred at room temperature for 24 h in a photoreactor purchased in WATTCAS (PCS230850), the lamp with a peak wavelength of 457 nm, dominant wavelength of 476 nm and spectral half width of 28.2 nm as the light source. After the reaction, the solvent was removed by evaporation under reduced pressure. Then 0.033 mmol 1,3,5-trimethoxybenzene was added as an internal standard, and the yield of imine was determined by $^1H$ NMR.

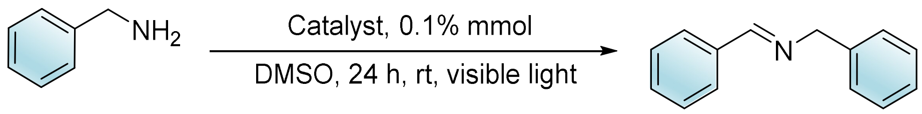


**Table S16**. Screening experiment on photocatalytic oxidative coupling of benzylamine.

| Entry | Catalyst | Atmosphere | Light | Solvent | Yield (%) |
|---|---|---|---|---|---|
| 1 | **9** | $O_2$ | + | $CH_3CN$ | 57 |
| 2 | **9** | $O_2$ | + | $CHCl_3$ | 72 |
| 3 | **9** | $O_2$ | + | $CH_3OH$ | 85 |
| 4 | **9** | $O_2$ | + | DMSO | 94 |
| 5 | **-** | $O_2$ | + | DMSO | - |
| 6 | **9** | Ar | + | DMSO | 12 |
| 7 | **9** | Air | + | DMSO | 85 |
| 8 | **9** | $O_2$ | - | DMSO | - |
| 9 | **3** | $O_2$ | + | DMSO | 87 |
| 10 | **4** | $O_2$ | + | DMSO | 43 |
| 11 | **5** | $O_2$ | + | DMSO | 89 |
| 12 | **7** | $O_2$ | + | DMSO | 90 |
| 13 | **8** | $O_2$ | + | DMSO | 88 |
| 14 | **TSC-MV$^{2+}$** | $O_2$ | + | DMSO | 18 |
| 15 | **TSC-pTAV$^{2+}$** | $O_2$ | + | DMSO | 16 |
| 16 | **TSC-PhV$^{2+}$** | $O_2$ | + | DMSO | 21 |
| 17 | **PhV$^{2+}$** | $O_2$ | + | DMSO | 24 |

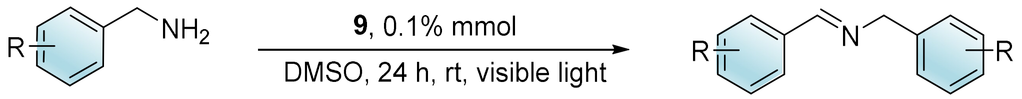


**Table S17**. Photocatalytic oxidative coupling of primary amines by **9**.

| | Amines | Products | Yield (%) |
|---|---|---|---|
| **1** | 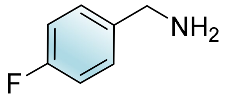 | 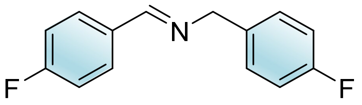 | 88 |
| **2** | 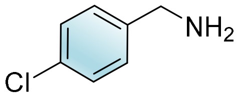 | 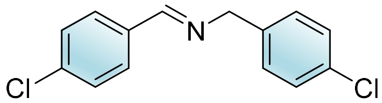 | 80 |
| **3** | 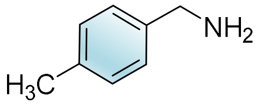 | 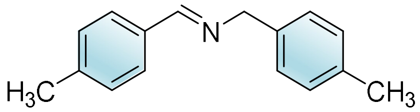 | 99 |
| **4** | 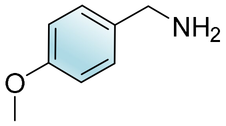 | 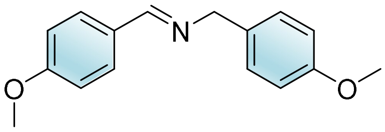 | 88 |
| **5** | 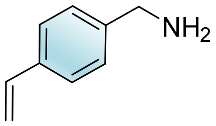 | 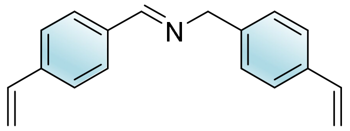 | 61 |
| **6** | 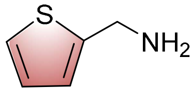 | 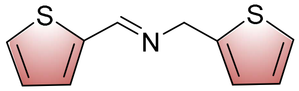 | 80 |
| **7** | 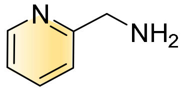 | 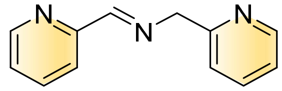 | 76 |
| **8** | 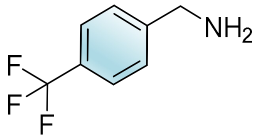 | 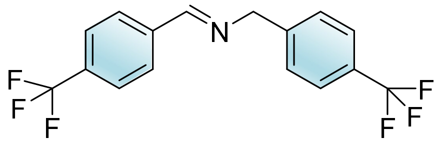 | 79 |

## 15. Hydrogen generation under xenon lamp

Photocatalytic $H_2$ production experiments were performed under Ar using the composite of **4**/**8** and $C_3N_4$ as a photocatalyst, TEOA as the sacrificial donor, platinum nanoparticles as the catalyst for proton reduction and the Xenon lamp (> 400 nm) with light power of 100 mW as the light source.

**Preparation of g-$C_3N_4$:** The g-$C_3N_4$ sample was fabricated by previous reports. Placing urea (10.0 g) in an alumina crucible with a lid, and then place it in a muffle furnace under air atmosphere to calcinate at 550°C for 4h at a heating rate of 5°C min, and then naturally cool to room temperature to obtain a yellow solid powder.

**Preparation of composite catalyst:** Putting 100 mg g-$C_3N_4$, different content of **4/8**, 2.65 mg potassium hexachloroplatinate, and 5 ml methanol in a 20ml Pyrex bottle with a rubber stopper. After that, it was irradiated under the Xenon lamp (> 400 nm) with a light power of 100 mW, and then dry in a vacuum oven for further use.

5 mL TEOA aqueous solution (10 vt%) was added to 20 mL pyrex bottle with 2.5 mL composite photocatalyst. The 20 mL Pyrex bottle was bubbled with Ar gas for 30 min, after that it was irradiated under the Xenon lamp (> 400 nm) with a light power of 100 mW. After the reaction, 3 mL of $CH_4$ was injected into the bottle and mixed uniformly. Then take 200 µL into the gas chromatography (GC). The production of the hydrogen was calculated according to the $H_2$ normalized curve in our previous work.

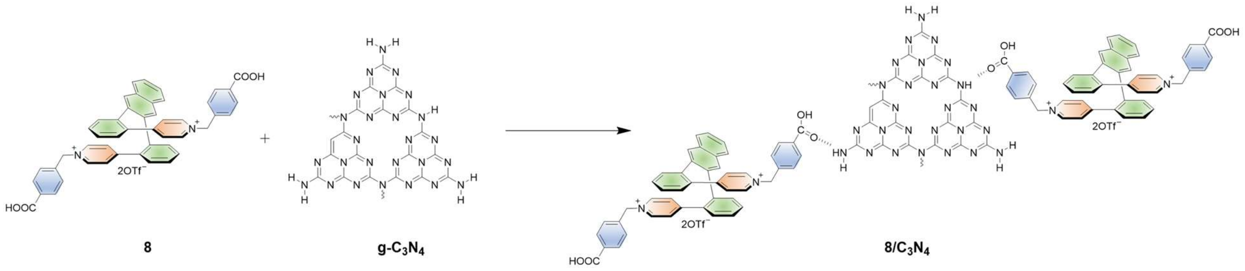


**Figure S43**. The hydrogen bonds between g-$C_3N_4$ and **8**.

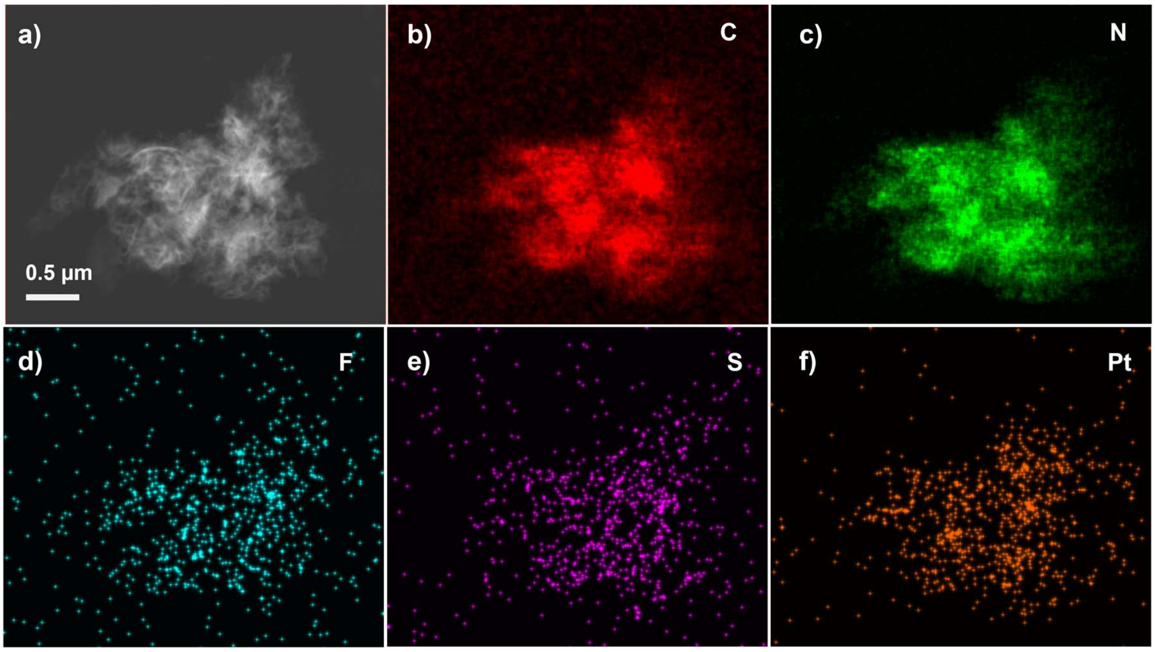


**Figure S44.** TEM images of g-$C_3N_4$/0.1% **8**/1%Pt.

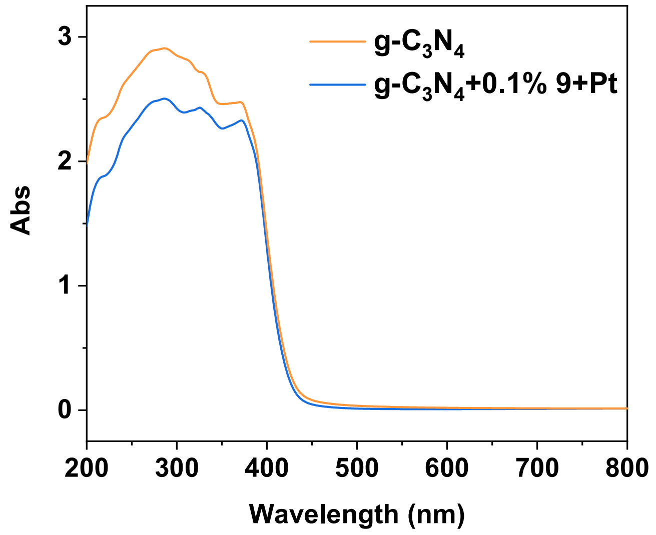


**Figure S45.** Diffuse reflectance UV/vis absorption spectra of g-$C_3N_4$/0.1% /1%Pt, $C_3N_4$ and **8**.

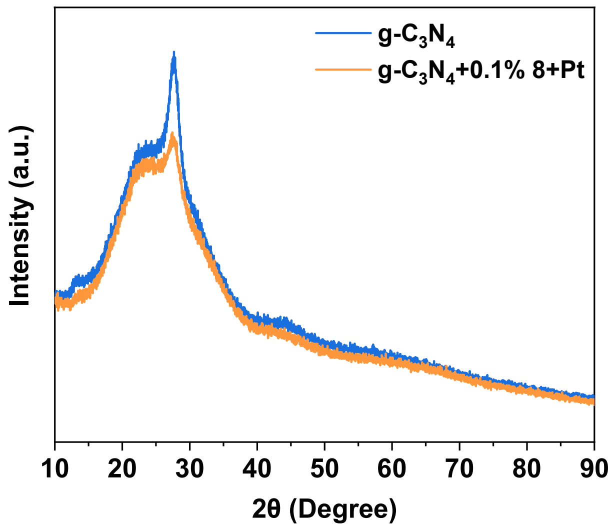


**Figure S46.** XRD patterns of composites of g-$C_3N_4$/0.1% **8**/1%Pt, and g-$C_3N_4$.

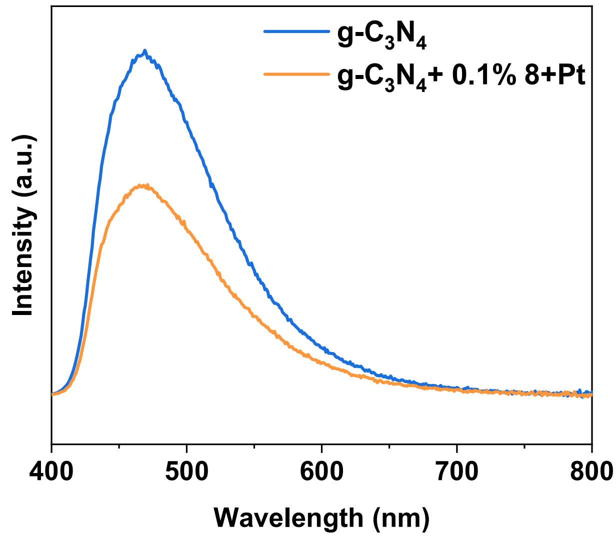


**Figure S47.** Steady-state photoluminescence spectra of the samples (the excitation wavelength is 400 nm).

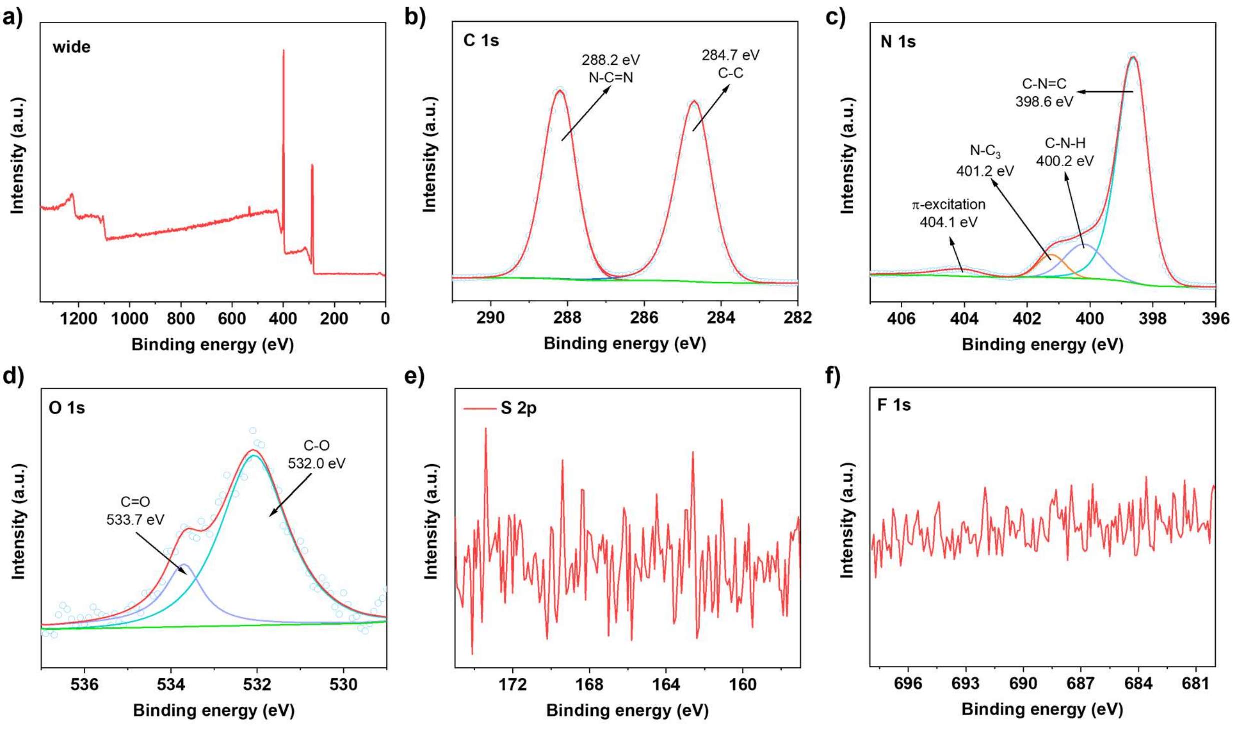


**Figure S48.** (a) XPS spectra and High-resolution (b) C 1s, (c) N 1s, (d) O 1s, (e) S 2p and (f) F 1s XPS spectra of g-$C_3N_4$.

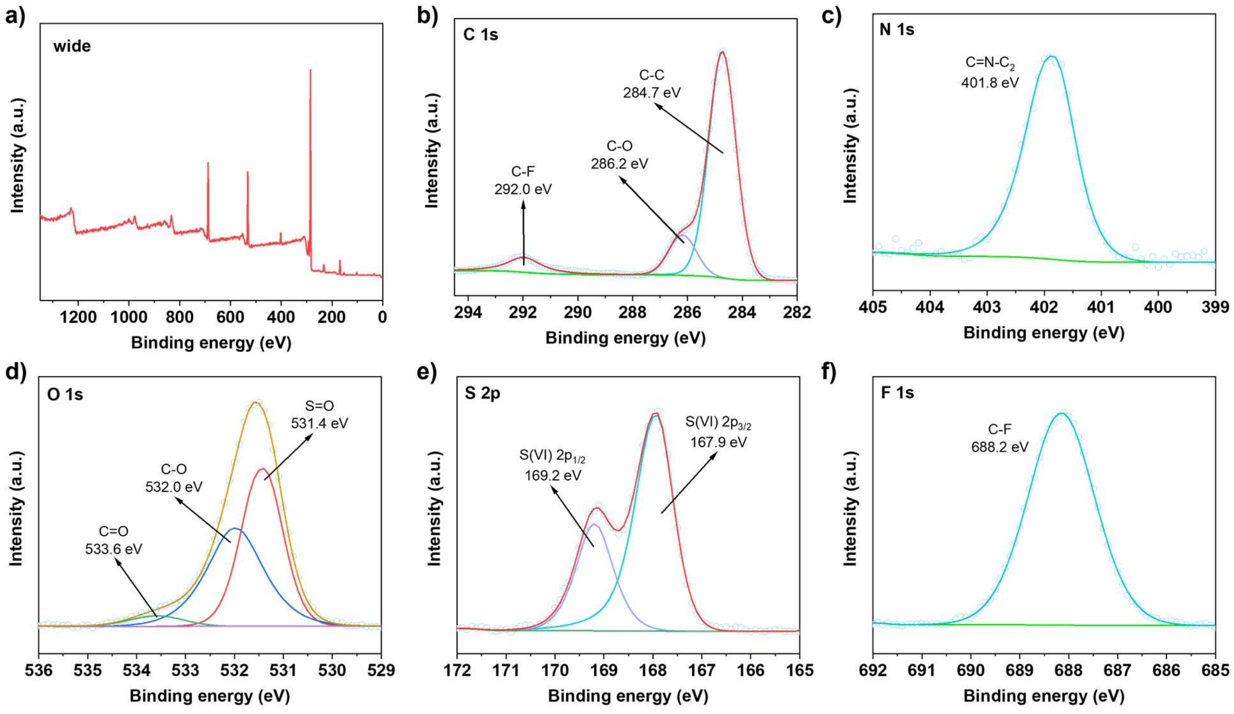


**Figure S49**. (a) XPS spectra and High-resolution (b) C 1s, (c) N 1s, (d) O 1s, (e) S 2p and (f) F 1s of **8**.

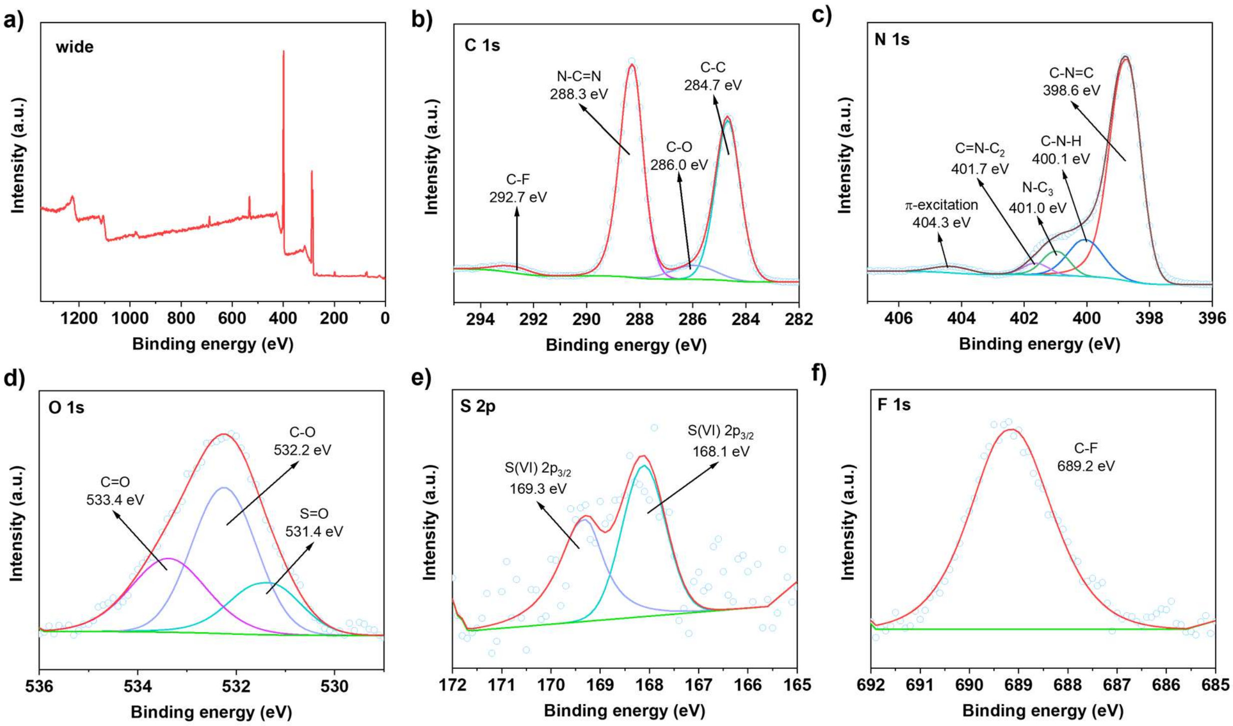


**Figure S50.** (a) XPS spectra and High-resolution (b) C 1s, (c) N 1s, (d) O 1s, (e) S 2p and (f) F 1s of g-$C_3N_4$/0.1% **8**/1%Pt.

**Apparent quantum yield (AQY) calculation:**

Considering that the reduction from $H^+$ to $H_2$ is a two-electron process, the AQY of the process was calculated using the equation (1). For the maximum absorption of g-$C_3N_4$ is 420 nm in visible region ($\lambda$ = 420 nm). E is measured 100 mW. The number of incident photons is calculated to be $2.12\times10^{17}$ $s^{-1}$. The $H_2$ molecules generated in 4 hours under Xenon light was 40.12 μmol. The number of evolved $H_2$ molecules is calculated to be $1.13\times10^{16}$ $s^{-1}$. The AQY of **8** is 1.59%.

$$AQY(\mathbf{8}) = \frac{\text{Number of reacted electrons}}{\text{Number of incident photons}} \times 100\% \qquad (1)$$

$$= \frac{\text{Number of evolved } \mathrm{H_2} \text{ molecules } \times 2}{\text{Number of incident photons}} \times 100\%$$

$$= \frac{2 \times \mathrm{n} \times \mathrm{NA}/\,\mathrm{t}}{\lambda \times \mathrm{E}/\,(\mathrm{h} \times \mathrm{c})} \times 100\%$$

$$= \frac{2 \times 40.12 \times 10^{-6} \times 6.02 \times 10^{23}/4\times3600}{420 \times 10^{-9} \times 100 \times 10^{-3}/\,(6.63 \times 10^{-34} \times 3 \times 10^{8})} \times 100\%$$

$$= \frac{0.44\times10^{16}\ \mathrm{s}^{-1}}{2.11\times10^{17}\ \mathrm{s}^{-1}} \times 100\%$$

$$= 1.59\%$$

## 16. $^{1}$H, $^{13}$C, $^{19}$F and 2D COSY NMR spectra and high resolution mass spectrum (HRMS)

$^{1}$H NMR spectrum (400 MHz, $CD_2Cl_2$) of **2**.

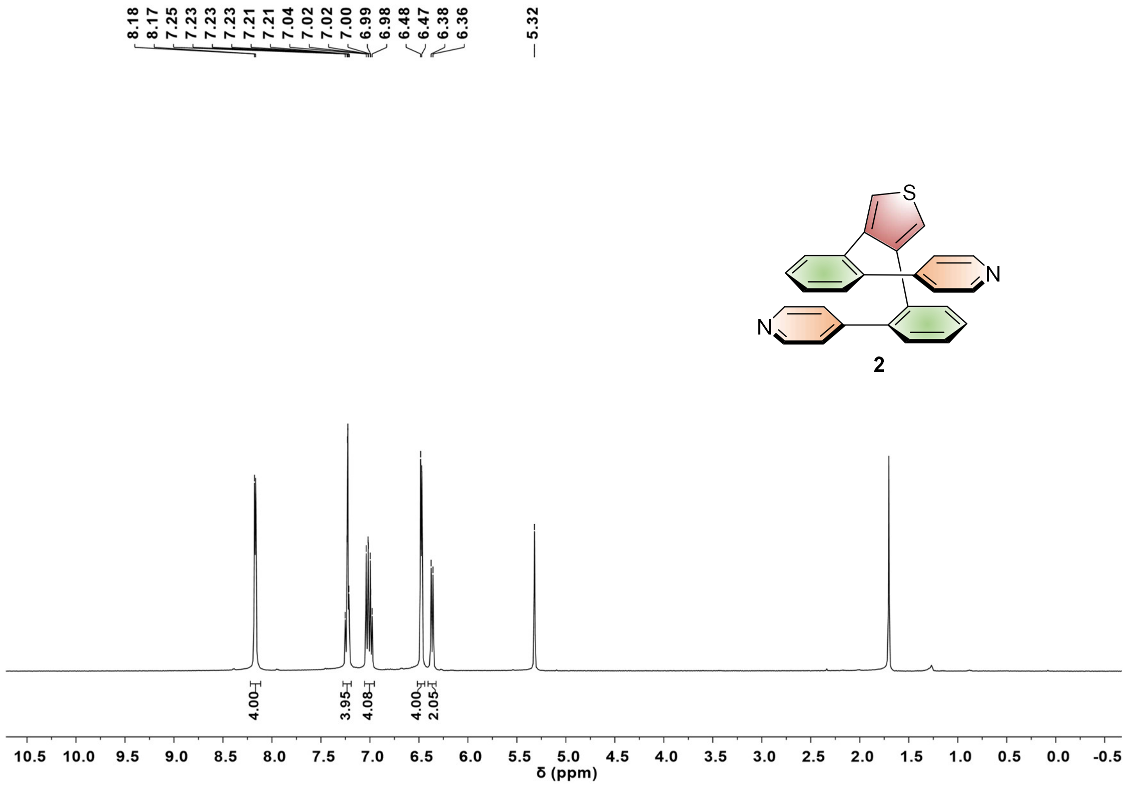


$^{1}$H–$^{1}$H COSY spectrum (400 MHz, $CD_2Cl_2$) of **2**.

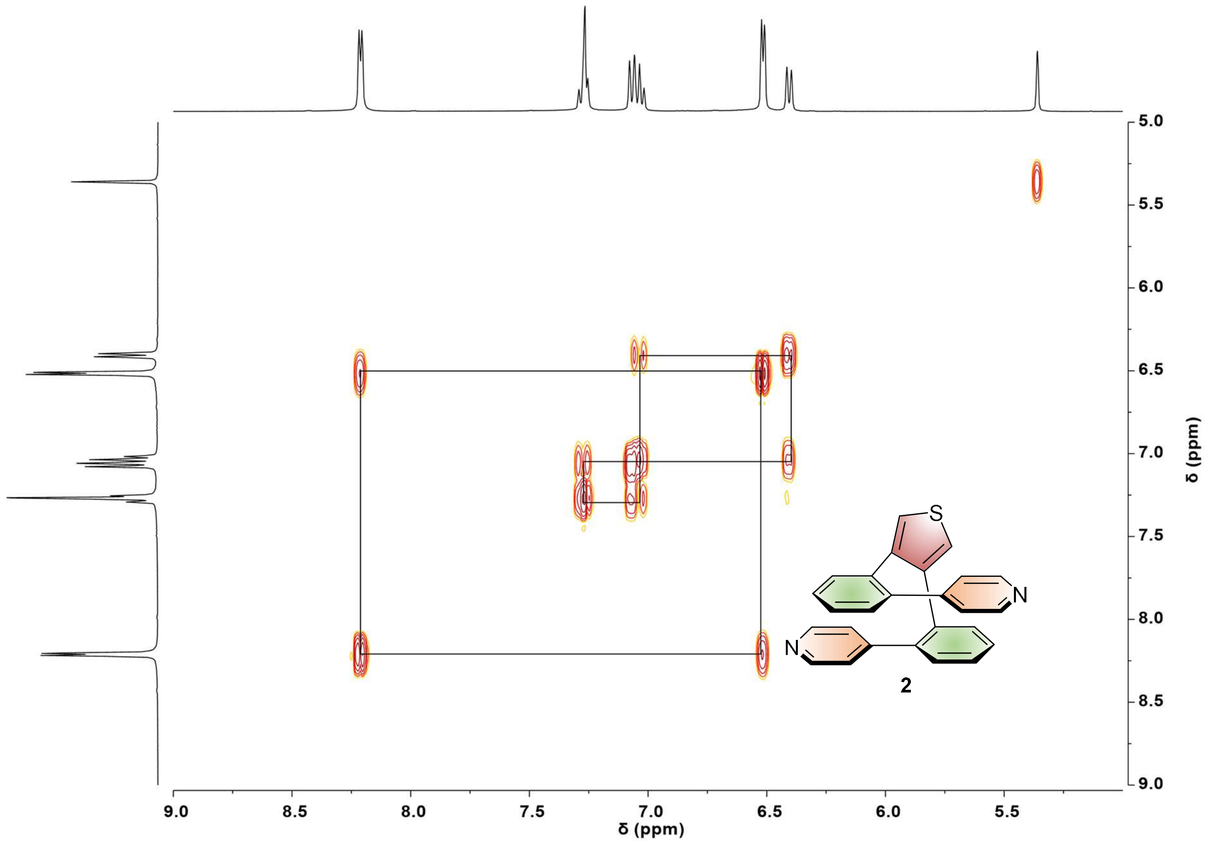

$^{13}$C NMR spectrum (400 MHz, $CD_2Cl_2$) of **2.**

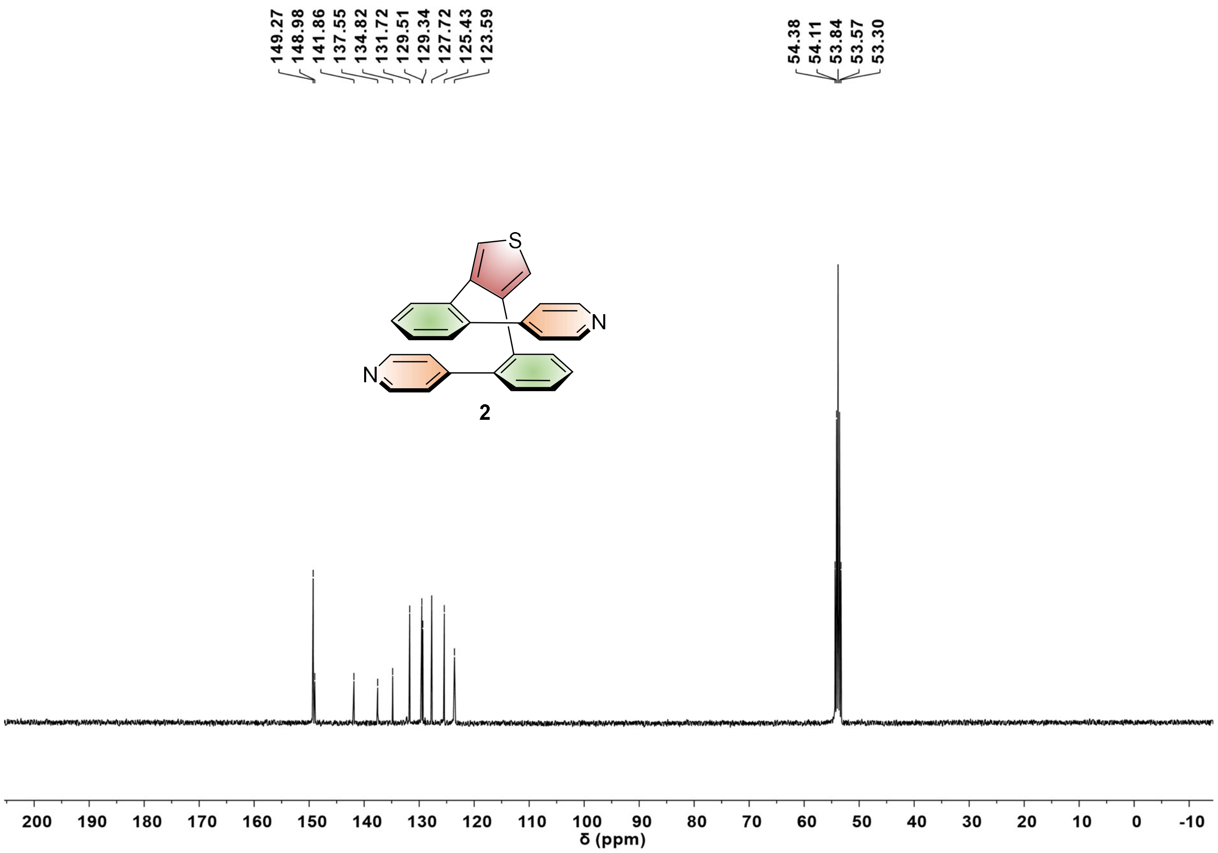


HRMS of **2**.

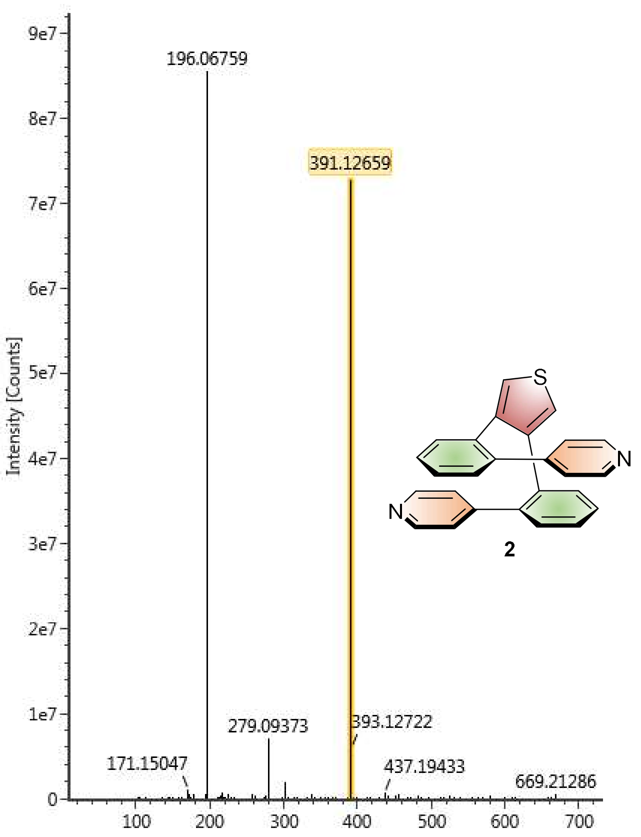

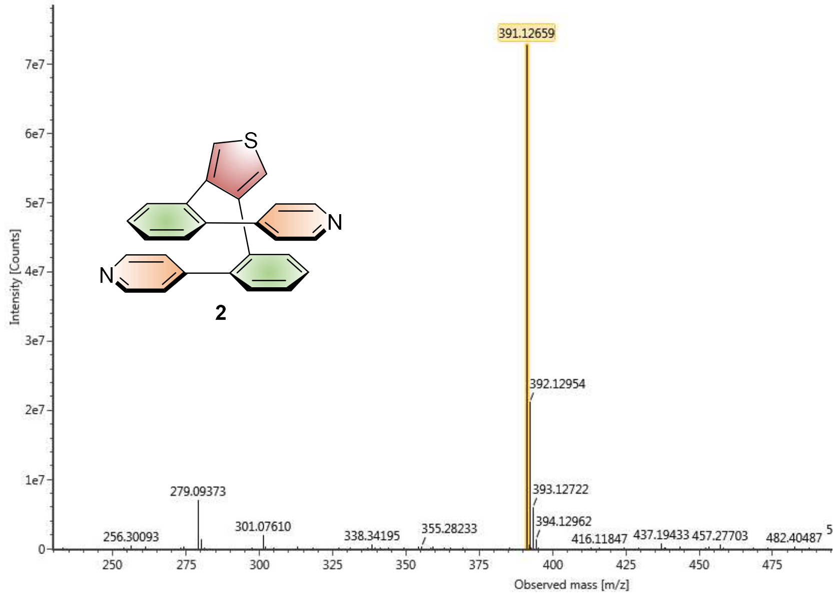
391.12659
392.12954
393.12722
394.12962
279.09373
301.07610
256.30093
338.34195
355.28233
416.11847
437.19433
457.27703
482.40487
Intensity [Counts]
Observed mass [m/z]
S
N
N
2

$^{1}H$ NMR spectrum (400 MHz, $CD_3CN$) of **3.**

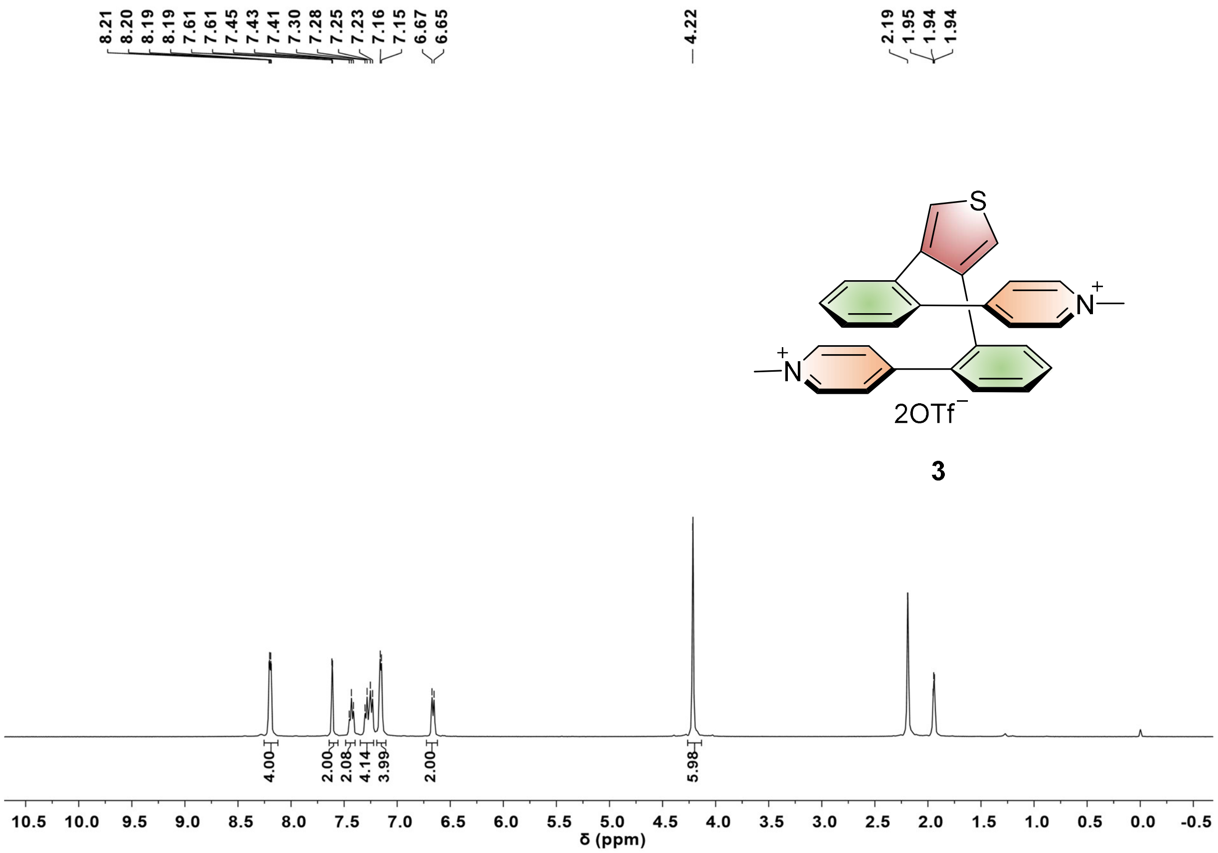


$^{1}H$-$^{1}H$ COSY spectrum (400 MHz, $CD_3CN$) of **3.**

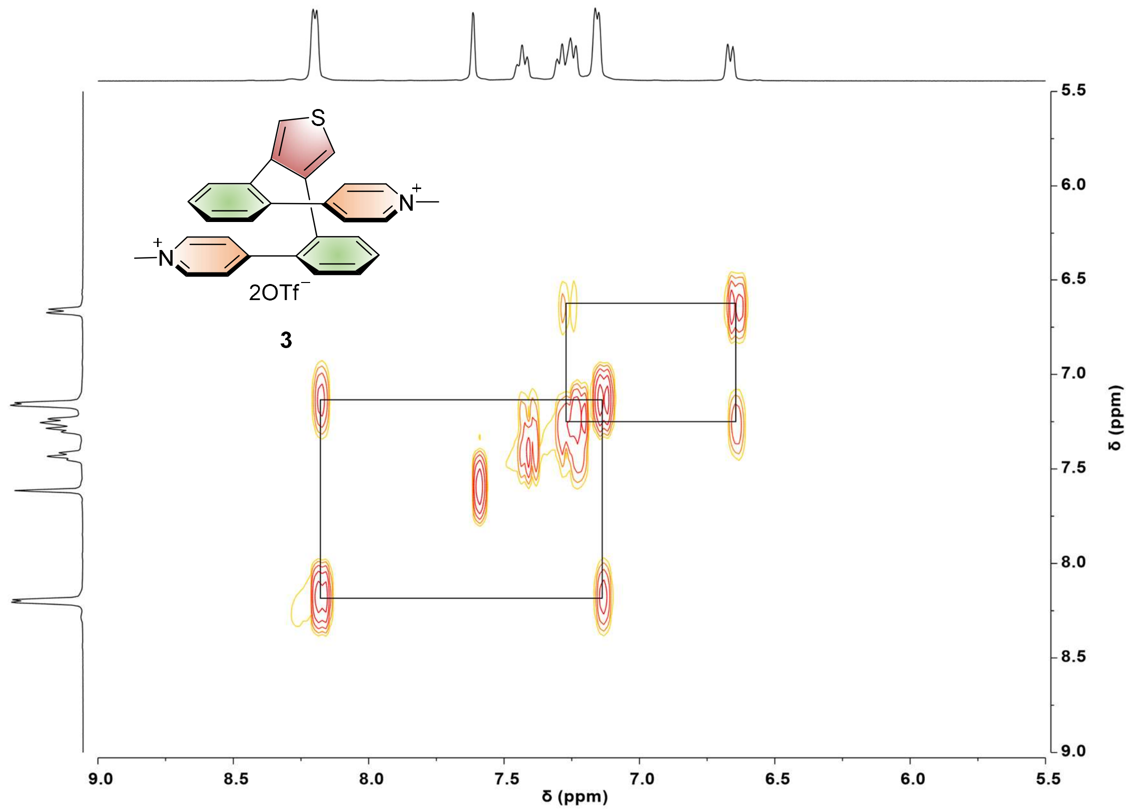

$^{13}C$ NMR spectrum (100 MHz, $CD_3CN$) of **3.**

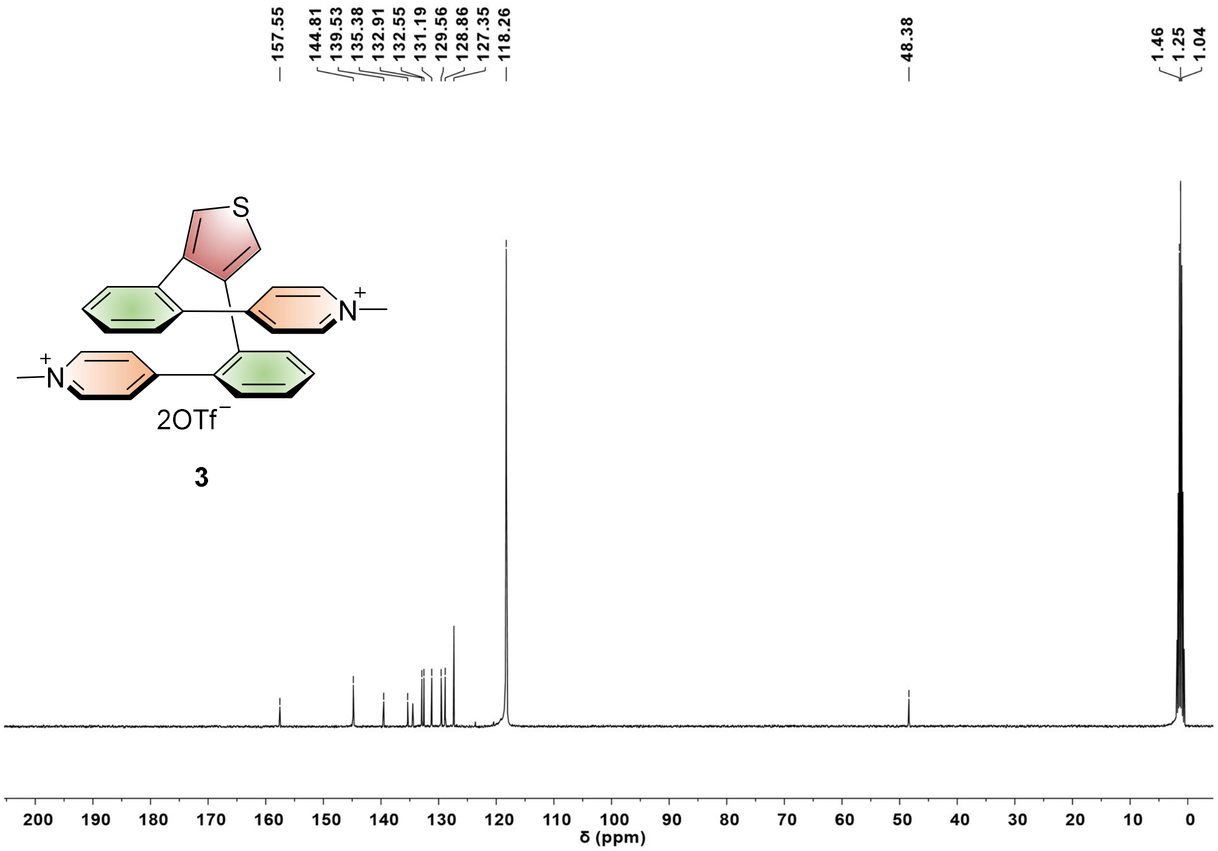


$^{19}F$ NMR spectrum (376 MHz, $CD_3CN$) of **3.**

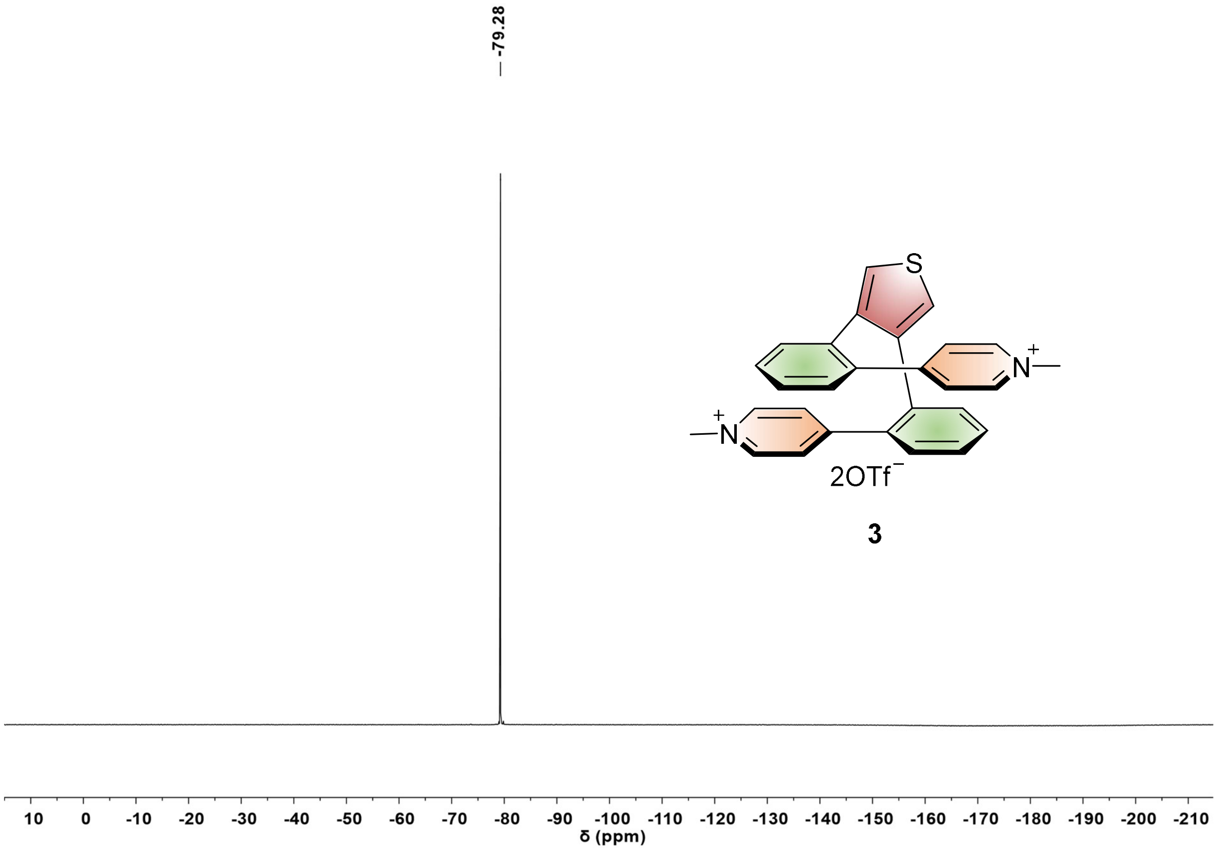

HRMS of **3**.

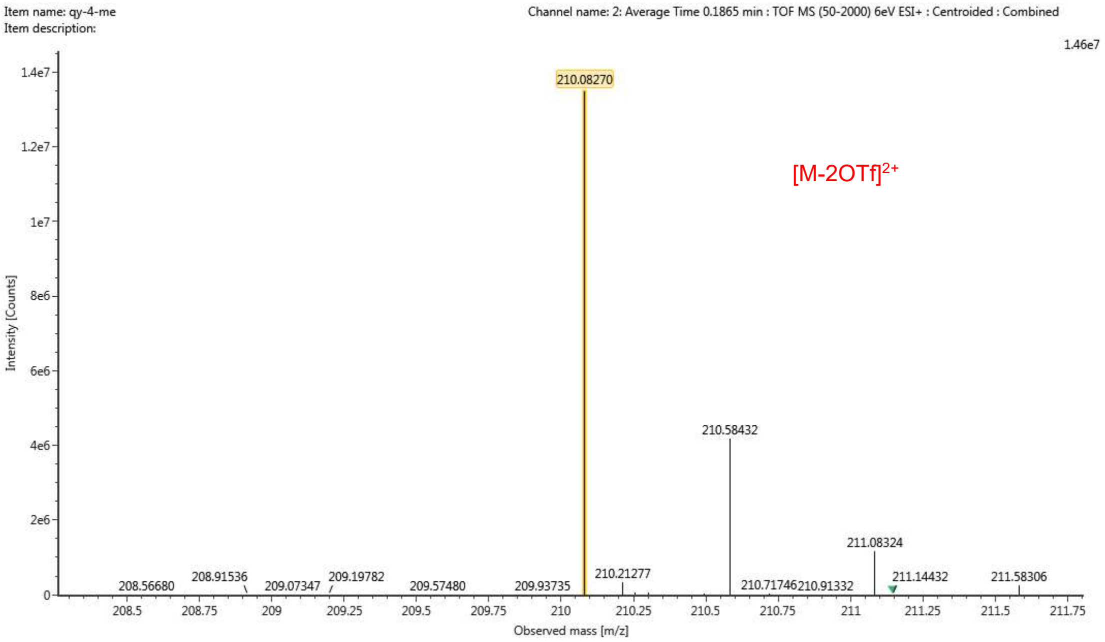
Item name: qy-4-me
Item description:
Channel name: 2: Average Time 0.1865 min : TOF MS (50-2000) 6eV ESI+ : Centroided : Combined
1.46e7
210.08270
[M-2OTf]2+
210.58432
211.08324
208.56680
208.91536
209.07347
209.19782
209.57480
209.93735
210.21277
210.71746
210.91332
211.14432
211.58306
Intensity [Counts]
Observed mass [m/z]

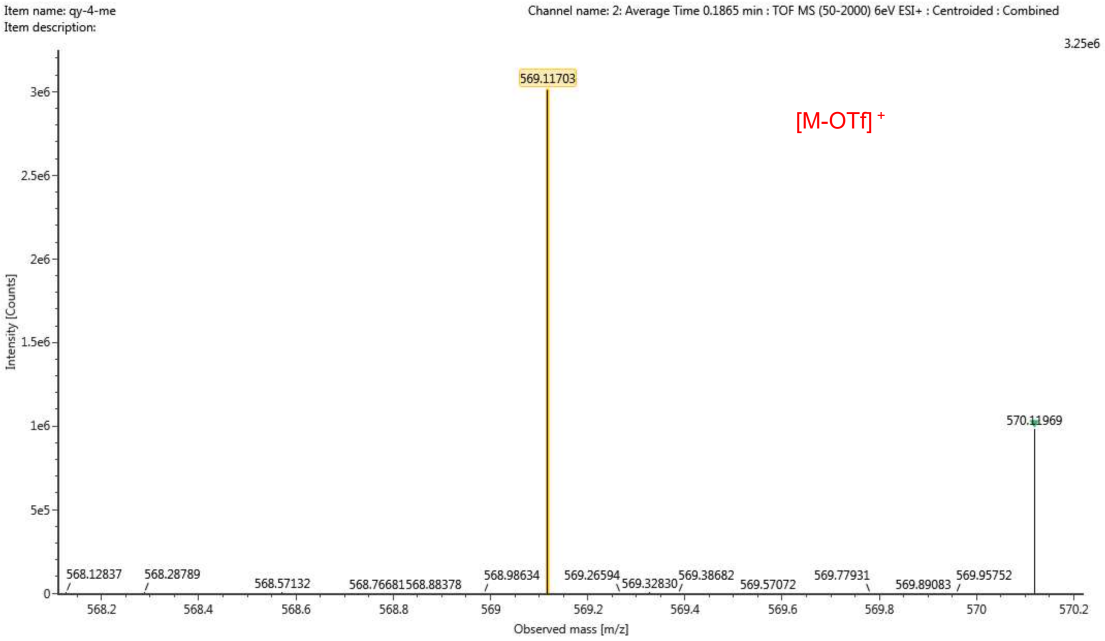
Item name: qy-4-me
Item description:
Channel name: 2: Average Time 0.1865 min : TOF MS (50-2000) 6eV ESI+ : Centroided : Combined
3.25e6
569.11703
[M-OTf] +
570.11969
568.12837
568.28789
568.57132
568.76681
568.88378
568.98634
569.26594
569.32830
569.38682
569.57072
569.77931
569.89083
569.95752
Intensity [Counts]
Observed mass [m/z]

$^{1}$H NMR spectrum (400 MHz, DMSO-$d_6$) of **4.**

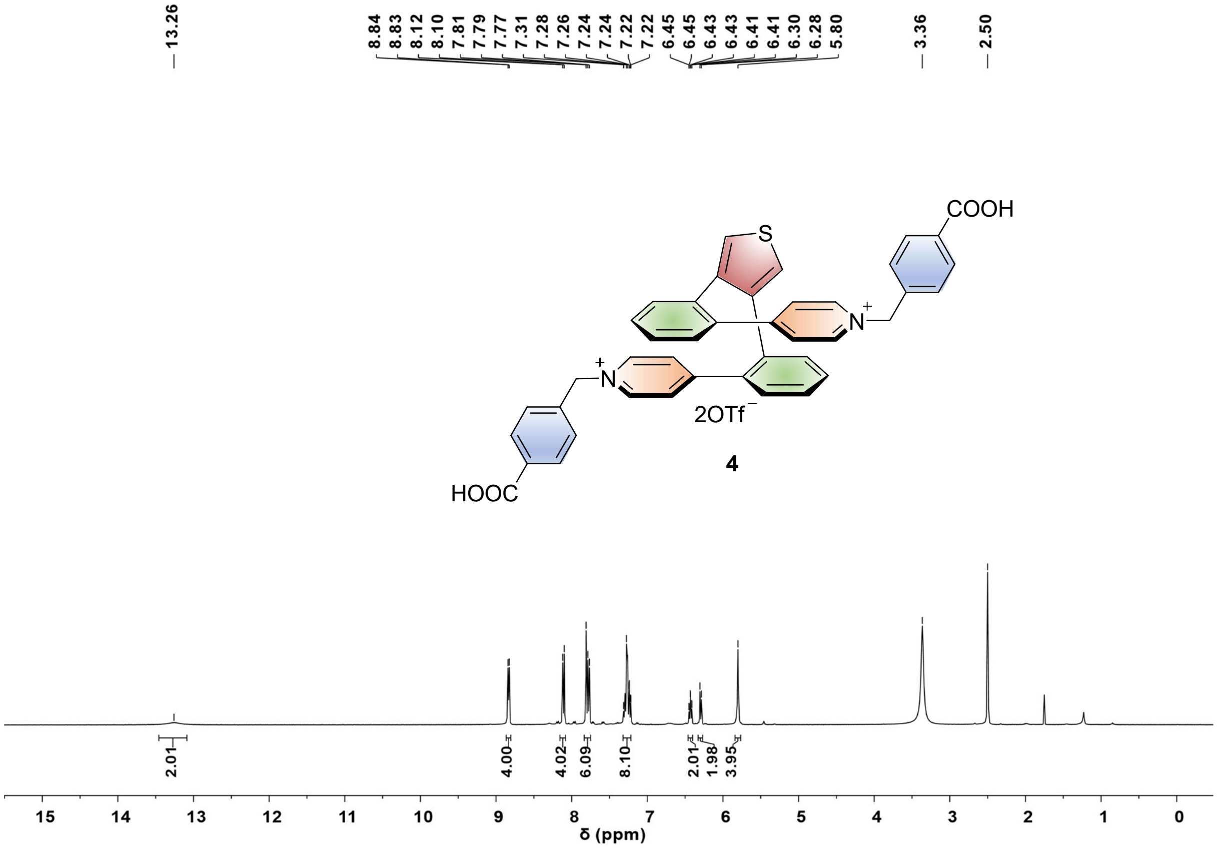


$^{1}$H-$^{1}$H COSY spectrum (400 MHz, DMSO-$d_6$) of **4.**

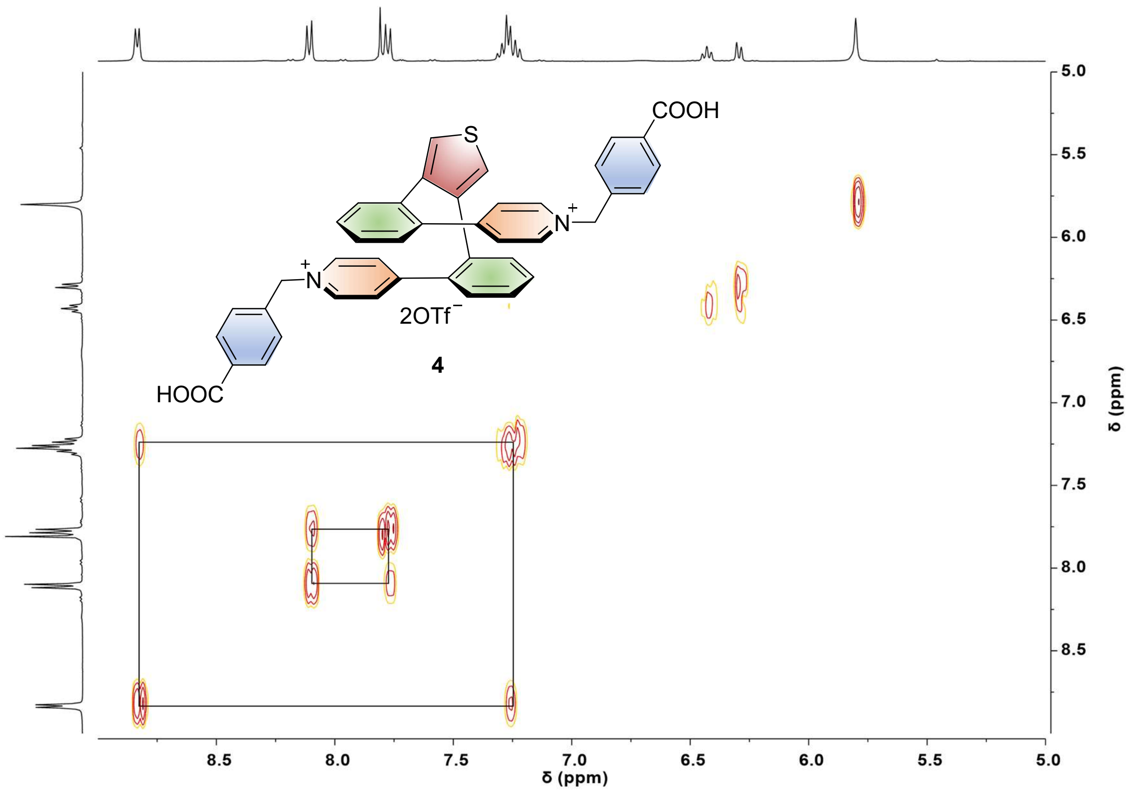

$^{13}$C NMR spectrum (100 MHz, DMSO-$d_6$) of **4.**

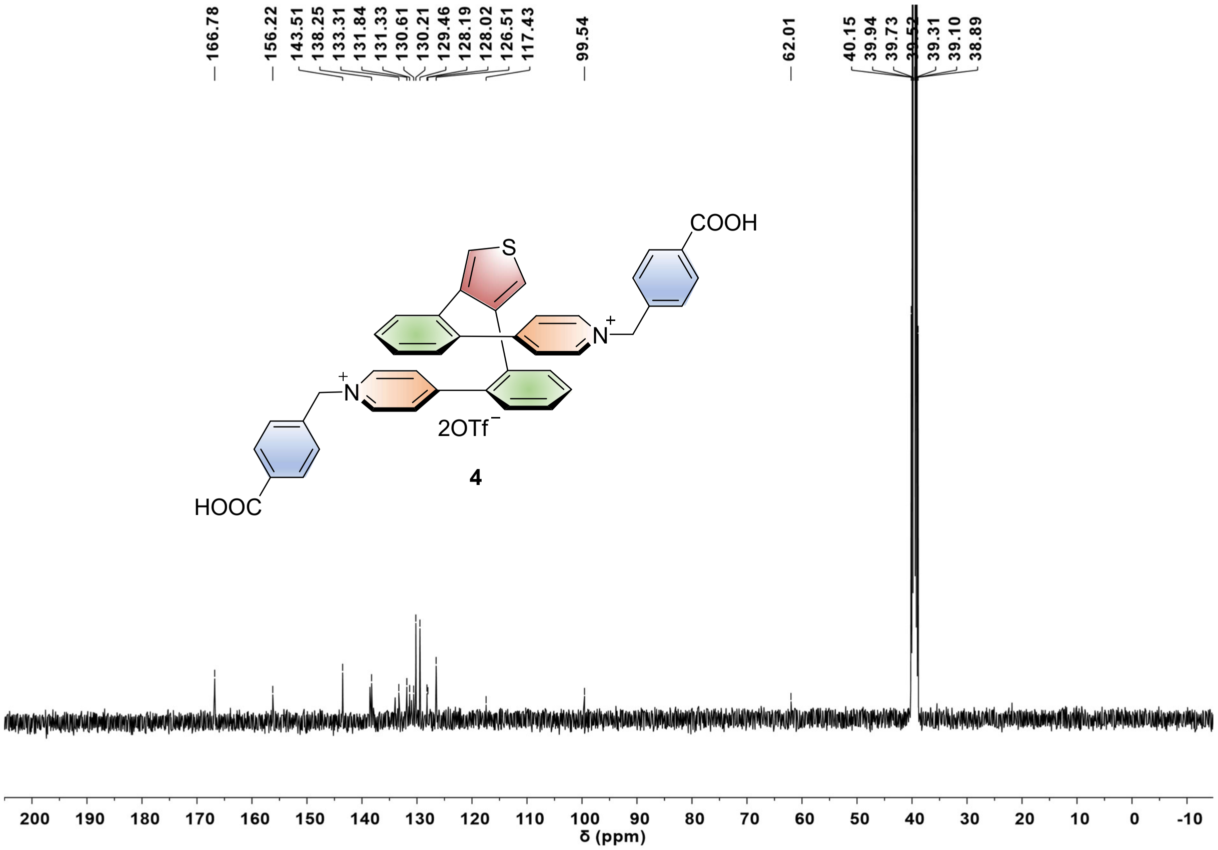


$^{19}$F NMR spectrum (376 MHz, DMSO-$d_6$) of **4.**

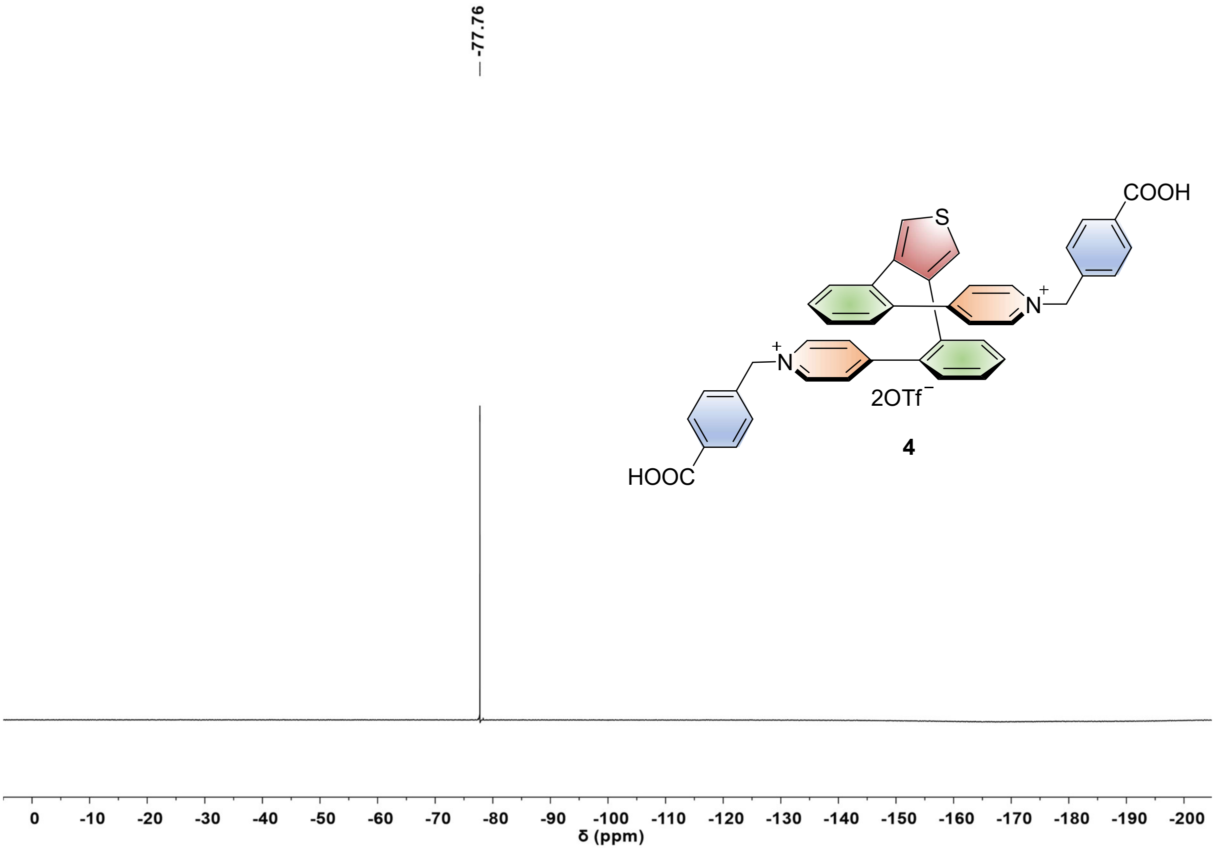

HRMS of **4**.

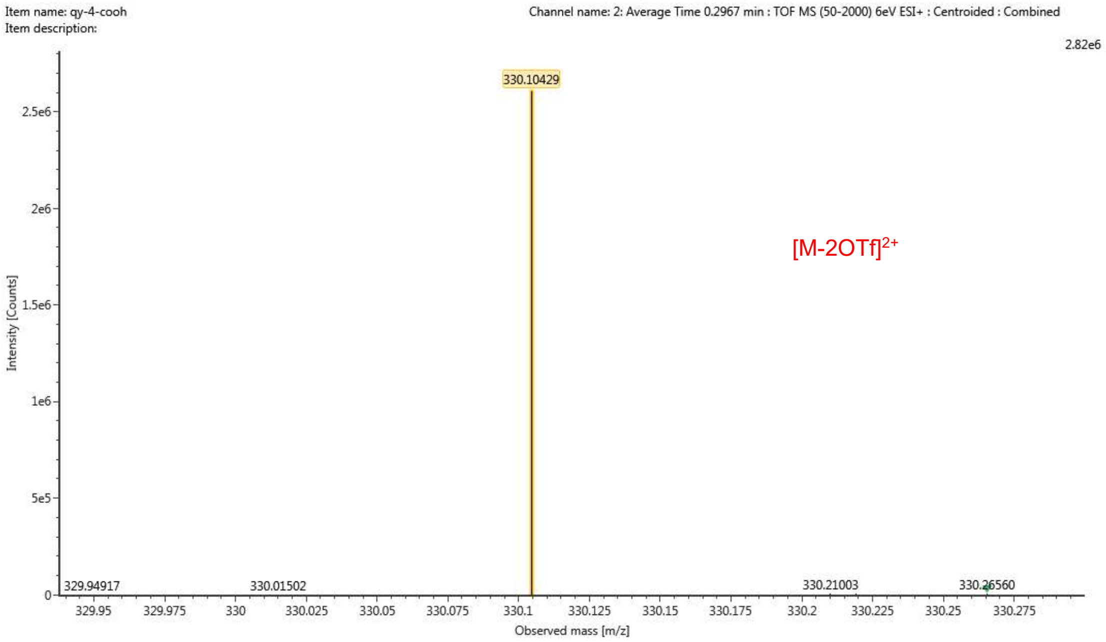
Item name: qy-4-cooh
Item description:
Channel name: 2: Average Time 0.2967 min : TOF MS (50-2000) 6eV ESI+ : Centroided : Combined
2.82e6
330.10429
[M-2OTf]2+
329.94917
330.01502
330.21003
330.26560
Intensity [Counts]
Observed mass [m/z]

$^1$H NMR spectrum (400 MHz, $CD_3CN$) of **5.**

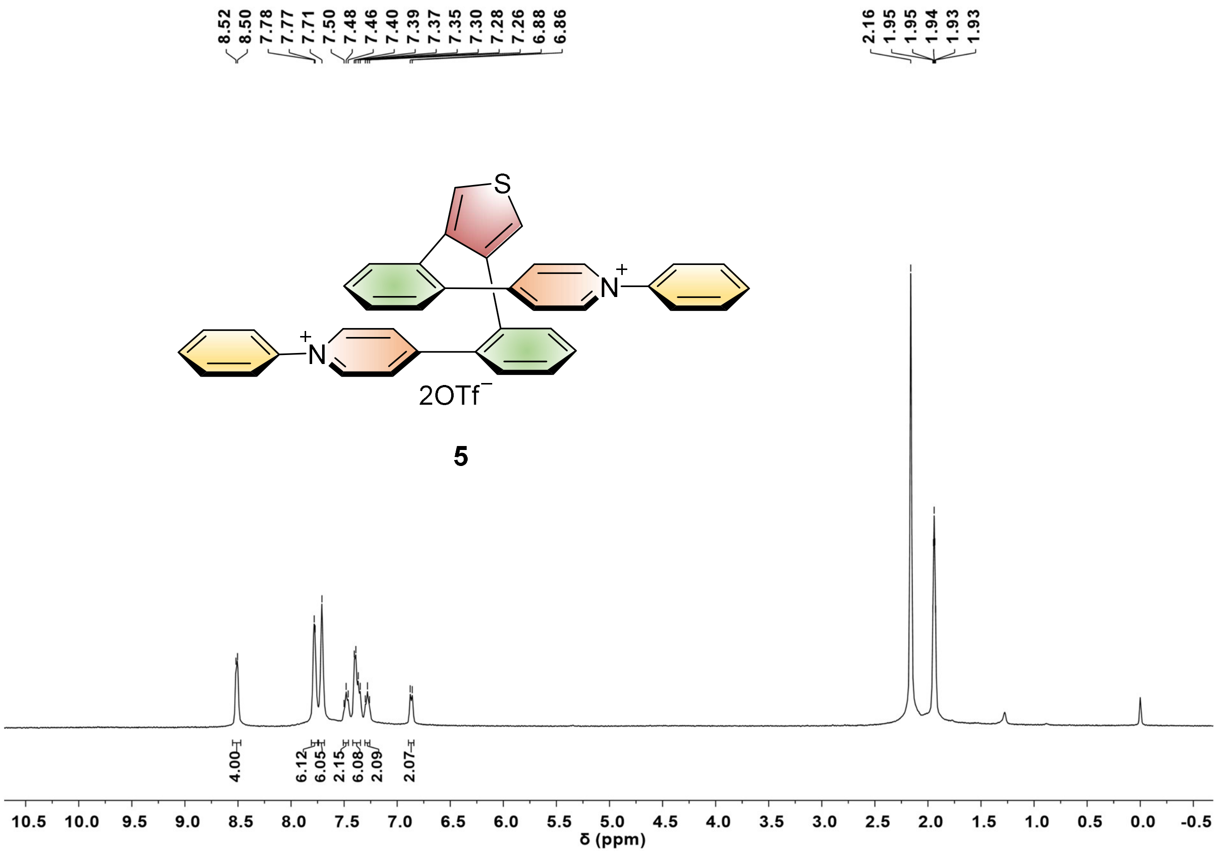


$^1$H-$^1$H COSY spectrum (400 MHz, $CD_3CN$) of **5.**

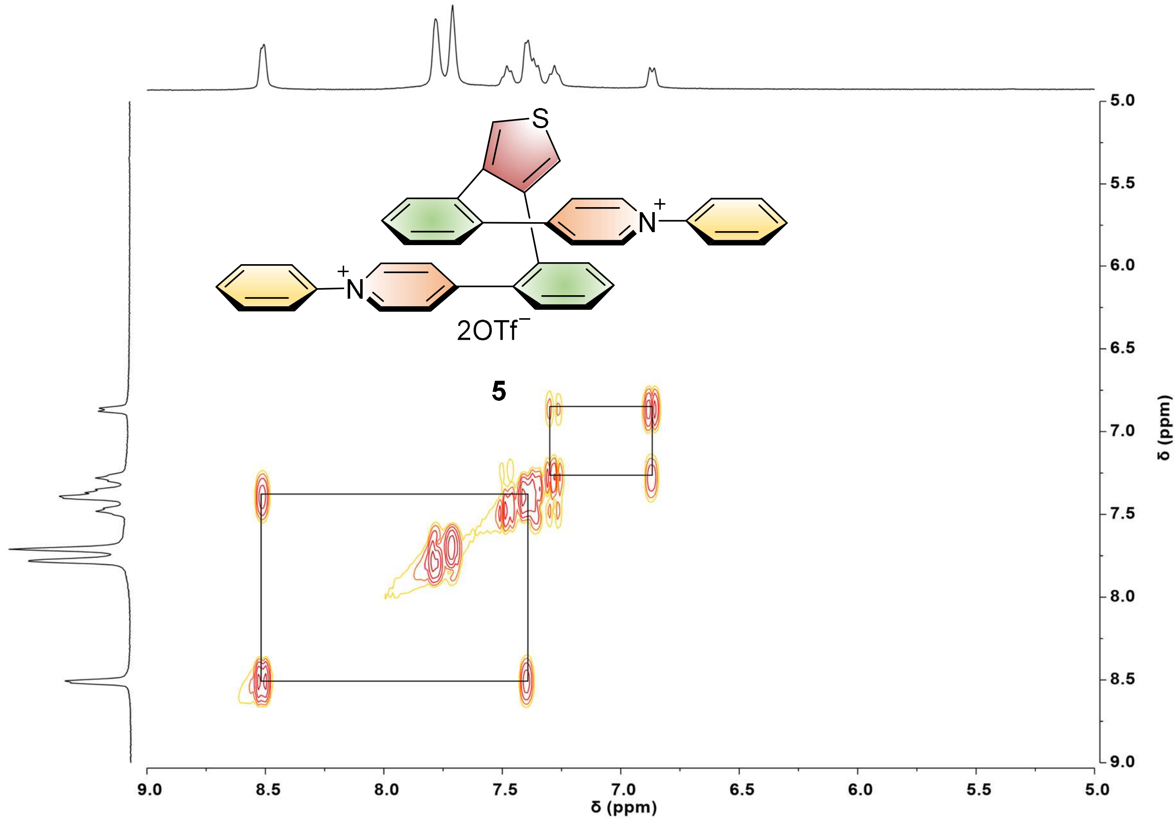

$^{13}C$ NMR spectrum (100 MHz, $CD_3CN$) of **5.**

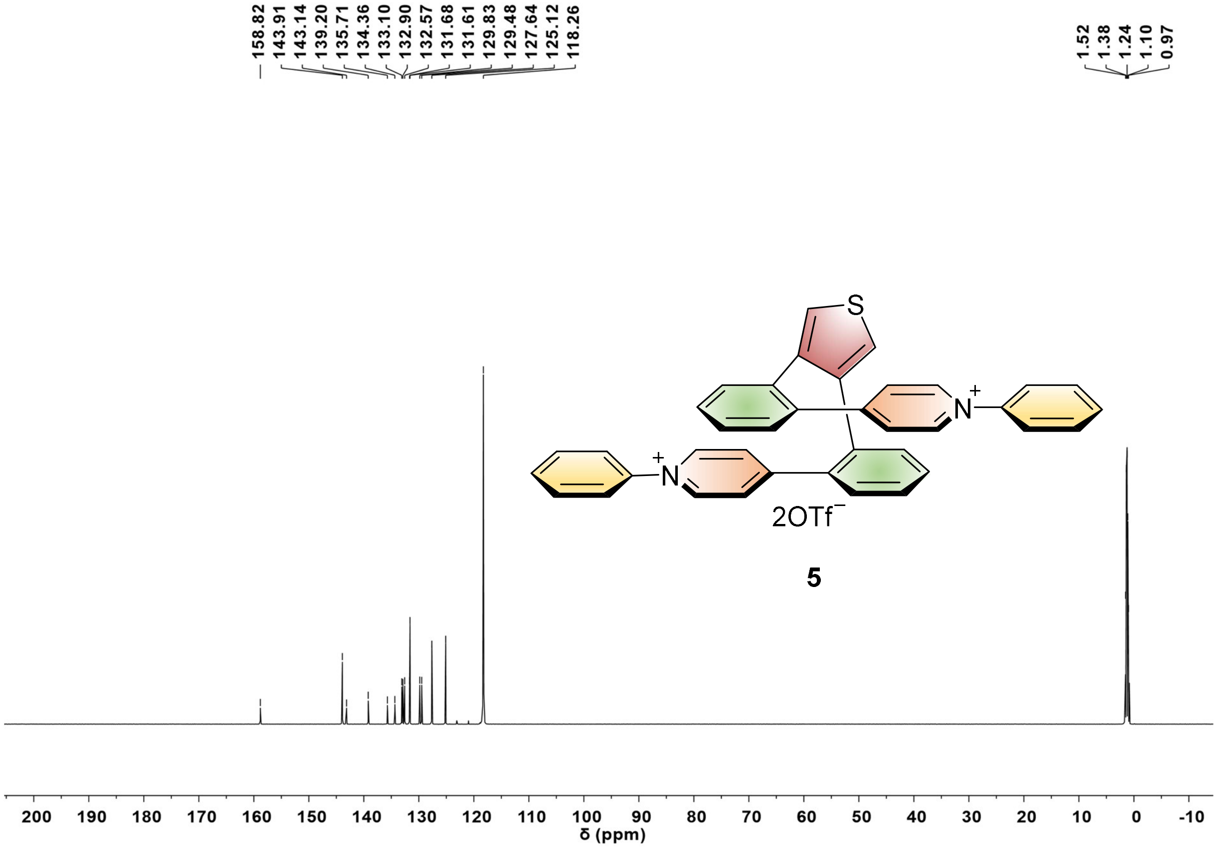


$^{19}F$ NMR spectrum (376 MHz, DMSO-$d_6$) of **5.**

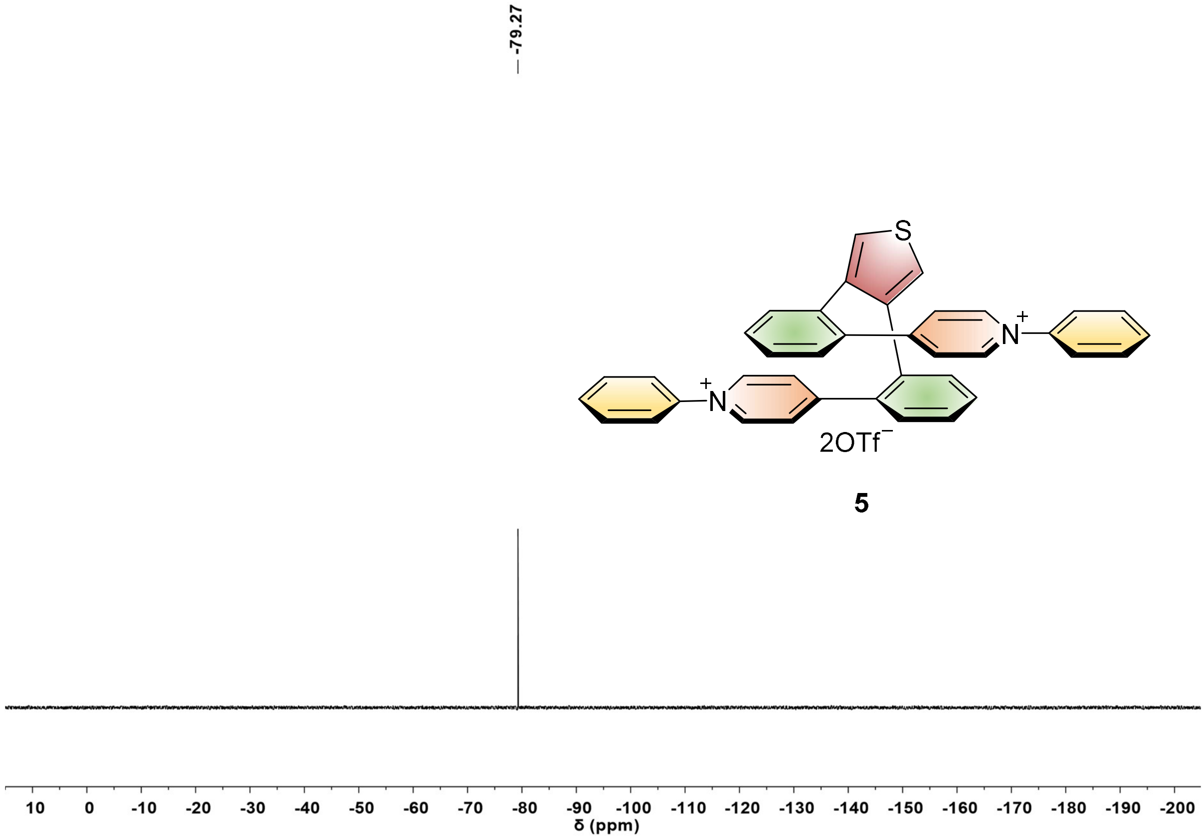

HRMS of **5**.

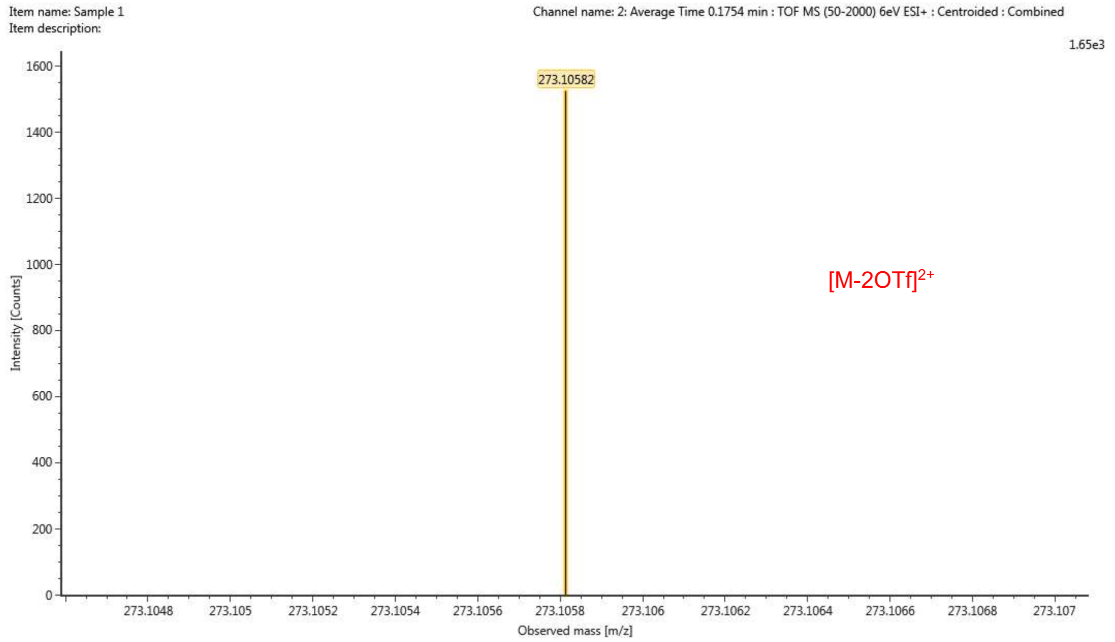
Item name: Sample 1
Item description:
Channel name: 2: Average Time 0.1754 min : TOF MS (50-2000) 6eV ESI+ : Centroided : Combined
1.65e3
273.10582
[M-2OTf]2+
Intensity [Counts]
Observed mass [m/z]

$^{1}$H NMR spectrum (400 MHz, $CD_2Cl_2$) of **6.**

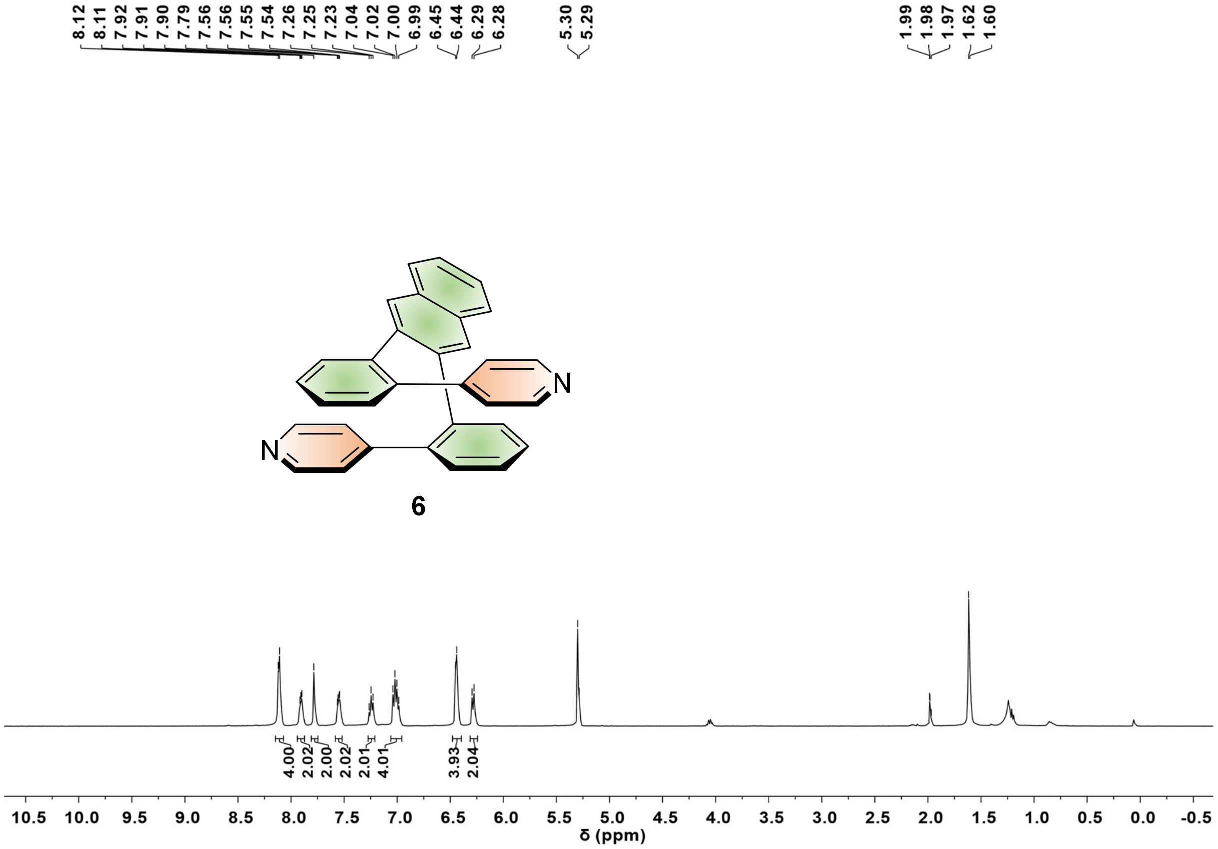


$^{1}$H-$^{1}$H COSY spectrum (400 MHz, $CD_2Cl_2$) of **6.**

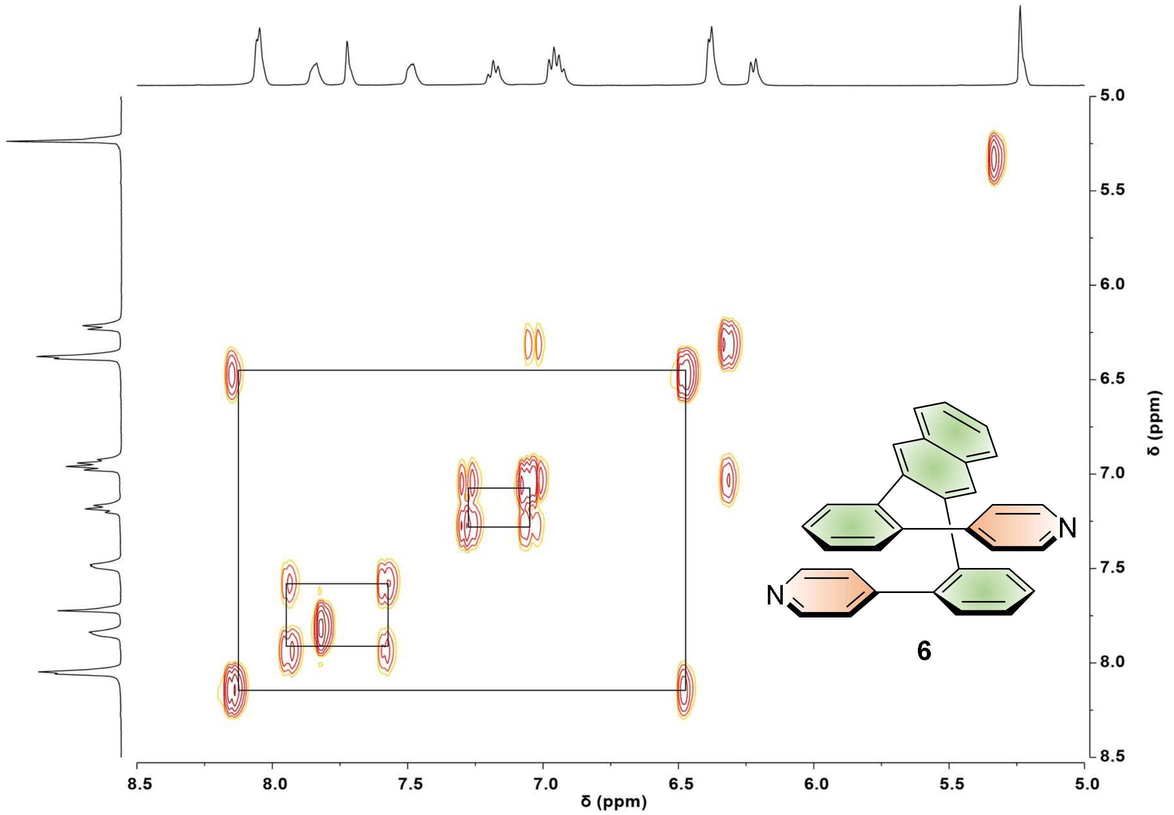

$^{13}C$ NMR spectrum (100 MHz, $CD_2Cl_2$) of **6.**

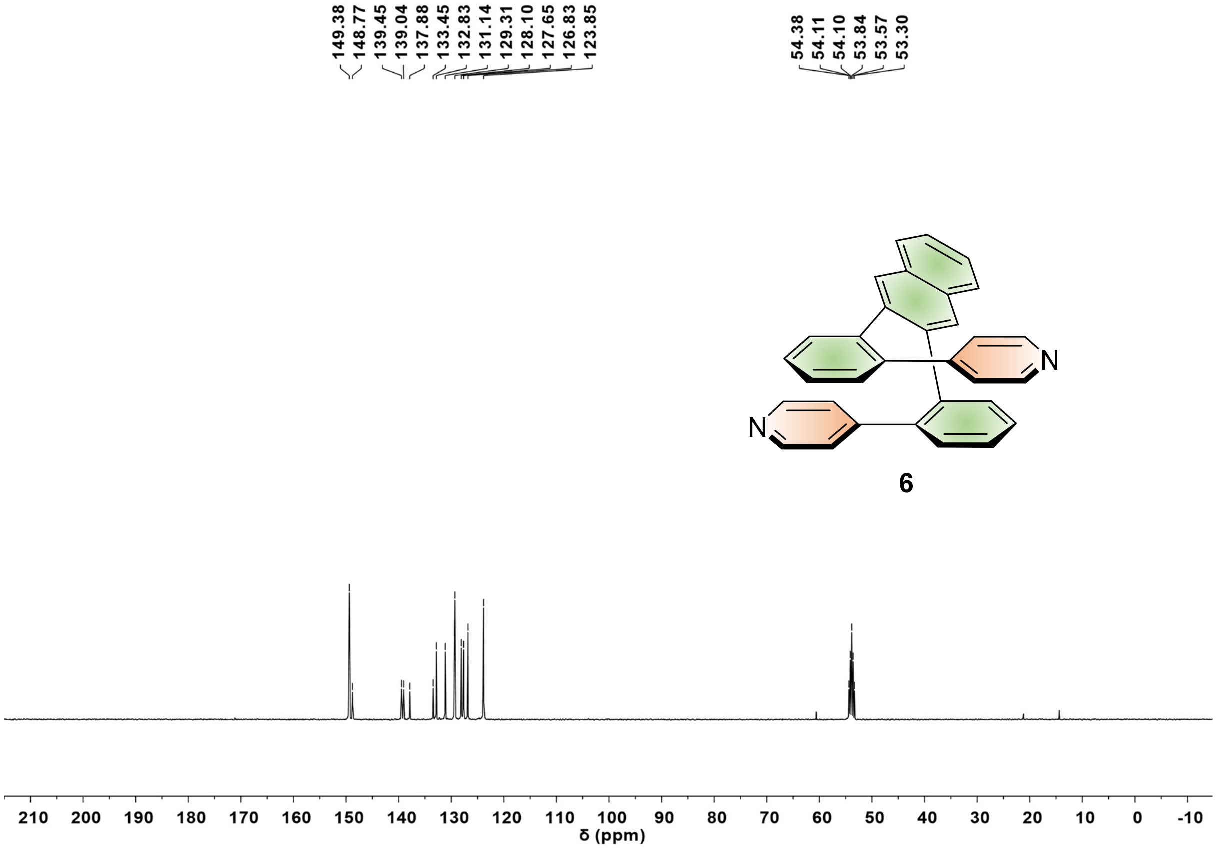


HRMS of **6**.

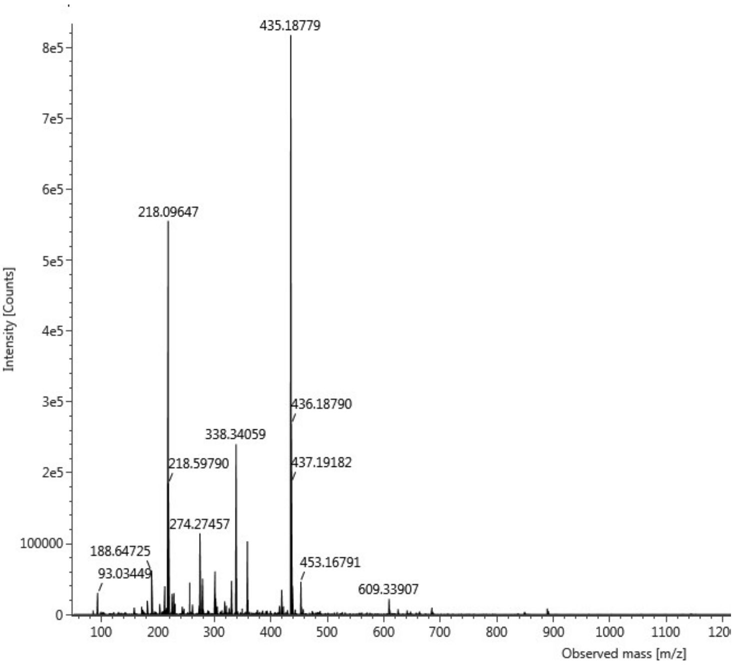

$^1$H NMR spectrum (400 MHz, $CD_3CN$) of **7.**

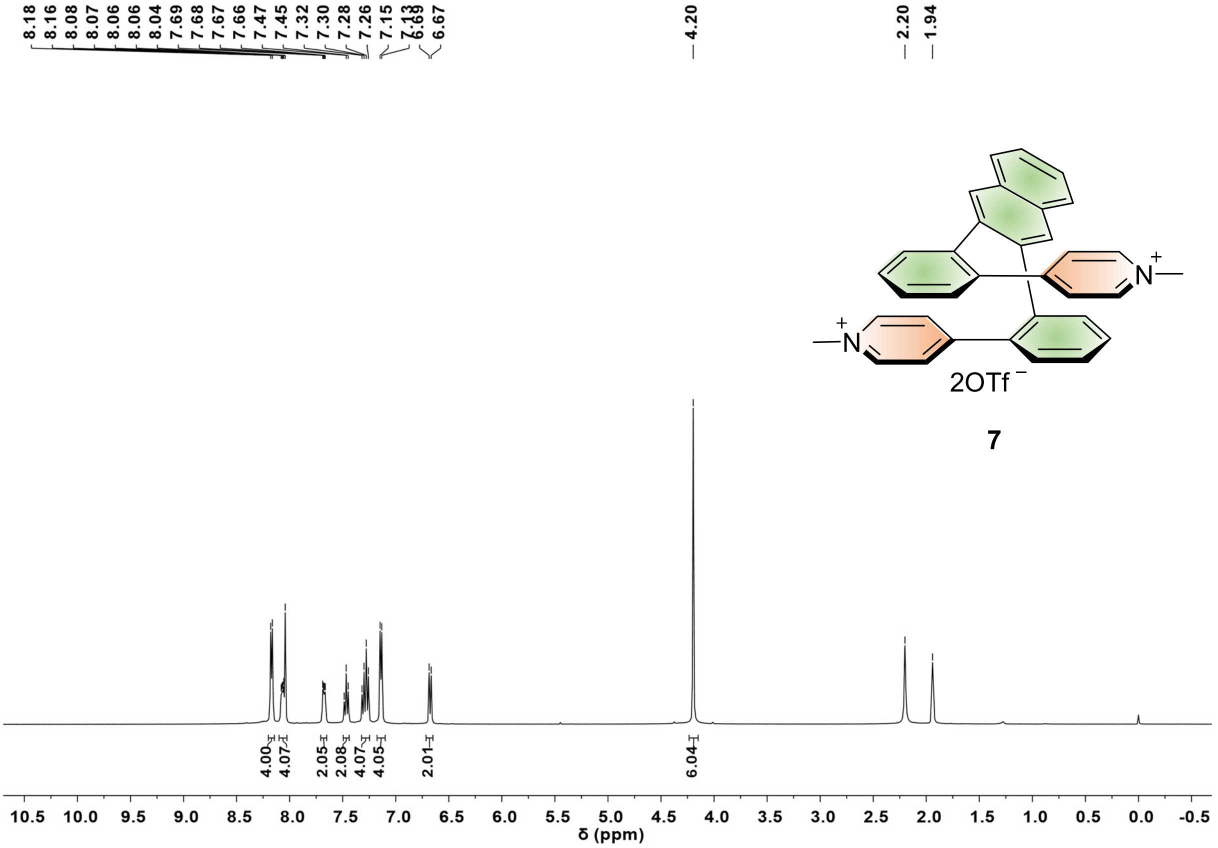


$^1$H-$^1$H COSY spectrum (400 MHz, $CD_3CN$) of **7.**

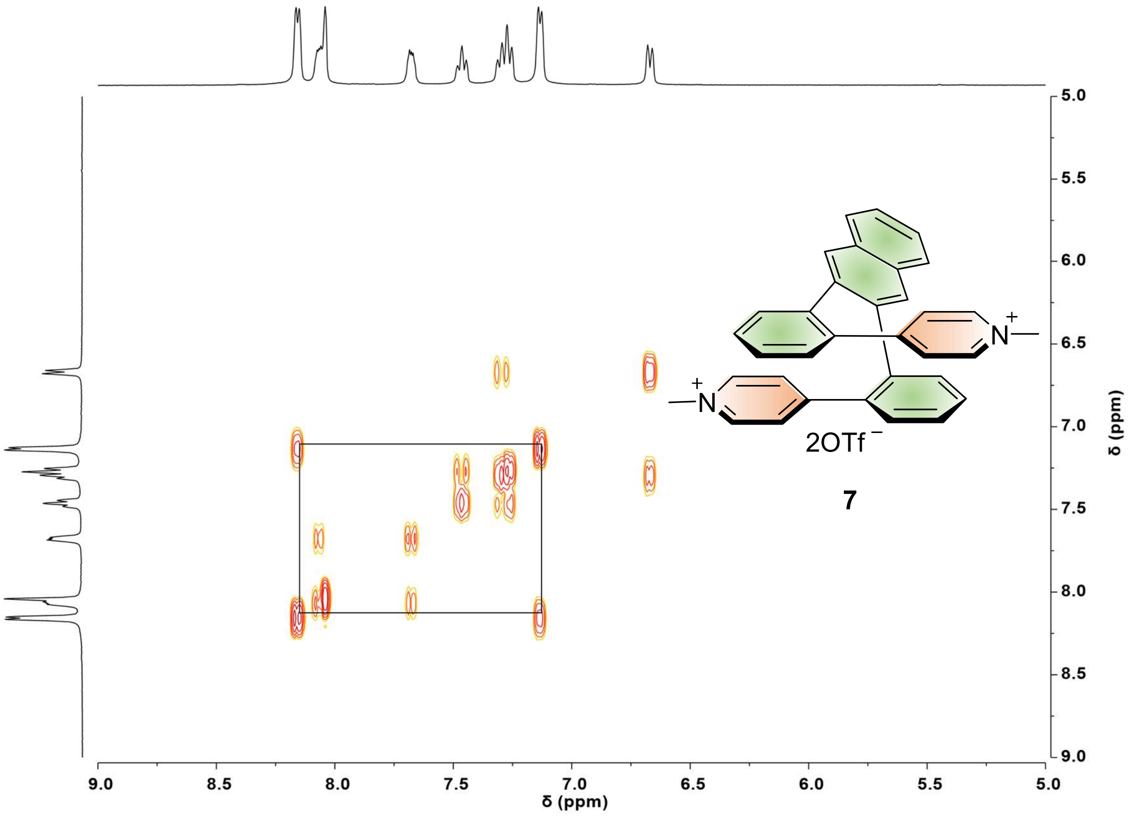

$^{13}$C NMR spectrum (100 MHz, $CD_3CN$) of **7.**

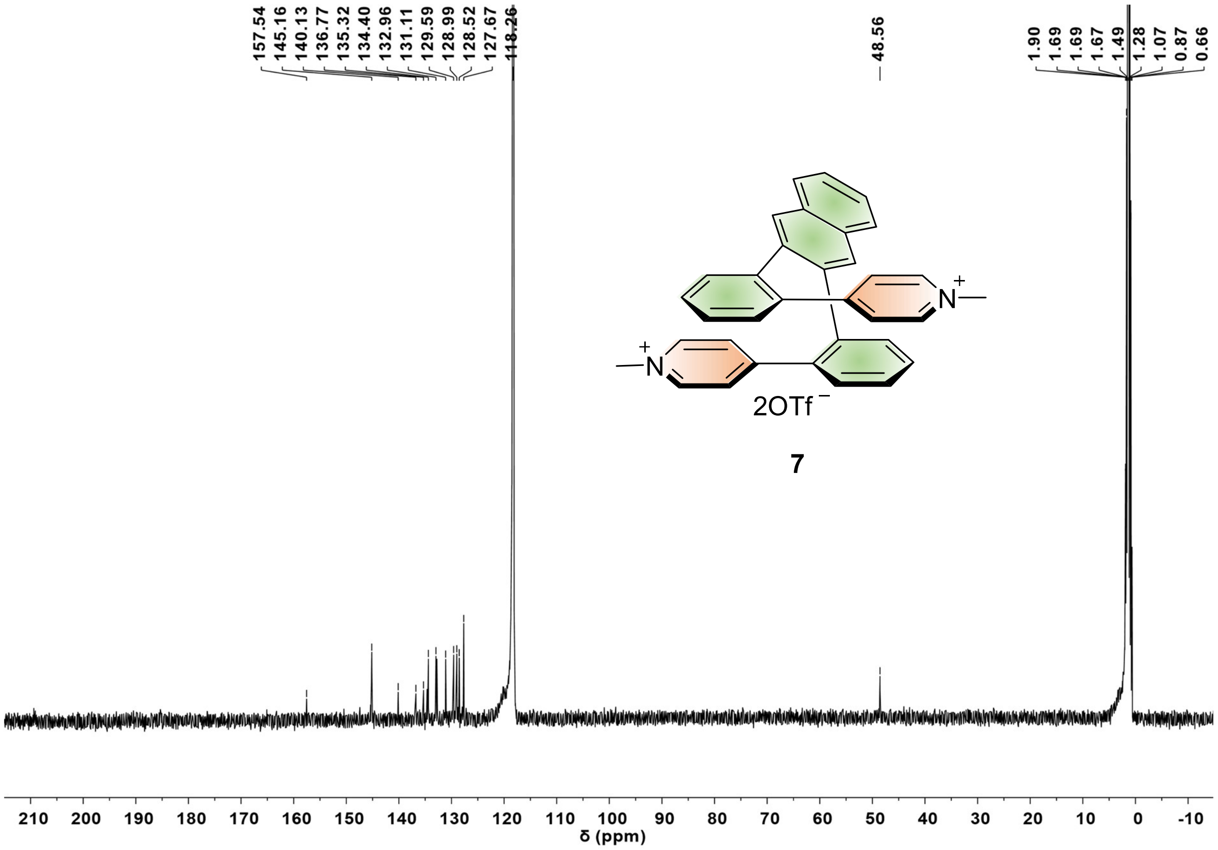


$^{19}$F NMR spectrum (376 MHz, $CD_3CN$) of **7.**

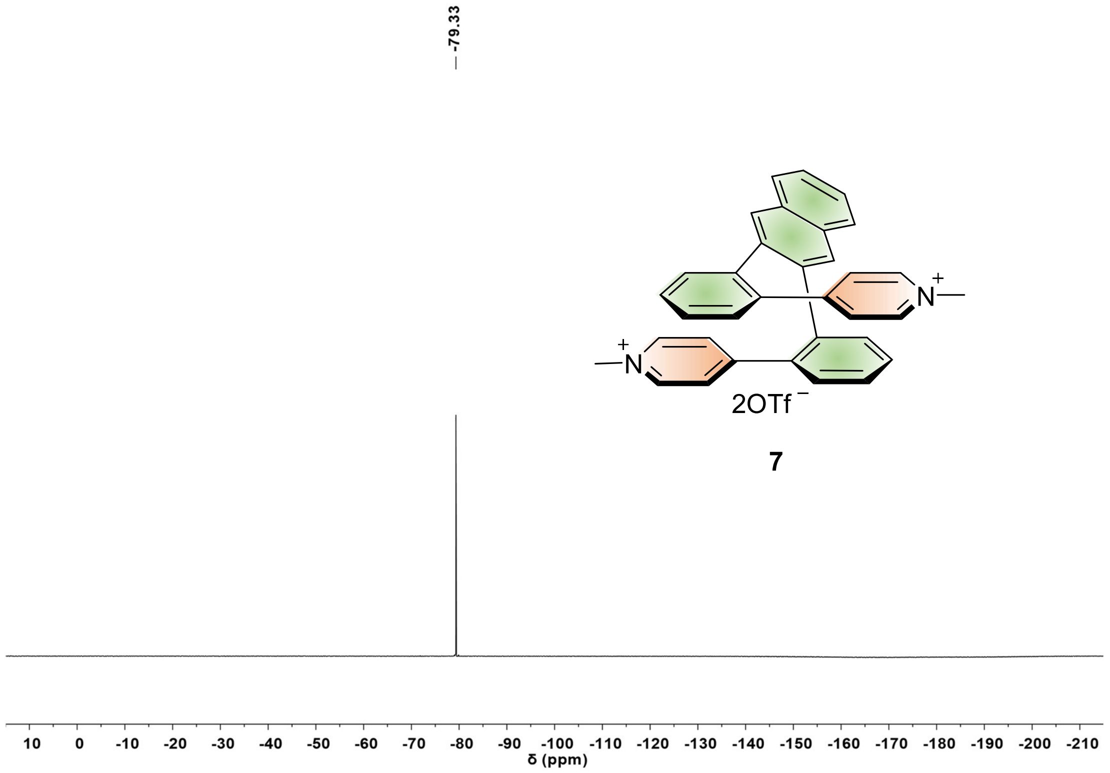

HRMS of **7**.

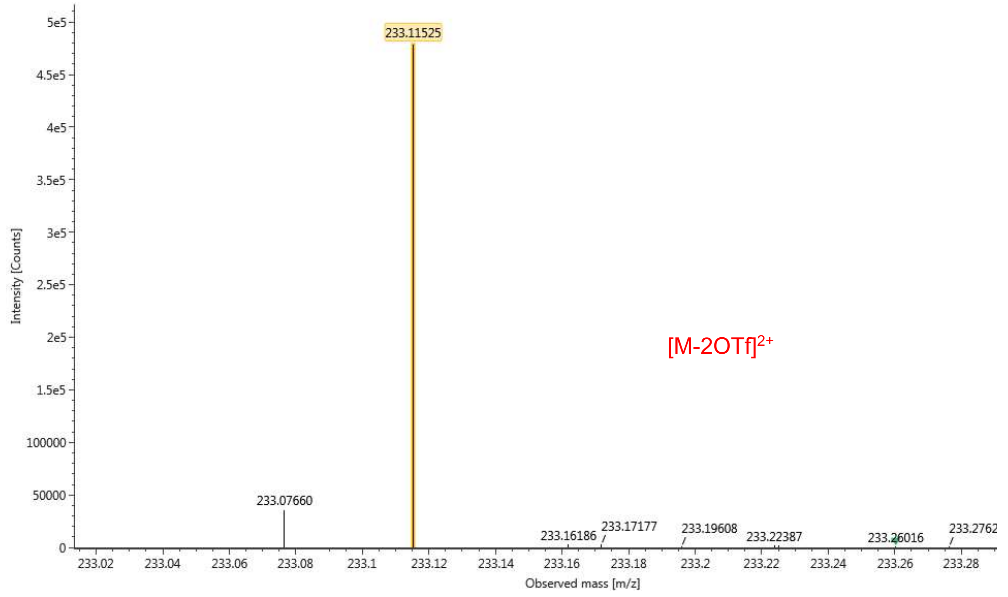
233.11525
233.07660
233.16186
233.17177
233.19608
233.22387
233.26016
233.2762
[M-2OTf]2+
Intensity [Counts]
Observed mass [m/z]

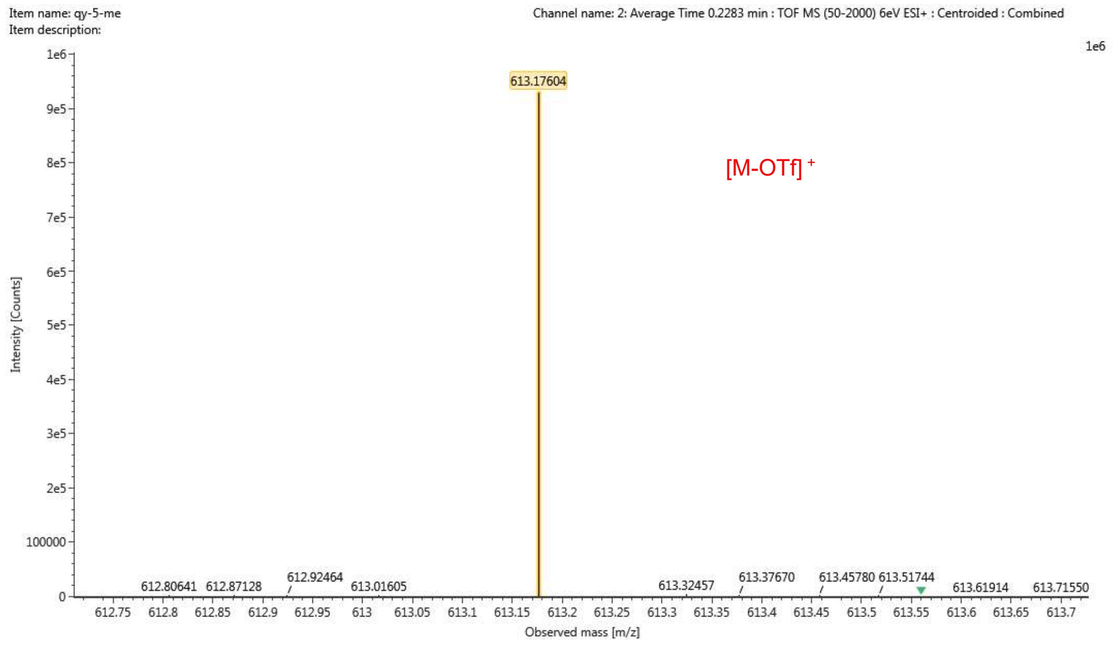
Item name: qy-5-me
Item description:
Channel name: 2: Average Time 0.2283 min : TOF MS (50-2000) 6eV ESI+ : Centroided : Combined
1e6
613.17604
[M-OTf] +
612.80641
612.87128
612.92464
613.01605
613.32457
613.37670
613.45780
613.51744
613.61914
613.71550
Intensity [Counts]
Observed mass [m/z]

$^{1}$H NMR spectrum (400 MHz, DMSO-$d_6$) of **8.**

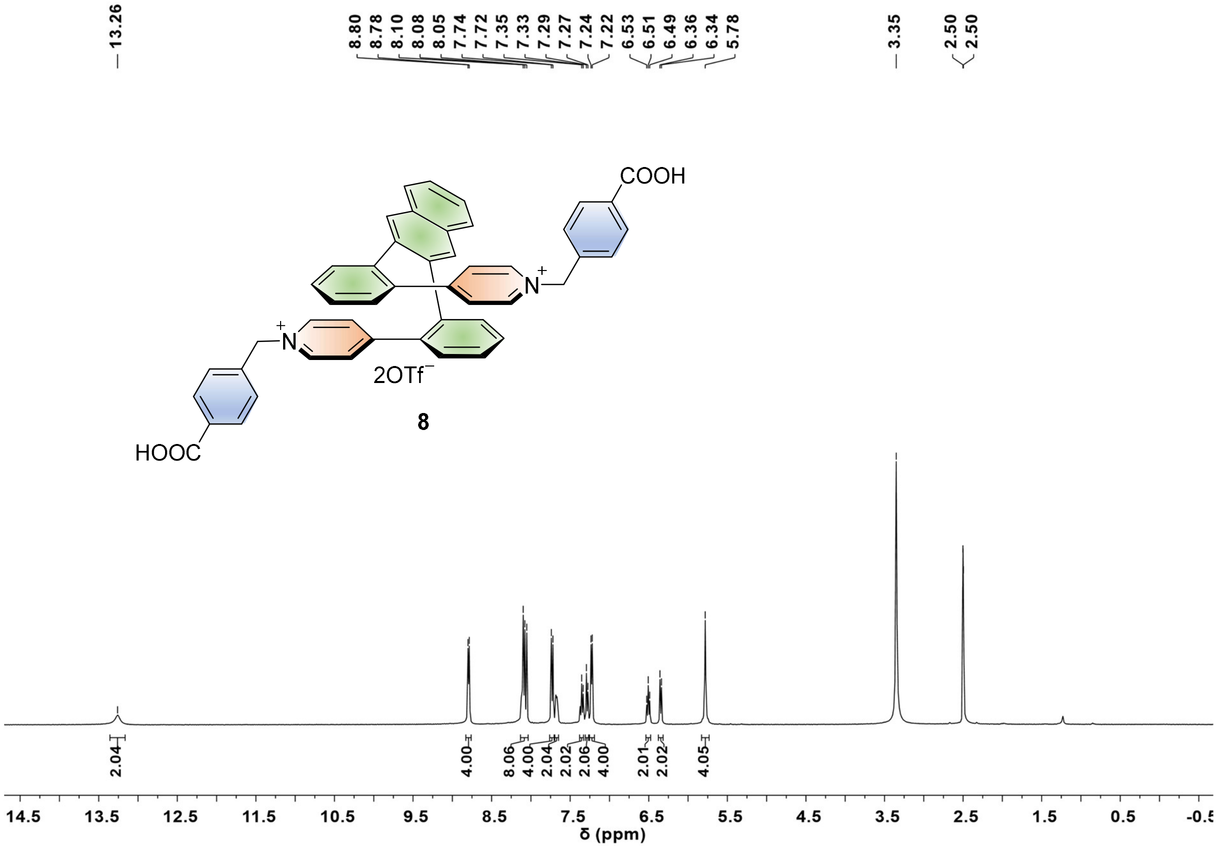


$^{1}$H-$^{1}$H COSY spectrum (400 MHz, DMSO-$d_6$) of **8.**

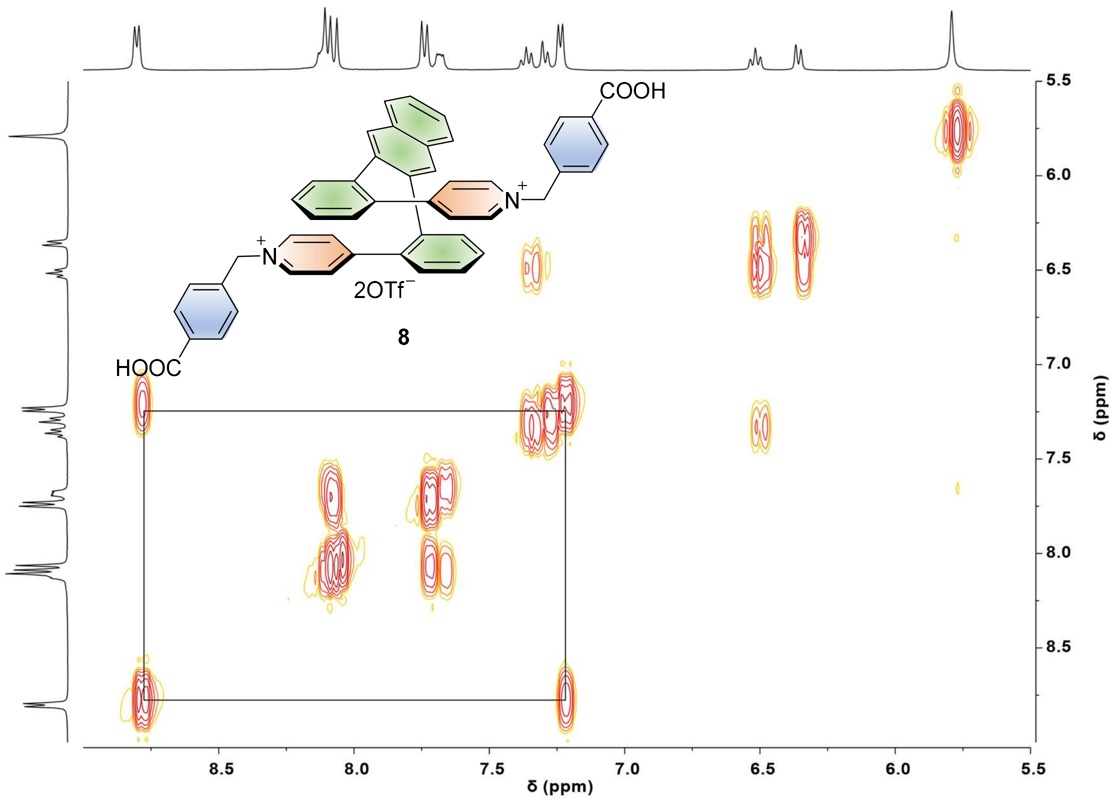

$^{13}$C NMR spectrum (100 MHz, DMSO-$d_6$) of **8.**

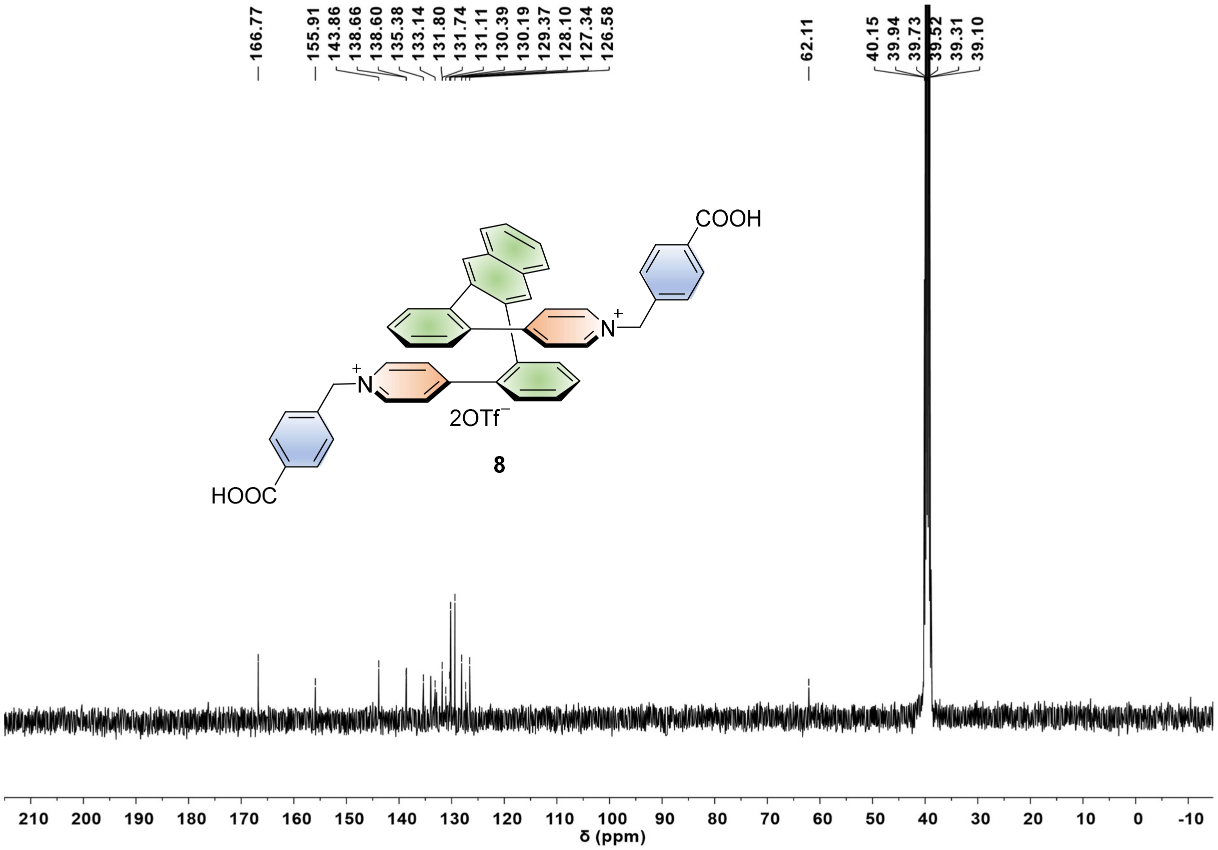


$^{19}$F NMR spectrum (376 MHz, DMSO-$d_6$) of **8**.

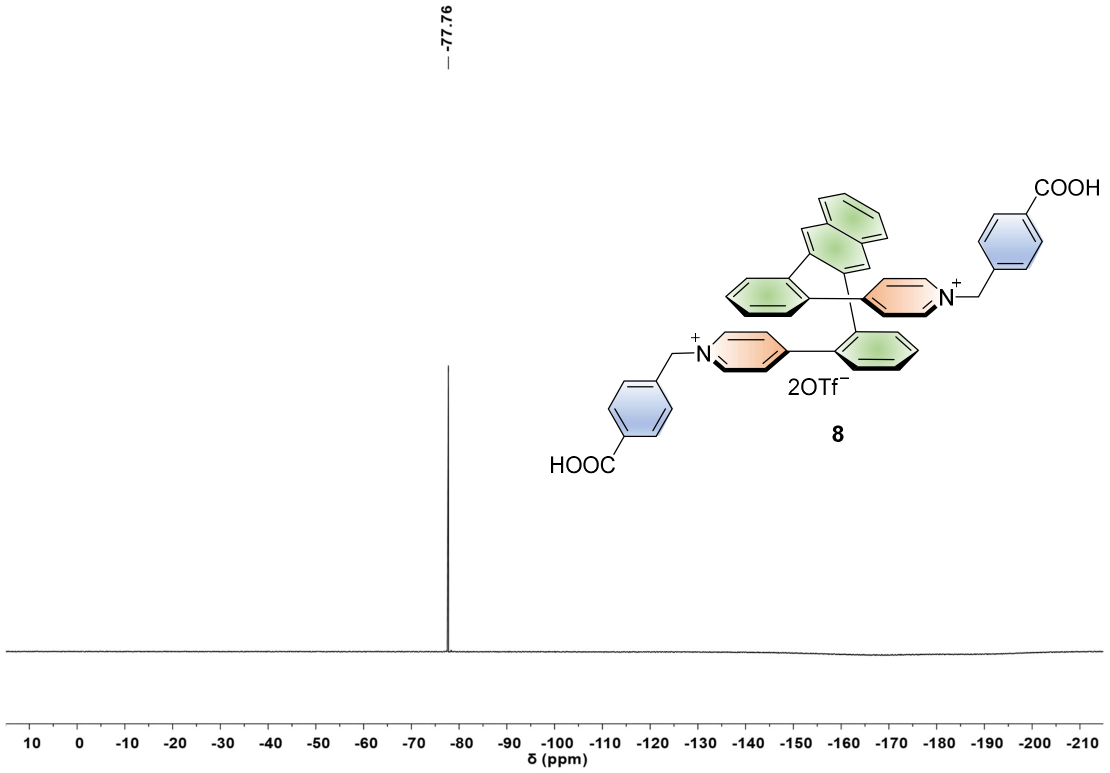

HRMS of **8**.

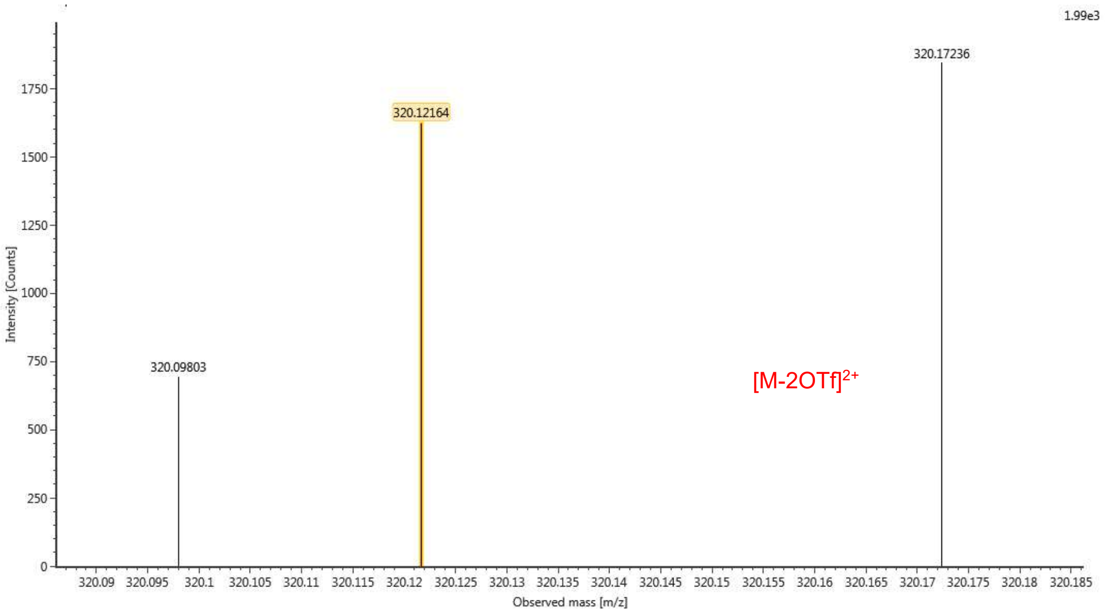
1.99e3
320.17236
320.12164
320.09803
[M-2OTf]2+
Intensity [Counts]
Observed mass [m/z]

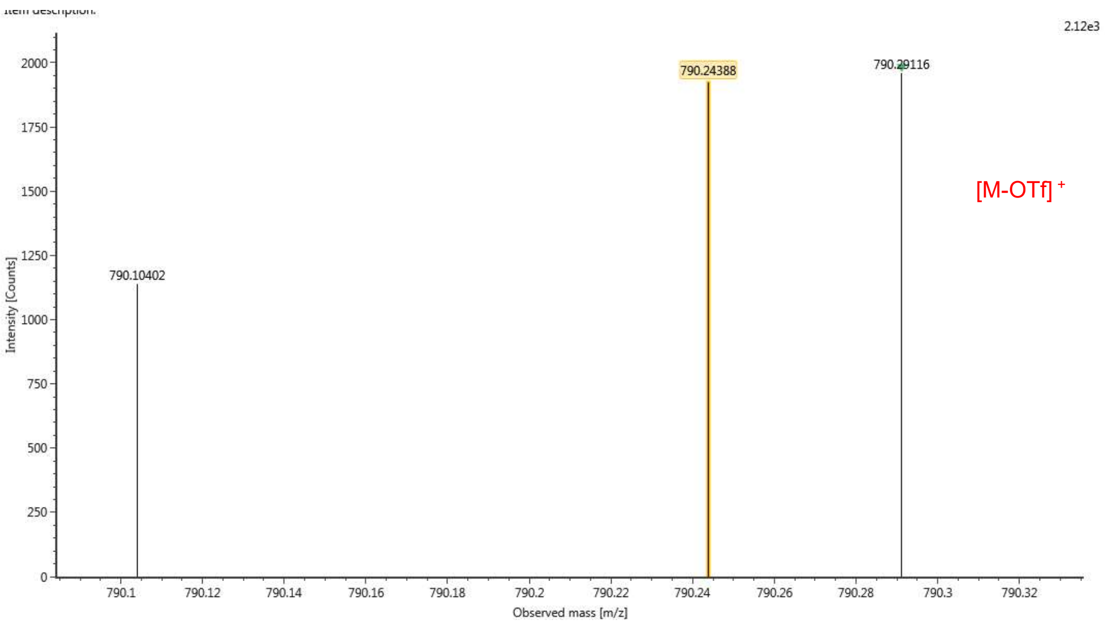
2.12e3
790.24388
790.29116
790.10402
[M-OTf] +
Intensity [Counts]
Observed mass [m/z]

$^1$H NMR spectrum (400 MHz, $CD_3CN$) of **9.**

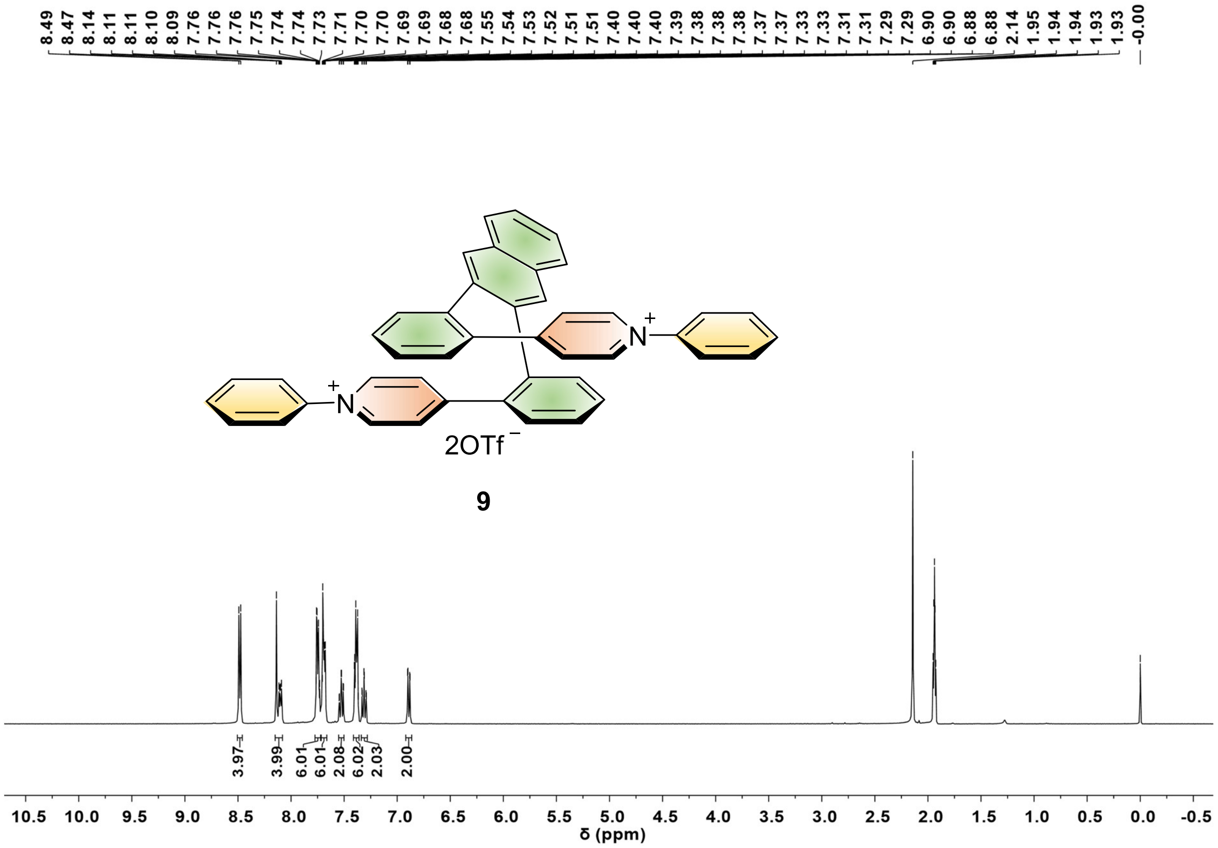

$^1$H-$^1$H COSY spectrum (400 MHz, $CD_3CN$) of **9.**

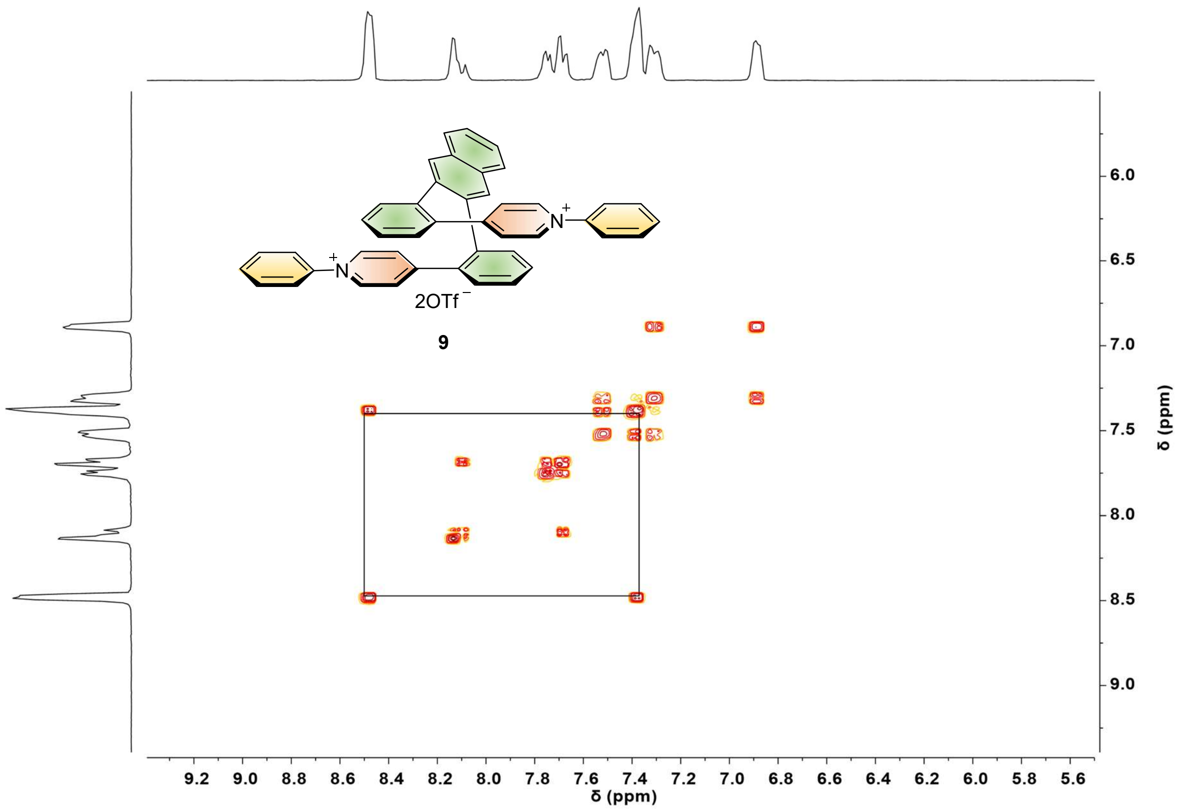

$^{13}C$ NMR spectrum (100 MHz, $CD_3CN$) of **9.**

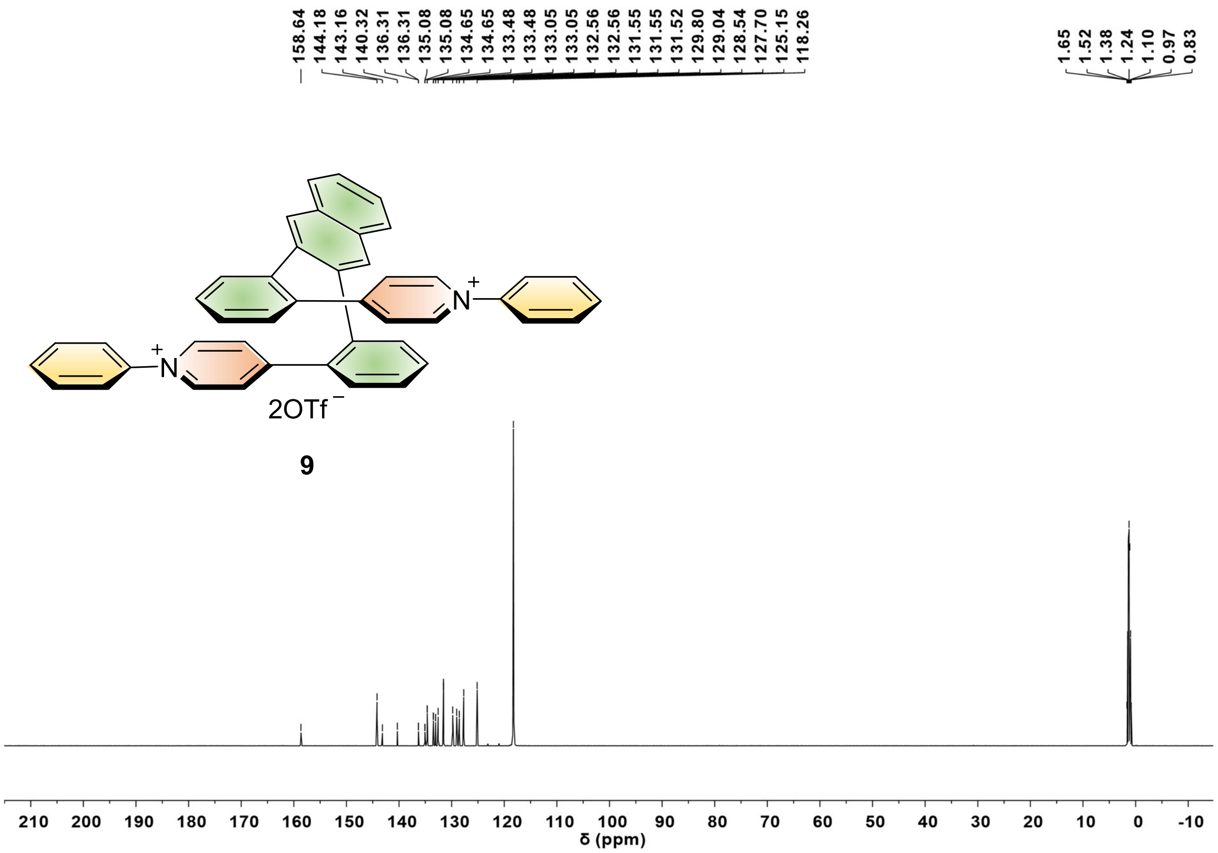


$^{19}F$ NMR spectrum (376 MHz, DMSO-$d_6$) of **9.**

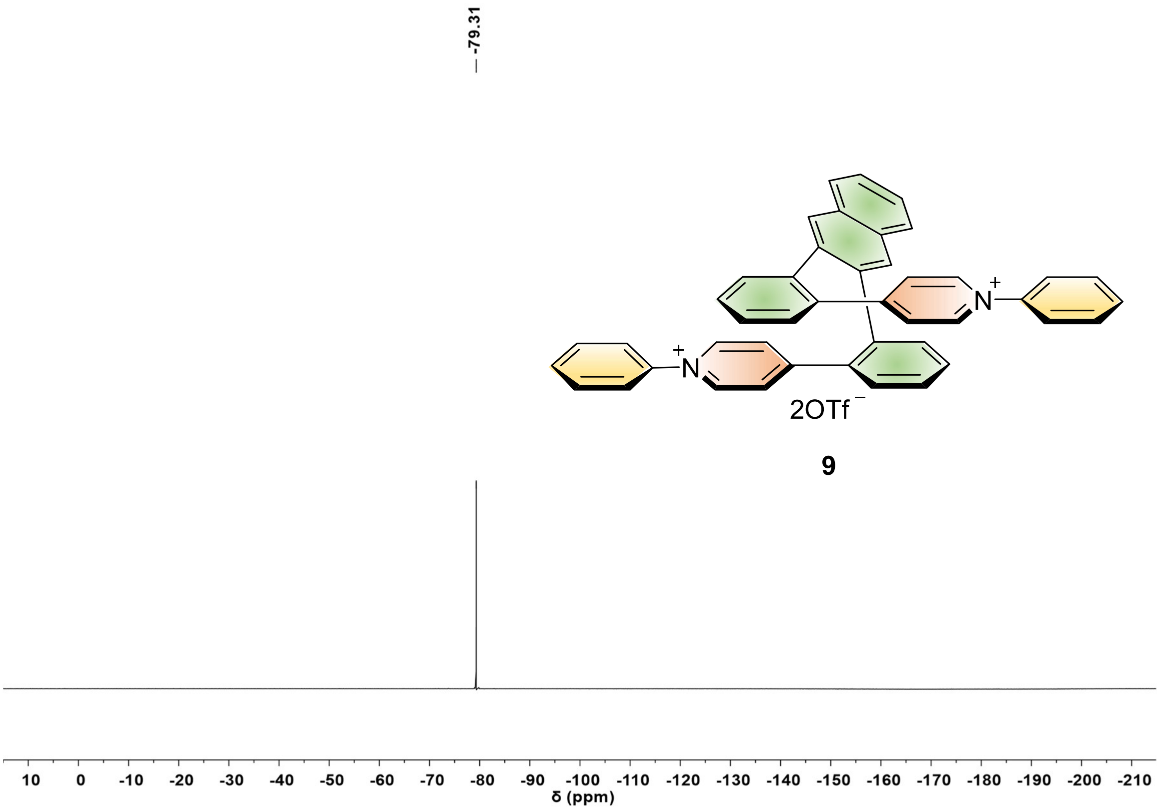

HRMS of **9**.

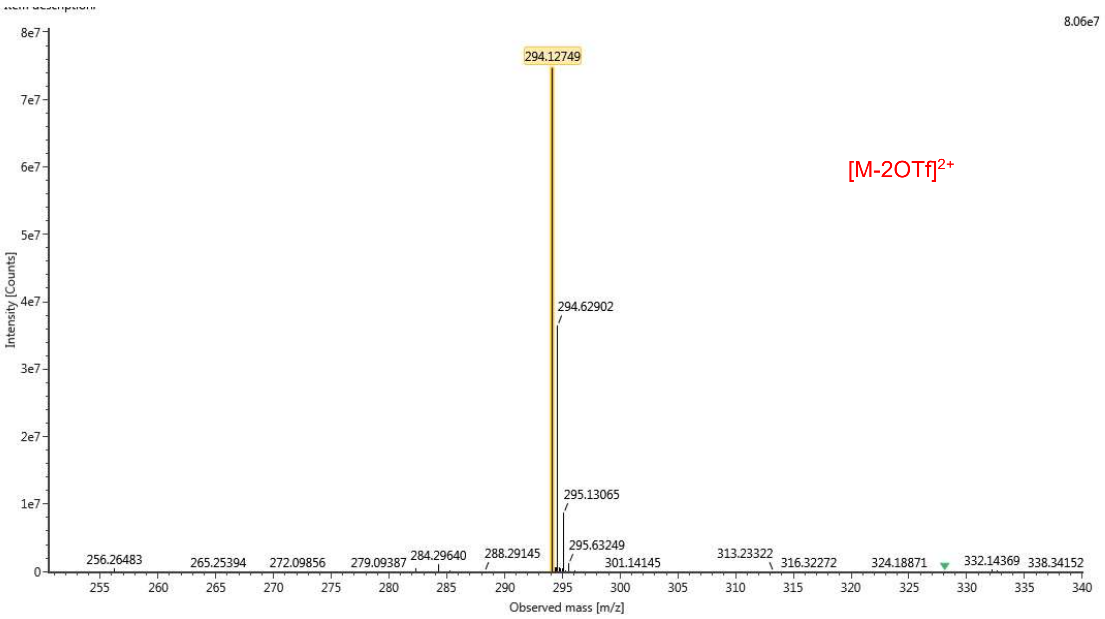
8.06e7
294.12749
[M-2OTf]2+
294.62902
295.13065
295.63249
256.26483
265.25394
272.09856
279.09387
284.29640
288.29145
301.14145
313.23322
316.32272
324.18871
332.14369
338.34152
Intensity [Counts]
Observed mass [m/z]

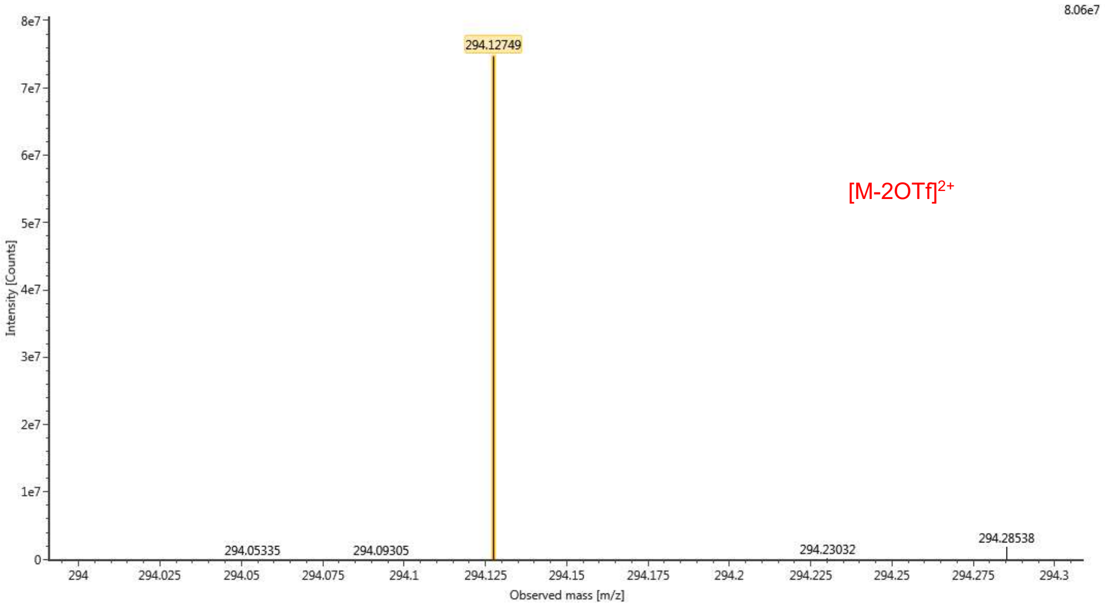
8.06e7
294.12749
[M-2OTf]2+
294.05335
294.09305
294.23032
294.28538
Intensity [Counts]
Observed mass [m/z]

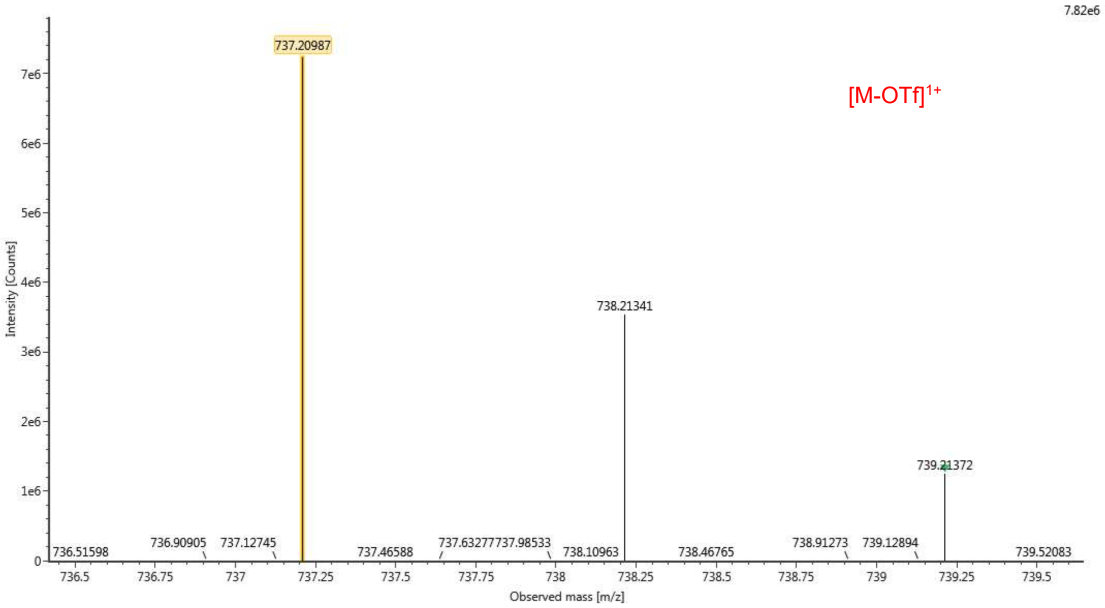
7.82e6
[M-OTf]$^{1+}$
737.20987
738.21341
739.21372
736.51598
736.90905
737.12745
737.46588
737.63277737.98533
738.10963
738.46765
738.91273
739.12894
739.52083
Intensity [Counts]
Observed mass [m/z]

**References**

[1] B. He, S. Zhang, Y. Zhang, G. Li, B. Zhang, W. Ma, B. Rao, R. Song, L. Zhang, Y. Zhang, G. He, *J. Am. Chem. Soc.* **2022**, *144*, 4422.
[2] S. Zhang, L. Ma, W. Ma, L. Chen, K. Gao, S. Yu, M. Zhang, L. Zhang, G. He, *Angew. Chem. Int. Ed.* **2022**, *61*, e202209054.
