# Supplementary material for: Donor–Acceptor Viologens with Through‐Space Conjugation for Enhanced Visible‐Light‐Driven Photocatalysis
Source: Adv Sci (Weinh). 2024 Nov 21;12(2):2409925. doi: 10.1002/advs.202409925 (PMC11727243; doi:10.1002/advs.202409925)

checkCIF/PLATON report

Structure factors have been supplied for datablock(s) 240223lu_lgpz205726_0m

THIS REPORT IS FOR GUIDANCE ONLY. IF USED AS PART OF A REVIEW PROCEDURE FOR PUBLICATION, IT SHOULD NOT REPLACE THE EXPERTISE OF AN EXPERIENCED CRYSTALLOGRAPHIC REFEREE.

No syntax errors found. CIF dictionary Interpreting this report

Datablock: 240223lu_lgpz205726_0m

Bond precision:	C-C = 0.0055 A	Wavelength=0.71073	
Cell:	a=13.7758(8)	b=22.2531(11)	c=14.1250(6)
	alpha=90	beta=100.094(2)	gamma=90
Temperature:	193 K		

	Calculated	Reported
Volume	4263.1(4)	4263.1(4)
Space group	P 21/n	P 1 21/n 1
Hall group	-P 2yn	-P 2yn
Moiety formula	C44 H32 N2, 2(C F3 O3 S), C2 H3 N	2(C F3 O3 S), C44 H32 N2, C2 H3 N
Sum formula	C48 H35 F6 N3 O6 S2	C48 H35 F6 N3 O6 S2
Mr	927.91	927.91
Dx,g cm-3	1.446	1.446
Z	4	4
Mu (mm-1)	0.206	0.206
F000	1912.0	1912.0
F000’	1914.11	
h,k,lmax	16,26,17	16,26,17
Nref	7809	7808
Tmin,Tmax	0.976,0.984	0.629,0.746
Tmin’	0.976	

Correction method= # Reported T Limits: Tmin=0.629 Tmax=0.746
AbsCorr = MULTI-SCAN

Data completeness= 1.000 Theta(max)= 25.349

R(reflections)= 0.0632(4516) wR2(reflections)= 0.1364(7808)

S = 1.038 Npar= 587

The following ALERTS were generated. Each ALERT has the format

test-name_ALERT_alert-type_alert-level.

Click on the hyperlinks for more details of the test.

Alert level C

```
PLAT042_ALERT_1_C Calc. and Reported MoietyFormula Strings  Differ     Please Check
              Calc: C44 H32 N2, 2(C F3 O3 S), C2 H3 N
              Rep.: 2(C F3 O3 S), C44 H32 N2, C2 H3 N
PLAT244_ALERT_4_C Low    'Solvent' Ueq as Compared to Neighbors of        S2 Check
PLAT244_ALERT_4_C Low    'Solvent' Ueq as Compared to Neighbors of       C46 Check
PLAT340_ALERT_3_C Low Bond Precision on  C-C Bonds ...............    0.00554 Ang.
PLAT906_ALERT_3_C Large K Value in the Analysis of Variance ......     11.280 Check
```

Alert level G

```
PLAT244_ALERT_4_G Low    'Solvent' Ueq as Compared to Neighbors of       C47 Check
PLAT244_ALERT_4_G Low    'Solvent' Ueq as Compared to Neighbors of       C48 Check
PLAT432_ALERT_2_G Short Inter X...Y Contact  O6       ..C11      .      3.00 Ang.
                                                     x,y,z  =      1_555 Check
PLAT910_ALERT_3_G Missing # of FCF Reflection(s) Below Theta(Min).         1 Note
                0  1  1,
PLAT933_ALERT_2_G Number of HKL-OMIT Records in Embedded .res File         1 Note
                0  1  1,
PLAT941_ALERT_3_G Average HKL Measurement Multiplicity ..........        4.3 Low
PLAT969_ALERT_5_G The 'Henn et al.' R-Factor-gap value ..........       1.96 Note
              Predicted wR2: Based on SigI**2  6.98 or SHELX Weight 13.67
PLAT978_ALERT_2_G Number C-C Bonds with Positive Residual Density.         2 Info
```

0 **ALERT level A** = Most likely a serious problem - resolve or explain
0 **ALERT level B** = A potentially serious problem, consider carefully
5 **ALERT level C** = Check. Ensure it is not caused by an omission or oversight
8 **ALERT level G** = General information/check it is not something unexpected

1 ALERT type 1 CIF construction/syntax error, inconsistent or missing data
3 ALERT type 2 Indicator that the structure model may be wrong or deficient
4 ALERT type 3 Indicator that the structure quality may be low
4 ALERT type 4 Improvement, methodology, query or suggestion
1 ALERT type 5 Informative message, check

It is advisable to attempt to resolve as many as possible of the alerts in all categories. Often the minor alerts point to easily fixed oversights, errors and omissions in your CIF or refinement strategy, so attention to these fine details can be worthwhile. In order to resolve some of the more serious problems it may be necessary to carry out additional measurements or structure refinements. However, the purpose of your study may justify the reported deviations and the more serious of these should normally be commented upon in the discussion or experimental section of a paper or in the "special_details" fields of the CIF. checkCIF was carefully designed to identify outliers and unusual parameters, but every test has its limitations and alerts that are not important in a particular case may appear. Conversely, the absence of alerts does not guarantee there are no aspects of the results needing attention. It is up to the individual to critically assess their own results and, if necessary, seek expert advice.

Publication of your CIF in IUCr journals

A basic structural check has been run on your CIF. These basic checks will be run on all CIFs submitted for publication in IUCr journals (*Acta Crystallographica*, *Journal of Applied Crystallography*, *Journal of Synchrotron Radiation*); however, if you intend to submit to *Acta Crystallographica Section C* or *E* or *IUCrData*, you should make sure that full publication checks are run on the final version of your CIF prior to submission.

Publication of your CIF in other journals

Please refer to the *Notes for Authors* of the relevant journal for any special instructions relating to CIF submission.

PLATON version of 06/01/2024; check.def file version of 05/01/2024

Datablock 240223lu_lgpz205726_0m - ellipsoid plot

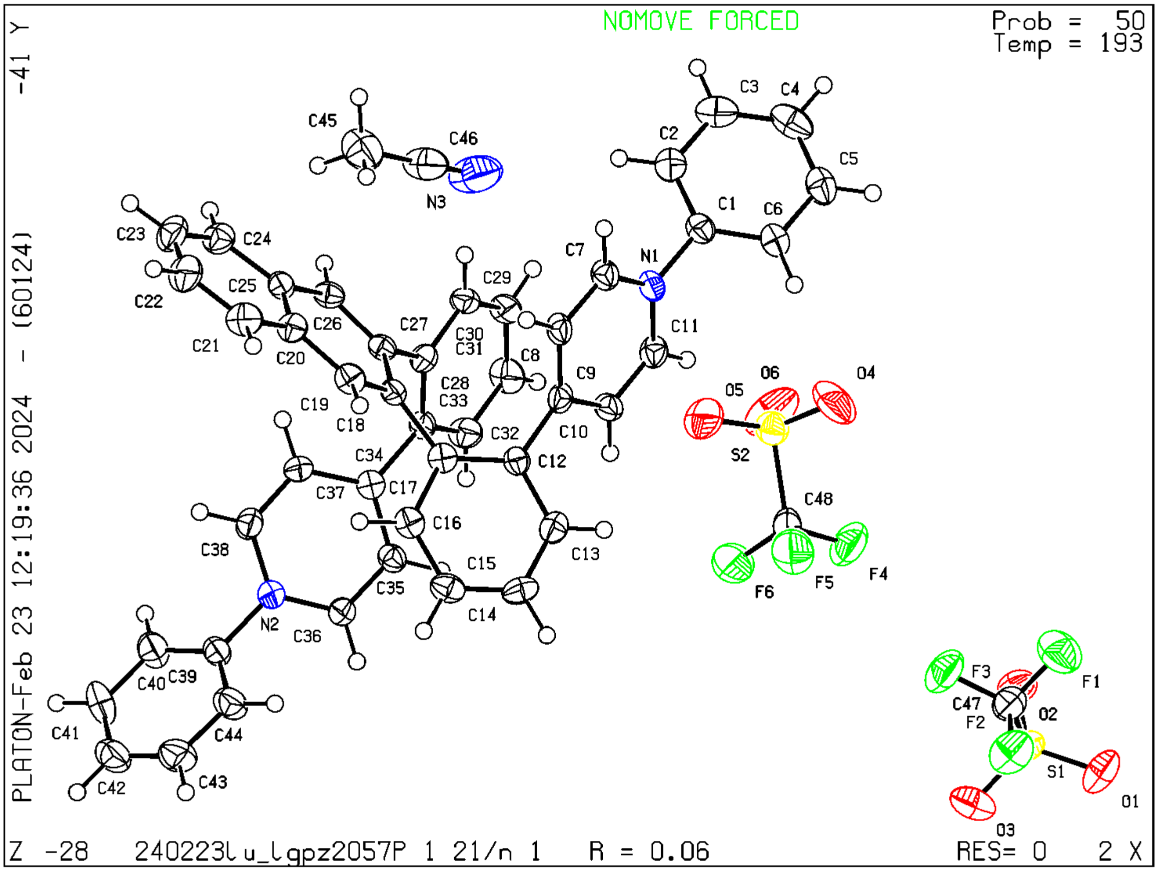

Supplement: Supplementary file 2 — Supporting cif [file ADVS-12-2409925-s002.zip › 9+checkcif.pdf]
