# Supplementary material for: Donor–Acceptor Viologens with Through‐Space Conjugation for Enhanced Visible‐Light‐Driven Photocatalysis
Source: Adv Sci (Weinh). 2024 Nov 21;12(2):2409925. doi: 10.1002/advs.202409925 (PMC11727243; doi:10.1002/advs.202409925)

checkCIF/PLATON report

Structure factors have been supplied for datablock(s) qy4

THIS REPORT IS FOR GUIDANCE ONLY. IF USED AS PART OF A REVIEW PROCEDURE FOR PUBLICATION, IT SHOULD NOT REPLACE THE EXPERTISE OF AN EXPERIENCED CRYSTALLOGRAPHIC REFEREE.

No syntax errors found. CIF dictionary Interpreting this report

Datablock: qy4

Bond precision: C-C = 0.0030 A Wavelength=1.54178

Cell:	a=10.3901(2)	b=12.4184(3)	c=13.5201(3)
	alpha=68.642(1)	beta=82.927(1)	gamma=69.479(1)
Temperature:	100 K		

	Calculated	Reported
Volume	1521.56(6)	1521.56(6)
Space group	P -1	P -1
Hall group	-P 1	-P 1
Moiety formula	C28 H24 N2 S, 2(C F3 O3 S)	C28 H24 N2 S, 2(C F3 O3 S)
Sum formula	C30 H24 F6 N2 O6 S3	C30 H24 F6 N2 O6 S3
Mr	718.69	718.69
Dx,g cm-3	1.569	1.569
Z	2	2
Mu (mm-1)	2.994	2.994
F000	736.0	736.0
F000'	740.60	
h,k,lmax		12,15,16
Nref		5766
Tmin,Tmax	0.670,0.698	0.657,0.753
Tmin'	0.608	

Correction method= # Reported T Limits: Tmin=0.657 Tmax=0.753
AbsCorr = MULTI-SCAN

Data completeness= Theta(max)= 70.282

R(reflections)= 0.0431(4978) wR2(reflections)= 0.1094(5766)

S = 1.071 Npar= 426

The following ALERTS were generated. Each ALERT has the format
test-name_ALERT_alert-type_alert-level.
Click on the hyperlinks for more details of the test.

Alert level C

```
PLAT094_ALERT_2_C Ratio of Maximum / Minimum Residual Density ....       2.15 Report
PLAT911_ALERT_3_C Missing FCF Refl Between Thmin & STh/L=    0.600         15 Report
                5  4  0,   2  2  1,   0  3  1,   2  3  1,   2  3  2,   3  3  2,
                4  4  2,   3  3  3,   3  5  3,   3-10  4,   4  5  4,  10 12  6,
               10 12  7,  -6 -6 10,   1  5 16,
PLAT975_ALERT_2_C Check Calcd Resid. Dens.  0.97Ang From O1      .       0.58 eA-3
```

Alert level G

```
PLAT154_ALERT_1_G The s.u.'s on the Cell Angles are Equal ..(Note)      0.001 Degree
PLAT244_ALERT_4_G Low    'Solvent' Ueq as Compared to Neighbors of        C29 Check
PLAT244_ALERT_4_G Low    'Solvent' Ueq as Compared to Neighbors of        C30 Check
PLAT720_ALERT_4_G Number of Unusual/Non-Standard Labels .........           1 Note
              S02
PLAT883_ALERT_1_G No Info/Value for _atom_sites_solution_primary .     Please Do !
PLAT912_ALERT_4_G Missing # of FCF Reflections Above STh/L=  0.600          9 Note
PLAT913_ALERT_3_G Missing # of Very Strong Reflections in FCF ....          1 Note
                0  3  1,
PLAT978_ALERT_2_G Number C-C Bonds with Positive Residual Density.          7 Info
```

0 **ALERT level A** = Most likely a serious problem - resolve or explain
0 **ALERT level B** = A potentially serious problem, consider carefully
3 **ALERT level C** = Check. Ensure it is not caused by an omission or oversight
8 **ALERT level G** = General information/check it is not something unexpected

2 ALERT type 1 CIF construction/syntax error, inconsistent or missing data
3 ALERT type 2 Indicator that the structure model may be wrong or deficient
2 ALERT type 3 Indicator that the structure quality may be low
4 ALERT type 4 Improvement, methodology, query or suggestion
0 ALERT type 5 Informative message, check

It is advisable to attempt to resolve as many as possible of the alerts in all categories. Often the minor alerts point to easily fixed oversights, errors and omissions in your CIF or refinement strategy, so attention to these fine details can be worthwhile. In order to resolve some of the more serious problems it may be necessary to carry out additional measurements or structure refinements. However, the purpose of your study may justify the reported deviations and the more serious of these should normally be commented upon in the discussion or experimental section of a paper or in the "special_details" fields of the CIF. checkCIF was carefully designed to identify outliers and unusual parameters, but every test has its limitations and alerts that are not important in a particular case may appear. Conversely, the absence of alerts does not guarantee there are no aspects of the results needing attention. It is up to the individual to critically assess their own results and, if necessary, seek expert advice.

Publication of your CIF in IUCr journals

A basic structural check has been run on your CIF. These basic checks will be run on all CIFs submitted for publication in IUCr journals (*Acta Crystallographica*, *Journal of Applied Crystallography*, *Journal of Synchrotron Radiation*); however, if you intend to submit to *Acta Crystallographica Section C* or *E* or *IUCrData*, you should make sure that full publication checks are run on the final version of your CIF prior to submission.

Publication of your CIF in other journals

Please refer to the *Notes for Authors* of the relevant journal for any special instructions relating to CIF submission.

PLATON version of 29/11/2023; check.def file version of 14/09/2023

Datablock qy4 - ellipsoid plot

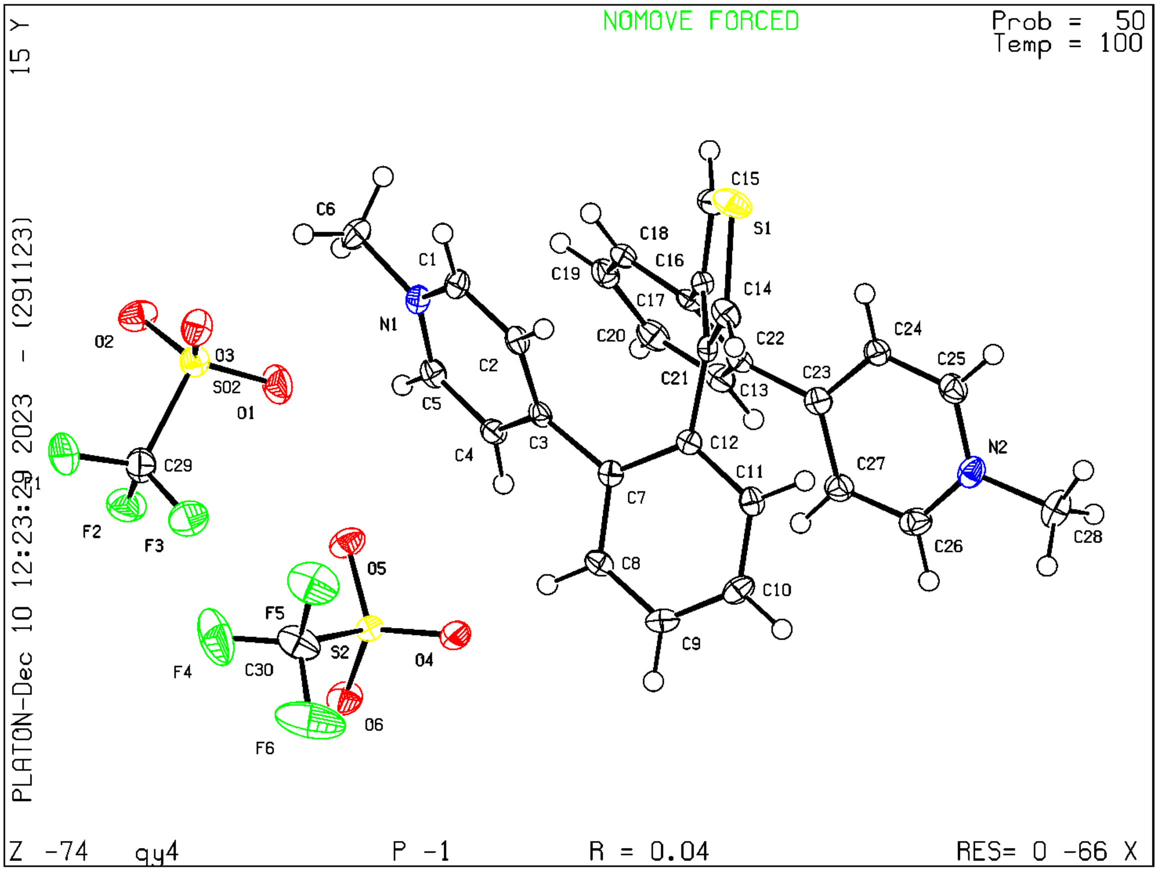

Supplement: Supplementary file 2 — Supporting cif [file ADVS-12-2409925-s002.zip › 3+checkcif .pdf]
