# Supplementary material for: Donor–Acceptor Viologens with Through‐Space Conjugation for Enhanced Visible‐Light‐Driven Photocatalysis
Source: Adv Sci (Weinh). 2024 Nov 21;12(2):2409925. doi: 10.1002/advs.202409925 (PMC11727243; doi:10.1002/advs.202409925)

checkCIF/PLATON report

Structure factors have been supplied for datablock(s) qy-2

THIS REPORT IS FOR GUIDANCE ONLY. IF USED AS PART OF A REVIEW PROCEDURE FOR PUBLICATION, IT SHOULD NOT REPLACE THE EXPERTISE OF AN EXPERIENCED CRYSTALLOGRAPHIC REFEREE.

No syntax errors found. CIF dictionary Interpreting this report

Datablock: qy-2

Bond precision:	C-C = 0.0033 A		Wavelength=0.71073
Cell:	a=9.1938(4)	b=39.441(2)	c=13.2161(7)
	alpha=90	beta=96.804(2)	gamma=90
Temperature:	150 K		

	Calculated	Reported
Volume	4758.6(4)	4758.6(4)
Space group	P 21/c	P 1 21/c 1
Hall group	-P 2ybc	-P 2ybc
Moiety formula	C32 H22 N2 [+ solvent]	C32 H22 N2
Sum formula	C32 H22 N2 [+ solvent]	C32 H22 N2
Mr	434.52	434.56
Dx,g cm-3	1.213	1.213
Z	8	8
Mu (mm-1)	0.071	0.071
F000	1824.0	1824.0
F000'	1824.64	
h,k,lmax	11,51,17	11,51,17
Nref	10910	10885
Tmin,Tmax	0.989,0.991	0.641,0.746
Tmin'	0.989	

Correction method= # Reported T Limits: Tmin=0.641 Tmax=0.746
AbsCorr = MULTI-SCAN

Data completeness= 0.998 Theta(max)= 27.499

R(reflections)= 0.0699(7618) wR2(reflections)= 0.1882(10885)

S = 1.041 Npar= 613

The following ALERTS were generated. Each ALERT has the format
test-name_ALERT_alert-type_alert-level.
Click on the hyperlinks for more details of the test.

Alert level C

```
PLAT230_ALERT_2_C Hirshfeld Test Diff for    N2       --C32      .         6.0 s.u.
PLAT230_ALERT_2_C Hirshfeld Test Diff for    N3       --C52      .         6.0 s.u.
PLAT230_ALERT_2_C Hirshfeld Test Diff for    C35      --C36      .         6.0 s.u.
PLAT906_ALERT_3_C Large K Value in the Analysis of Variance ......      4.956 Check
```

Alert level G

```
PLAT605_ALERT_4_G Largest Solvent Accessible VOID in the Structure        201 A**3
PLAT868_ALERT_4_G ALERTS Due to the Use of _smtbx_masks Suppressed          ! Info
PLAT910_ALERT_3_G Missing # of FCF Reflection(s) Below Theta(Min).          3 Note
PLAT912_ALERT_4_G Missing # of FCF Reflections Above STh/L=  0.600         23 Note
PLAT933_ALERT_2_G Number of HKL-OMIT Records in Embedded .res File          2 Note
PLAT941_ALERT_3_G Average HKL Measurement Multiplicity ...........        4.9 Low
PLAT978_ALERT_2_G Number C-C Bonds with Positive Residual Density.         21 Info
```

0 **ALERT level A** = Most likely a serious problem - resolve or explain
0 **ALERT level B** = A potentially serious problem, consider carefully
4 **ALERT level C** = Check. Ensure it is not caused by an omission or oversight
7 **ALERT level G** = General information/check it is not something unexpected

0 ALERT type 1 CIF construction/syntax error, inconsistent or missing data
5 ALERT type 2 Indicator that the structure model may be wrong or deficient
3 ALERT type 3 Indicator that the structure quality may be low
3 ALERT type 4 Improvement, methodology, query or suggestion
0 ALERT type 5 Informative message, check

It is advisable to attempt to resolve as many as possible of the alerts in all categories. Often the minor alerts point to easily fixed oversights, errors and omissions in your CIF or refinement strategy, so attention to these fine details can be worthwhile. In order to resolve some of the more serious problems it may be necessary to carry out additional measurements or structure refinements. However, the purpose of your study may justify the reported deviations and the more serious of these should normally be commented upon in the discussion or experimental section of a paper or in the "special_details" fields of the CIF. checkCIF was carefully designed to identify outliers and unusual parameters, but every test has its limitations and alerts that are not important in a particular case may appear. Conversely, the absence of alerts does not guarantee there are no aspects of the results needing attention. It is up to the individual to critically assess their own results and, if necessary, seek expert advice.

Publication of your CIF in IUCr journals

A basic structural check has been run on your CIF. These basic checks will be run on all CIFs submitted for publication in IUCr journals (*Acta Crystallographica*, *Journal of Applied Crystallography*, *Journal of Synchrotron Radiation*); however, if you intend to submit to *Acta Crystallographica Section C* or *E* or *IUCrData*, you should make sure that full publication checks are run on the final version of your CIF prior to submission.

Publication of your CIF in other journals

Please refer to the *Notes for Authors* of the relevant journal for any special instructions relating to CIF submission.

PLATON version of 06/07/2023; check.def file version of 30/06/2023

Datablock qy-2 - ellipsoid plot

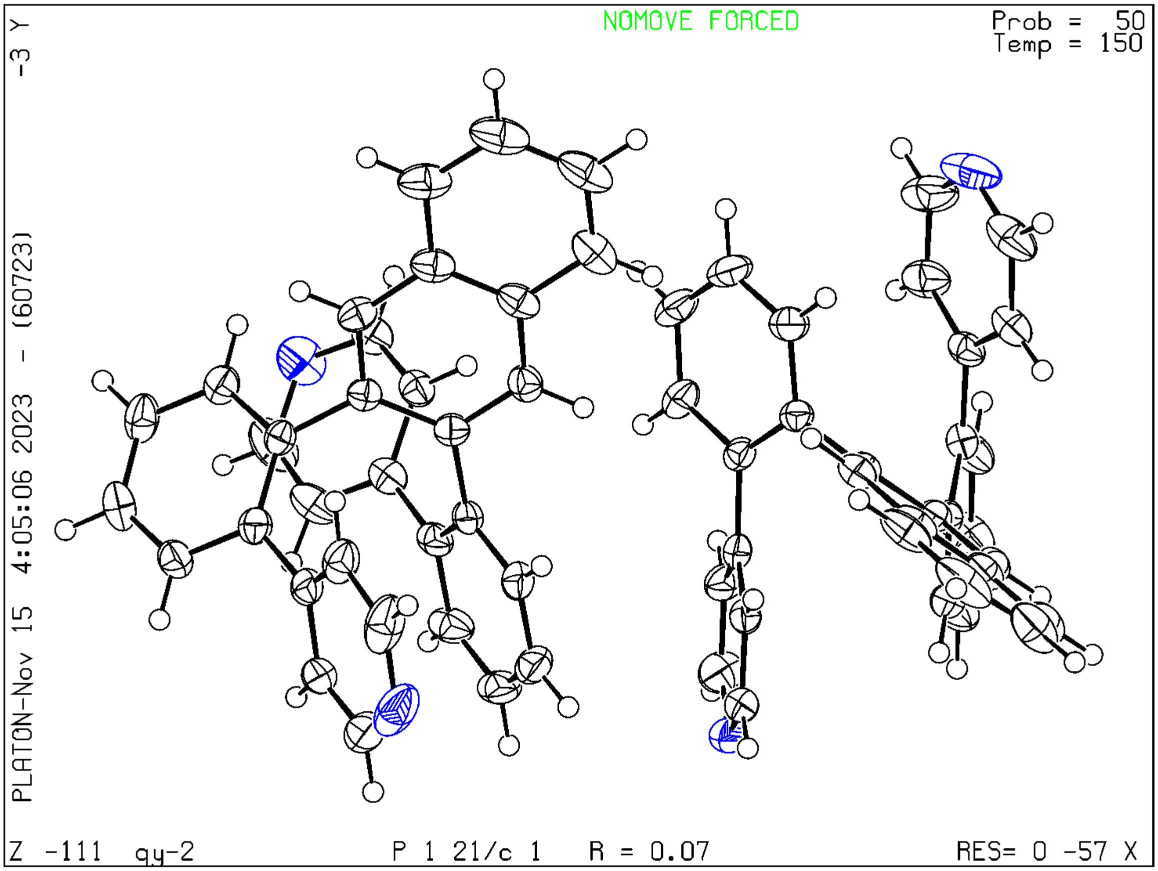

Supplement: Supplementary file 2 — Supporting cif [file ADVS-12-2409925-s002.zip › 6+checkcif.pdf]
