# Supplementary material for: Donor–Acceptor Viologens with Through‐Space Conjugation for Enhanced Visible‐Light‐Driven Photocatalysis
Source: Adv Sci (Weinh). 2024 Nov 21;12(2):2409925. doi: 10.1002/advs.202409925 (PMC11727243; doi:10.1002/advs.202409925)

checkCIF/PLATON report

Structure factors have been supplied for datablock(s) 240223lu_lgpz205726_1_0m

THIS REPORT IS FOR GUIDANCE ONLY. IF USED AS PART OF A REVIEW PROCEDURE FOR PUBLICATION, IT SHOULD NOT REPLACE THE EXPERTISE OF AN EXPERIENCED CRYSTALLOGRAPHIC REFEREE.

No syntax errors found. CIF dictionary Interpreting this report

Datablock: 240223lu_lgpz205726_1_0m

Bond precision:	C-C = 0.0075 A		Wavelength=1.34139
Cell:	a=13.747(4)	b=14.267(6)	c=27.719(8)
	alpha=76.775(14)	beta=81.439(8)	gamma=88.527(10)
Temperature:	193 K		

	Calculated	Reported
Volume	5233(3)	5233(3)
Space group	P -1	P -1
Hall group	-P 1	-P 1
Moiety formula	2(C48 H36 N2 O4), 4(C F3 O3 S), C2 H3 N [+ solvent]	4(C F3 O3 S), 2(C48 H36 N2 O4), C2 H3 N
Sum formula	C102 H75 F12 N5 O20 S4 [+ solvent]	C102 H75 F12 N5 O20 S4
Mr	2046.91	2046.91
Dx,g cm-3	1.299	1.299
Z	2	2
Mu (mm-1)	1.042	1.042
F000	2108.0	2108.0
F000’	2116.07	
h,k,lmax	16,17,33	16,17,33
Nref	19152	19001
Tmin,Tmax	0.882,0.920	0.612,0.752
Tmin’	0.882	

Correction method= # Reported T Limits: Tmin=0.612 Tmax=0.752
AbsCorr = MULTI-SCAN

Data completeness= 0.992 Theta(max)= 53.906

R(reflections)= 0.1260(12887)

wR2(reflections)= 0.3152(19001)

S = 1.065

Npar= 1434

The following ALERTS were generated. Each ALERT has the format
test-name_ALERT_alert-type_alert-level.
Click on the hyperlinks for more details of the test.

Alert level C

```
PLAT082_ALERT_2_C High R1 Value .................................       0.13 Report
PLAT084_ALERT_3_C High wR2 Value (i.e. > 0.25) ...................       0.32 Report
PLAT234_ALERT_4_C Large Hirshfeld Difference S2       --O13      .       0.21 Ang.
PLAT234_ALERT_4_C Large Hirshfeld Difference S2       --C100     .       0.16 Ang.
PLAT234_ALERT_4_C Large Hirshfeld Difference F6       --C100     .       0.17 Ang.
PLAT244_ALERT_4_C Low    'Solvent' Ueq as Compared to Neighbors of         S1 Check
PLAT244_ALERT_4_C Low    'Solvent' Ueq as Compared to Neighbors of         S2 Check
PLAT244_ALERT_4_C Low    'Solvent' Ueq as Compared to Neighbors of       C103 Check
PLAT250_ALERT_2_C Large U3/U1 Ratio for <U(i,j)> Tensor(Resd    5)        2.4 Note
PLAT250_ALERT_2_C Large U3/U1 Ratio for <U(i,j)> Tensor(Resd    7)        2.3 Note
PLAT260_ALERT_2_C Large Average Ueq of Residue Including        S2      0.169 Check
PLAT260_ALERT_2_C Large Average Ueq of Residue Including        S3      0.160 Check
PLAT260_ALERT_2_C Large Average Ueq of Residue Including        S4      0.149 Check
PLAT260_ALERT_2_C Large Average Ueq of Residue Including        S5      0.190 Check
PLAT260_ALERT_2_C Large Average Ueq of Residue Including       S4A      0.139 Check
PLAT340_ALERT_3_C Low Bond Precision on  C-C Bonds ..............    0.00748 Ang.
PLAT790_ALERT_4_C Centre of Gravity not Within Unit Cell: Resd.  #          1 Note
              C48 H36 N2 O4
PLAT906_ALERT_3_C Large K Value in the Analysis of Variance ......      3.993 Check
PLAT911_ALERT_3_C Missing FCF Refl Between Thmin & STh/L=    0.600        149 Report
                2  0  0,   3  0  0,  -2  2  0,  -1  4  0,   0  4  0,   1  4  0,
               -7 10  0,   4 12  0,   7-11  1,   4-10  1,   1 -4  1,  -1 -2  1,
                1 -2  1,   2 -2  1,  -3  0  1,  -4  2  1,   1  2  1,  -7 10  1,
                5 14  1,   3-10  2,   5-10  2,   6-10  2,  10-10  2,   0 -4  2,
                3 -4  2,   0  0  2,   1  0  2,   1  2  2,  -2  4  2,   1  4  2,
                2  4  2,   6 14  2,   1-16  3,   3-10  3,   4-10  3,   5-10  3,
                8-10  3,  -2 -2  3,   0 -2  3,   2  0  3,   3  0  3,  -4  2  3,
               -3  2  3,  -2  2  3,  -1  2  3,   1  2  3,   1  4  3,   4 14  3,
               -5-12  4,  -3-12  4,   3-10  4,   2 -2  4,   0  0  4,   2  0  4,
                3  0  4,   0  2  4,   3  2  4,  -2  4  4,  -1  4  4,   0-14  5,
                4-10  5,  -1 -2  5,  -2  0  5,   2  0  5,  -3  2  5,  -2  2  5,
               -1  2  5,   0  2  5,   1  4  5,   2  4  5,   3 16  5,  -2-12  6,
               -2-10  6,   3-10  6,  -1  0  6,   1  4  6, -10  8  6,  -6  8  6,
                1-14  7,  -1  2  7,  -9  8  7,  -8  8  7,  -7  8  7,  -3-12  8,
               -2-12  8,  -1-10  8,   1-10  8, -15  2  8,   1-13  9,   2-13  9,
                1-12  9,   0-11  9,  -2-10  9,   1-10  9,   0  0  9, -15  2  9,
PLAT913_ALERT_3_C Missing # of Very Strong Reflections in FCF ....         22 Note
                2  0  0,   0  4  0,   1  4  0,   1 -4  1,  -1 -2  1,  -3  0  1,
               -4  2  1,   0 -4  2,   1  0  2,   3  0  3,  -4  2  3,  -3  2  3,
               -2  2  3,   1  4  3,   0  0  4,   2  0  4,   2  0  5,  -3  2  5,
               -2  2  5,  -1  2  5,   1  4  5,   2  4  5,
PLAT918_ALERT_3_C Reflection(s) with I(obs) much Smaller I(calc) .          6 Check
PLAT975_ALERT_2_C Check Calcd Resid. Dens.  0.77Ang From O23     .       0.87 eA-3
```

Alert level G

```
ABSMU01_ALERT_1_G  Calculation of _exptl_absorpt_correction_mu
                not performed for this radiation type.
PLAT002_ALERT_2_G Number of Distance or Angle Restraints on AtSite         36 Note
PLAT003_ALERT_2_G Number of Uiso or Uij Restrained non-H Atoms ...         40 Report
PLAT007_ALERT_5_G Number of Unrefined Donor-H Atoms .............           8 Report
              H1    H2    H3    H4    H5    H6    H7    H8
PLAT042_ALERT_1_G Calc. and Reported MoietyFormula Strings  Differ     Please Check
              Calc: 2(C48 H36 N2 O4), 4(C F3 O3 S), C2 H3 N
              Rep.: 4(C F3 O3 S), 2(C48 H36 N2 O4), C2 H3 N
PLAT083_ALERT_2_G SHELXL Second Parameter in WGHT  Unusually Large      20.00 Why ?
PLAT172_ALERT_4_G The CIF-Embedded .res File Contains DFIX Records          9 Report
PLAT173_ALERT_4_G The CIF-Embedded .res File Contains DANG Records         18 Report
PLAT175_ALERT_4_G The CIF-Embedded .res File Contains SAME Records          1 Report
PLAT176_ALERT_4_G The CIF-Embedded .res File Contains SADI Records         19 Report
PLAT178_ALERT_4_G The CIF-Embedded .res File Contains SIMU Records          6 Report
PLAT186_ALERT_4_G The CIF-Embedded .res File Contains ISOR Records          1 Report
PLAT187_ALERT_4_G The CIF-Embedded .res File Contains RIGU Records          3 Report
PLAT188_ALERT_3_G A Non-default SIMU Restraint Value has been used     0.0100 Report
PLAT188_ALERT_3_G A Non-default SIMU Restraint Value has been used     0.0100 Report
PLAT188_ALERT_3_G A Non-default SIMU Restraint Value has been used     0.0100 Report
PLAT188_ALERT_3_G A Non-default SIMU Restraint Value has been used     0.0100 Report
PLAT191_ALERT_3_G A Non-default SADI Restraint Value has been used     0.0400 Report
PLAT191_ALERT_3_G A Non-default SADI Restraint Value has been used     0.0400 Report
PLAT191_ALERT_3_G A Non-default SADI Restraint Value has been used     0.0400 Report
PLAT191_ALERT_3_G A Non-default SADI Restraint Value has been used     0.0400 Report
PLAT191_ALERT_3_G A Non-default SADI Restraint Value has been used     0.0400 Report
PLAT191_ALERT_3_G A Non-default SADI Restraint Value has been used     0.0400 Report
PLAT191_ALERT_3_G A Non-default SADI Restraint Value has been used     0.0400 Report
PLAT191_ALERT_3_G A Non-default SADI Restraint Value has been used     0.0400 Report
PLAT191_ALERT_3_G A Non-default SADI Restraint Value has been used     0.0400 Report
PLAT191_ALERT_3_G A Non-default SADI Restraint Value has been used     0.0400 Report
PLAT191_ALERT_3_G A Non-default SADI Restraint Value has been used     0.0400 Report
PLAT191_ALERT_3_G A Non-default SADI Restraint Value has been used     0.0400 Report
PLAT231_ALERT_4_G Hirshfeld Test (Solvent)   S4       --C98      .        6.8 s.u.
PLAT244_ALERT_4_G Low    'Solvent' Ueq as Compared to Neighbors of        C97 Check
PLAT244_ALERT_4_G Low    'Solvent' Ueq as Compared to Neighbors of       C100 Check
PLAT300_ALERT_4_G Atom Site Occupancy of H1        Constrained at         0.5 Check
PLAT300_ALERT_4_G Atom Site Occupancy of H2        Constrained at         0.5 Check
PLAT300_ALERT_4_G Atom Site Occupancy of H3        Constrained at         0.5 Check
PLAT300_ALERT_4_G Atom Site Occupancy of H4        Constrained at         0.5 Check
PLAT300_ALERT_4_G Atom Site Occupancy of H5        Constrained at         0.5 Check
PLAT300_ALERT_4_G Atom Site Occupancy of H6        Constrained at         0.5 Check
PLAT300_ALERT_4_G Atom Site Occupancy of H7        Constrained at         0.5 Check
PLAT300_ALERT_4_G Atom Site Occupancy of H8        Constrained at         0.5 Check
PLAT300_ALERT_4_G Atom Site Occupancy of S3        Constrained at         0.5 Check
PLAT300_ALERT_4_G Atom Site Occupancy of F10       Constrained at         0.5 Check
PLAT300_ALERT_4_G Atom Site Occupancy of F11       Constrained at         0.5 Check
PLAT300_ALERT_4_G Atom Site Occupancy of F12       Constrained at         0.5 Check
PLAT300_ALERT_4_G Atom Site Occupancy of O18       Constrained at         0.5 Check
PLAT300_ALERT_4_G Atom Site Occupancy of O19       Constrained at         0.5 Check
PLAT300_ALERT_4_G Atom Site Occupancy of O20       Constrained at         0.5 Check
PLAT300_ALERT_4_G Atom Site Occupancy of C99       Constrained at         0.5 Check
PLAT300_ALERT_4_G Atom Site Occupancy of S5        Constrained at         0.5 Check
PLAT300_ALERT_4_G Atom Site Occupancy of F13       Constrained at         0.5 Check
PLAT300_ALERT_4_G Atom Site Occupancy of F14       Constrained at         0.5 Check
PLAT300_ALERT_4_G Atom Site Occupancy of F15       Constrained at         0.5 Check
```

```
PLAT300_ALERT_4_G Atom Site Occupancy of O21       Constrained at        0.5 Check
PLAT300_ALERT_4_G Atom Site Occupancy of O22       Constrained at        0.5 Check
PLAT300_ALERT_4_G Atom Site Occupancy of O23       Constrained at        0.5 Check
PLAT300_ALERT_4_G Atom Site Occupancy of C101      Constrained at        0.5 Check
PLAT302_ALERT_4_G Anion/Solvent/Minor-Residue Disorder (Resd    5)       100% Note
PLAT302_ALERT_4_G Anion/Solvent/Minor-Residue Disorder (Resd    6)       100% Note
PLAT302_ALERT_4_G Anion/Solvent/Minor-Residue Disorder (Resd    7)       100% Note
PLAT302_ALERT_4_G Anion/Solvent/Minor-Residue Disorder (Resd    8)       100% Note
PLAT304_ALERT_4_G Non-Integer Number of Atoms in ..... (Resd    6)       4.92 Check
PLAT304_ALERT_4_G Non-Integer Number of Atoms in ..... (Resd    8)       3.08 Check
PLAT432_ALERT_2_G Short Inter X...Y Contact  C32      ..C61      .       3.20 Ang.
                                                     x,y,z  =      1_555 Check
PLAT434_ALERT_2_G Short Inter HL..HL Contact F5       ..F9A      .       2.76 Ang.
                                                  x,-1+y,z  =      1_545 Check
PLAT606_ALERT_4_G Solvent Accessible VOID(S) in Structure ........          ! Info
PLAT789_ALERT_4_G Atoms with Negative _atom_site_disorder_group  #         16 Check
PLAT790_ALERT_4_G Centre of Gravity not Within Unit Cell: Resd.  #          2 Note
              C48 H36 N2 O4
PLAT790_ALERT_4_G Centre of Gravity not Within Unit Cell: Resd.  #          3 Note
              C F3 O3 S
PLAT790_ALERT_4_G Centre of Gravity not Within Unit Cell: Resd.  #          4 Note
              C F3 O3 S
PLAT790_ALERT_4_G Centre of Gravity not Within Unit Cell: Resd.  #          5 Note
              C F3 O3 S
PLAT790_ALERT_4_G Centre of Gravity not Within Unit Cell: Resd.  #          6 Note
              C F3 O3 S
PLAT790_ALERT_4_G Centre of Gravity not Within Unit Cell: Resd.  #          7 Note
              C F3 O3 S
PLAT790_ALERT_4_G Centre of Gravity not Within Unit Cell: Resd.  #          8 Note
              C F3 O3 S
PLAT790_ALERT_4_G Centre of Gravity not Within Unit Cell: Resd.  #          9 Note
              C2 H3 N
PLAT822_ALERT_4_G CIF-embedded .res Contains Negative PART Numbers          2 Check
PLAT860_ALERT_3_G Number of Least-Squares Restraints ............         676 Note
PLAT868_ALERT_4_G ALERTS Due to the Use of _smtbx_masks Suppressed          ! Info
PLAT912_ALERT_4_G Missing # of FCF Reflections Above STh/L=  0.600          2 Note
PLAT941_ALERT_3_G Average HKL Measurement Multiplicity ..........         4.0 Low
PLAT969_ALERT_5_G The 'Henn et al.' R-Factor-gap value ..........        4.53 Note
              Predicted wR2: Based on SigI**2  6.95 or SHELX Weight 30.79
PLAT978_ALERT_2_G Number C-C Bonds with Positive Residual Density.          0 Info
```

```
   0 ALERT level A = Most likely a serious problem - resolve or explain
   0 ALERT level B = A potentially serious problem, consider carefully
  22 ALERT level C = Check. Ensure it is not caused by an omission or oversight
  81 ALERT level G = General information/check it is not something unexpected

   2 ALERT type 1 CIF construction/syntax error, inconsistent or missing data
  15 ALERT type 2 Indicator that the structure model may be wrong or deficient
  24 ALERT type 3 Indicator that the structure quality may be low
  60 ALERT type 4 Improvement, methodology, query or suggestion
   2 ALERT type 5 Informative message, check
```

It is advisable to attempt to resolve as many as possible of the alerts in all categories. Often the minor alerts point to easily fixed oversights, errors and omissions in your CIF or refinement strategy, so attention to these fine details can be worthwhile. In order to resolve some of the more serious problems it may be necessary to carry out additional measurements or structure refinements. However, the purpose of your study may justify the reported deviations and the more serious of these should normally be commented upon in the discussion or experimental section of a paper or in the "special_details" fields of the CIF. checkCIF was carefully designed to identify outliers and unusual parameters, but every test has its limitations and alerts that are not important in a particular case may appear. Conversely, the absence of alerts does not guarantee there are no aspects of the results needing attention. It is up to the individual to critically assess their own results and, if necessary, seek expert advice.

Publication of your CIF in IUCr journals

A basic structural check has been run on your CIF. These basic checks will be run on all CIFs submitted for publication in IUCr journals (*Acta Crystallographica*, *Journal of Applied Crystallography*, *Journal of Synchrotron Radiation*); however, if you intend to submit to *Acta Crystallographica Section C* or *E* or *IUCrData*, you should make sure that full publication checks are run on the final version of your CIF prior to submission.

Publication of your CIF in other journals

Please refer to the *Notes for Authors* of the relevant journal for any special instructions relating to CIF submission.

PLATON version of 06/01/2024; check.def file version of 05/01/2024

Datablock 240223lu_lgpz205726_1_0m - ellipsoid plot

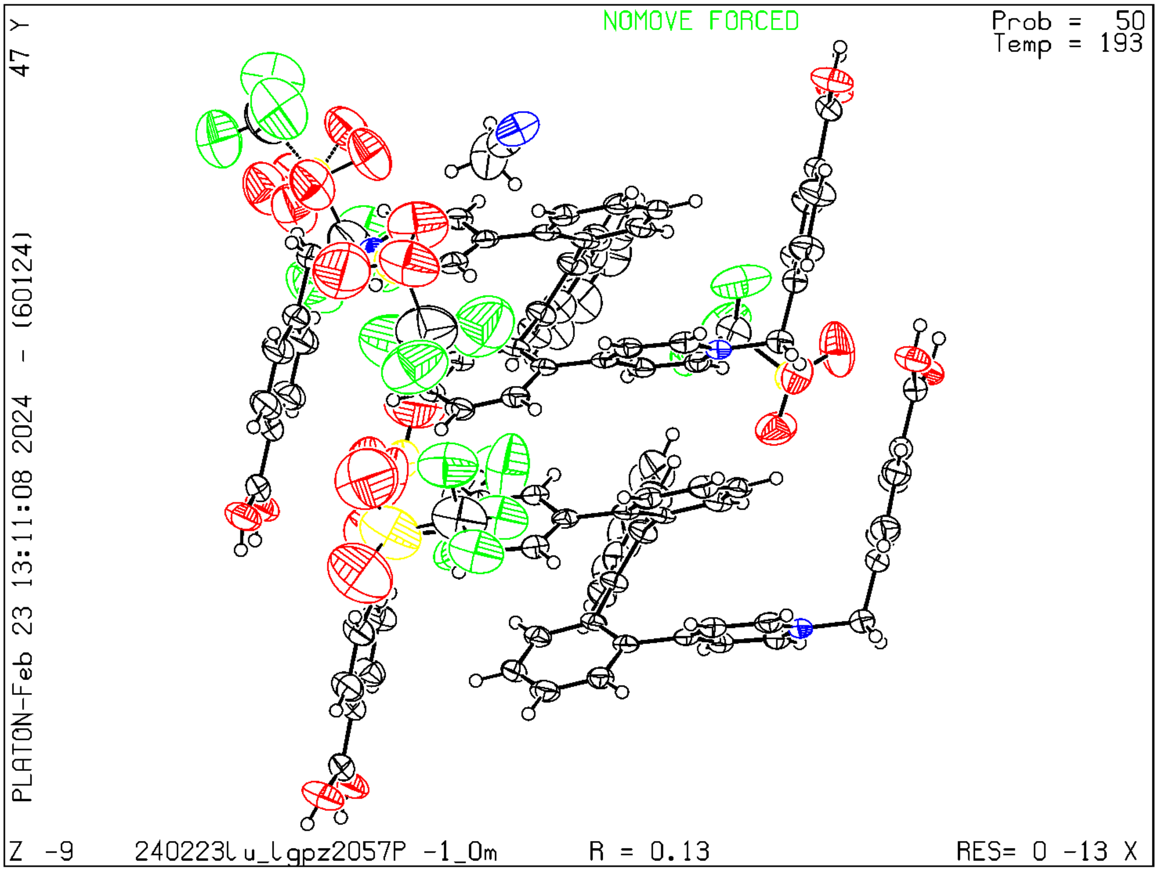

Supplement: Supplementary file 2 — Supporting cif [file ADVS-12-2409925-s002.zip › 8+checkcif.pdf]
